# Specificity and exon target space of splicing modifying compounds

**DOI:** 10.1038/s41467-026-74594-9

**Published:** 2026-07-04

**Authors:** Felina Lenkeit, Judith Knehr, Marc Altorfer, Andrea Byrnes, Wenjing Li, Jack Hsiao, Connie Wu, Priti Gaitonde, Philip R. Skaanderup, Steve Mullin, Elizaveta Solovyeva, Michal Pikusa, Andrew T. Krueger, Johannes Ottl, Caroline Gubser Keller, Christian Kolter, Ulrike Naumann, Philipp Ottis, Alejandro Reyes

**Affiliations:** 1 Discovery Sciences, Novartis Biomedical Research, Basel, Switzerland; 2 Discovery Sciences, Novartis Biomedical Research, Cambridge, MA USA; 3 Disease Area Neuroscience, Novartis Biomedical Research, Cambridge, MA USA; 4 Research Informatics, Novartis Biomedical Research, Basel, Switzerland

**Keywords:** Gene expression analysis, Target identification, Alternative splicing

## Abstract

Modulation of splicing is an established therapeutic strategy with clinical applications and potential to target specific exons to influence gene expression. Small-molecule splicing modifiers such as Risdiplam and Branaplam induce inclusion of exons typically skipped due to weak ${5}^{{\prime} }$ splice sites. Risdiplam preferentially induces exons with an N_−3_G_−2_A_−1_ sequence at the ${3}^{{\prime} }$ exon end, whereas Branaplam favors A_−3_G_−2_A_−1_-ending exons. However, determinants of specificity remain unclear, as many motif-matching exons are not induced. Here, we investigate the molecular basis of splicing-modulator specificity. Using biochemical assays, transcriptome analyses, and genetic perturbations, we identify sequence-dependent features that determine exon responsiveness to splicing-modulator induction. We further demonstrate that specificity can be reprogrammed through manipulation of U1 snRNA. These findings refine the determinants of splicing-modulator target space and may support identification of additional target exons and compounds.

## Abstract (web summary)

Splicing modifiers show selective exon targeting, but the basis of specificity is unclear. Here, the authors identify sequence- and structure-based determinants, including U1 snRNA complementarity, that govern exon responsiveness and demonstrate that splicing-modulator specificity can be reprogrammed.

## Introduction

**Fig. 1:**
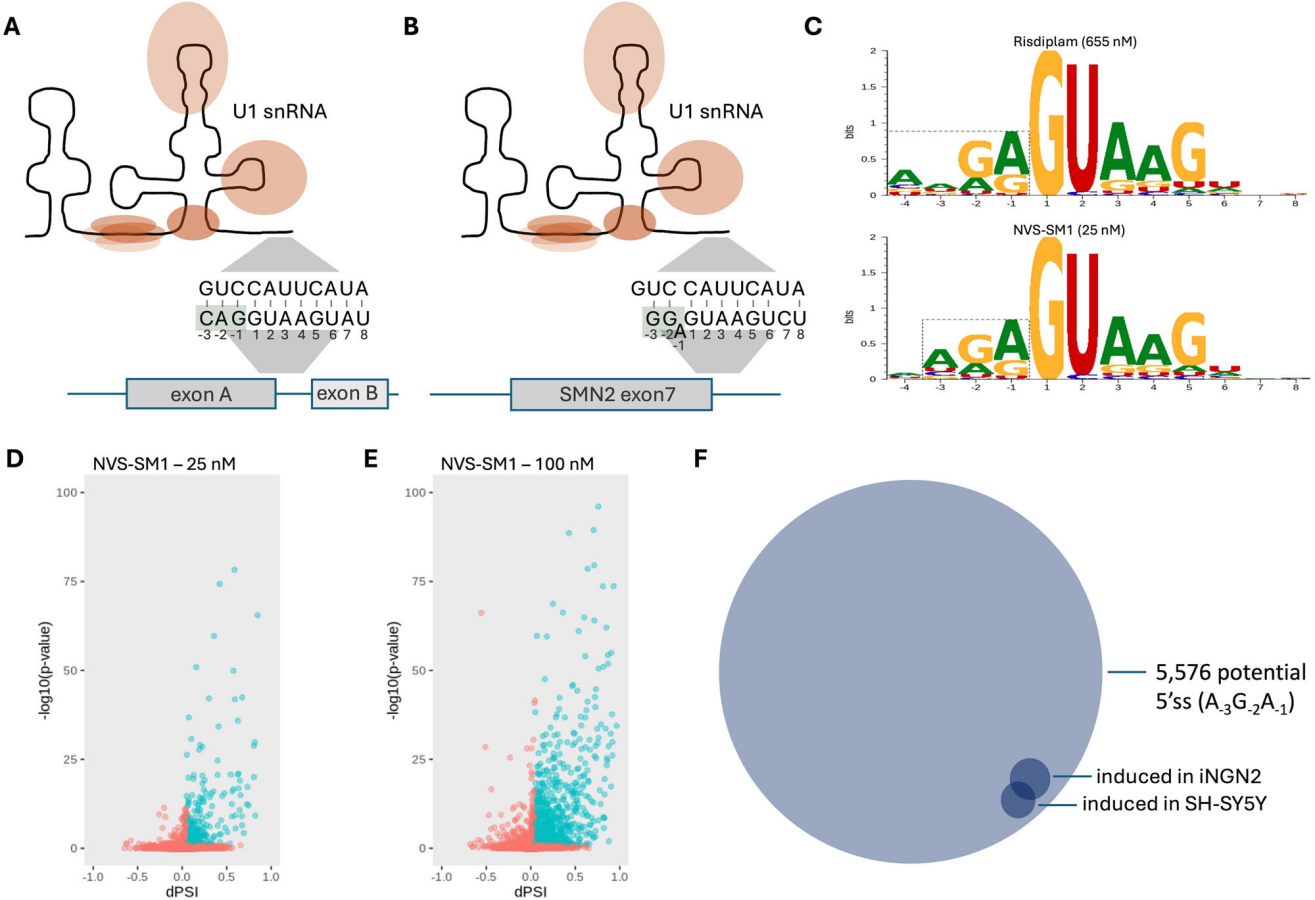
Sequence specificity of splicing compounds. **A** Splice site recognition by base-pairing between the U1 snRNA within the spliceosome and an exon carrying a consensus U1 binding site (here called exon A). The U1 binding site spans from the three nucleotides at the ${3}^{{\prime} }$ end of exon (positions -3, -2, -1) to the eight nucleotides of the downstream intron (positions +1 to +8). **B** Splice site recognition of exon 7 in the *SMN2* gene in the presence of a splicing modifier like Risdiplam. **C** RNA sequence logos to represent sequence conservation at the U1 binding site of all exons included in response to either 655 nM Risdiplam, or 25 nM NVS-SM1. The last 4 nucleotides of the exon (position −4 to −1) and the first 8 nucleotides of the following intron (position +1 to +8) are included in the logo. **D** Differential splicing analysis between whole transcriptome profiles of SH-SY5Y cells treated with 25 nM NVS-SM1 and SH-SY5Y cells treated with DMSO. Exons highlighted in turquoise are considered as included in response to compound treatment (ΔPSI  > 0.05, adjusted *p* < 0.1). Statistical significance was assessed using a likelihood ratio test, and adjusted *p* values are reported. Source data are provided as a Source Data file. **E** Differential splicing analysis between whole transcriptome profiles of SH-SY5Y cells treated with 100 nM NVS-SM1 and SH-SY5Y cells treated with DMSO. Statistical significance was assessed using a likelihood ratio test, and adjusted *p* values are reported. Source data are provided as a Source Data file. **F** Venn diagram to highlight gap between 5576 potential ${5}^{{\prime} }$ ss matching primary sequence preference of NVS-SM1 and the actual number of detected induced AGA-ending exons in response to NVS-SM1 treatment in SH-SY5Y and iNGN2 cells.

Splicing is a fundamental biological process that generates mature mRNA by removing introns from pre-mRNA. Splicing is carried out by the spliceosome, a macromolecular complex which comprises various protein components and small nuclear RNAs (snRNAs), together termed small ribonucleoproteins (snRNPs). One of these snRNAs, as part of the U1 snRNP, guides the first step of the splicing reaction by base-pairing with the sequence spanning from the terminal three nucleotides at the ${3}^{{\prime} }$ end of an exon (positions -3, -2, -1) to the eight nucleotides of the downstream intron (positions +1 to +8) ^1,2^ (Fig. 1A). This sequence stretch in the pre-mRNAs is known as U1 binding site and its consensus sequence is ${5}^{{\prime} }-{{{\rm{C}}}}_{-3}{{{\rm{A}}}}_{-2}{{{\rm{G}}}}_{-1}{{{\rm{G}}}}_{1}{{{\rm{U}}}}_{2}{{{\rm{A}}}}_{3}{{{\rm{A}}}}_{4}{{{\rm{G}}}}_{5}{{{\rm{U}}}}_{6}{{{\rm{A}}}}_{7}{{{\rm{U}}}}_{8}-{3}^{{\prime} }$. Similarly, the branchpoint sequence, the polypyrimidine tract and the ${3}^{{\prime} }$ splice site (${3}^{{\prime} }$ ss) are sequence elements that guide the splicing machinery to determine the ${5}^{{\prime} }$ start of an exon. In most human genes, alternative selection of ${5}^{{\prime} }$ and ${3}^{{\prime} }$ splice sites enable the production of multiple isoforms from a single gene, contributing to protein diversity^3,4^.

Modification of splice site selection has been proven to be a promising approach in treating various diseases. This strategy addresses not only diseases caused by mutations that lead to aberrant splicing, but also diseases with other causes, where modulating the splicing process can influence the expression levels of particular isoforms^5^. By directly targeting mRNAs, this approach enables the modulation of proteins that may otherwise be challenging to drug or even considered undruggable. While antisense oligonucleotides (ASOs) constitute the majority of clinically validated splicing modifiers^6–8^, RNA-targeting small molecules offer numerous advantages. Notably, these small molecules allow non-invasive oral delivery, ensure systemic distribution, and have the capacity to cross the blood-brain barrier, thus enhancing their potential as therapeutic agents^9,10^.

To date, there are several small molecule splicing modulator compounds with available human clinical data, including Risdiplam, NVS-SM1 (Branaplam)^11^, PTC518, SKY-0515, RGT-61159 and REM-422. Risdiplam is being used for the treatment of spinal muscular atrophy (SMA)^12^. SMA is caused by a mutation in the survival motor neuron 1 (*SMN1*) gene that leads to a deficiency of functional SMN proteins^13^. The gene *SMN2* is nearly identical to *SMN1*, but harbors a critical C to T substitution in exon 7 that disrupts an exonic splicing enhancer and impairs exon inclusion. This sequence context leads to inefficient recognition of exon 7, resulting in predominant exon skipping and production of non-functional *SMN2* isoforms (Fig. 1B). By introducing exon 7 into transcripts of *SMN2*, Risdiplam restores the expression of functional SMN protein that compensates for the *SMN1* pathologic mutations^14–16^. Branaplam, here referred to as its development code NVS-SM1, was also found to promote exon 7 inclusion in *SMN2* transcripts. Additionally, this molecule demonstrated the capability to induce the inclusion of a frame-shifting pseudoexon between exon 49 and 50 in the *HTT* gene that introduces a pre-mature stop codon which triggers the mRNA to be degraded^17–19^. This *HTT* pseudoexon is not recognized by the spliceosome in the absence of NVS-SM1, as it contains a non-canonical ${5}^{{\prime} }$ ss (${5}^{{\prime} }-{{{\rm{A}}}}_{-3}{{{\rm{G}}}}_{-2}{{{\rm{A}}}}_{-1}{{{\rm{G}}}}_{1}{{{\rm{U}}}}_{2}{{{\rm{A}}}}_{3}{{{\rm{A}}}}_{4}{{{\rm{G}}}}_{5}{{{\rm{G}}}}_{6}{{{\rm{G}}}}_{7}{{{\rm{G}}}}_{8}-{3}^{{\prime} }$).

Genome-wide transcriptome analyses and mutational screening assays have shown that Risdiplam has a preference to induce exons with a N_−3_G_−2_A_−1_ (N = any base) sequence at the ${3}^{{\prime} }$ exon end, while NVS-SM1 has a proclivity towards introducing A_−3_G_−2_A_−1_-ending exons^20,21^ (Fig. 1C). Without the presence of these compounds, these exons are typically being skipped due to their weak ${5}^{{\prime} }$ ss, making their recognition unlikely^22–24^. An NMR structure of a chemical analog of Risdiplam, SMN-C5, showed that the compound binds the RNA helix formed by the ${5}^{{\prime} }$ ss and the ${5}^{{\prime} }$ end of the U1 snRNA in its major groove. Here, it stabilizes the bulged-out A_−1_ at the exon end, acting like a molecular glue that strengthens the interaction of both RNAs, compensating for the weak ${5}^{{\prime} }$ ss to enable splicing initiation^25,26^. Subsequent NMR analyses extended this bulge-repair mechanism to diverse chemotypes, including Branaplam analogs, confirming that structurally distinct compounds can target the same A_−1_ bulge at the exon-intron junction^27^. Beyond structural models, Ishigami and co-workers applied a quantitative high-throughput splicing assay and modeling approach to demonstrate that, while Risdiplam recognizes a relatively narrow N_−3_G_−2_A_−1_ motif, Branaplam displays two distinct sequence-recognition modes at the ${5}^{{\prime} }$ ss. This suggests that specificity arises from the subtle interplay between local sequence composition and RNA conformational dynamics^21^. Moreover, they identified the -4 position as a key determinant within the A_−4_N_−3_G_−2_A_−1_ motif of Risdiplam-responsive exons. With all analyses performed in a fixed genetic background, where exon architecture, U1 snRNP composition and spliceosomal components remained unaltered, the influence of these characteristics in different local genetic contexts is still unexplored.

While the studies above have substantially contributed to our understanding of small-molecule splicing modulation, the determinants of the underlying selectivity remain incompletely resolved. For instance, a recent study predicted >58,000 potential exons with a ${5}^{{\prime} }$ ss sequence of ${5}^{{\prime} }-{{{\rm{A}}}}_{-1}{{{\rm{G}}}}_{-2}{{{\rm{A}}}}_{-3}{{{\rm{G}}}}_{1}{{{\rm{U}}}}_{2}{{{\rm{A}}}}_{3}{{{\rm{A}}}}_{4}{{{\rm{G}}}}_{5}-{3}^{{\prime} }$ in human introns, however observed inclusion upon NVS-SM1 treatment for only 609 exons^17^. Similarly, transcriptome data have shown that N_−3_G_−2_A_−1_ ending exons are induced at different levels upon treatment with NVS-SM1 or Risdiplam.

Here, we analyzed the contribution of sequence elements to the specificity of splicing modulators using a combination of biochemical assays, whole transcriptome analyses, and genetic perturbation approaches. In contrast to prior studies focused primarily on the structural mechanism of bulge stabilization, we present quantitative evidence linking sequence complementarity between U1 snRNA and the exon to drug responsiveness. Beyond characterizing exons that are induced by splicing modulators, we also investigated exons that remain unresponsive to treatment in order to reveal sequence features that prevent compound activity. Our analyses suggest that the presence of an inducible A_−1_ bulge is not merely a structural consequence of compound binding but rather a prerequisite for drug-mediated exon inclusion. To address additional determinants of specificity, we examined whether the conservation of nucleotides in the intronic portion of U1-binding sites among compound-responsive exons arises from direct involvement in compound-RNA interactions or instead reflects their role in stabilizing the U1–${5}^{{\prime} }$ ss duplex. By genetically modifying the U1 snRNP, we demonstrate that sequence complementarity between U1 snRNA and target exons in these intronic nucleotides not only drives sensitivity but can also be exploited to modulate compound specificity. Finally, we systematically integrated sequence context and predicted regulatory elements and developed a machine learning framework to evaluate their contribution to compound responsiveness.

Taken together, these results refine the parameters that define the splicing modulator target space and provide mechanistic insights for the rational design of next-generation splicing-modifying compounds.

## Results

NVS-SM1 is known to preferentially induce the splicing of exons with ${3}^{{\prime} }$ ends consisting of an A_−3_G_−2_A_−1_ trinucleotide (Fig. 1C), which we refer to as AGA exons for simplicity^18^. First, we aimed to define the potential space of AGA target exons throughout the human genome. We used the large collection of human RNA-seq data from the Genotype-Tissue Expression Project available through the snaptron database^28^. We identified 5576 potential ${5}^{{\prime} }$ ss matching a ${5}^{{\prime} }-{{{\rm{A}}}}_{-3}{{{\rm{G}}}}_{-2}{{{\rm{A}}}}_{-1}{{{\rm{G}}}}_{1}{{{\rm{U}}}}_{2}-{3}^{{\prime} }$ motif. Most of the corresponding exons were supported by unannotated exon-exon junctions and, when detected, were included at low levels, confirming that these non-canonical exons are usually not recognized by the splicing machinery under normal physiological conditions.

### Variation of exon target space of NVS-SM1 across cell types

This large number of predicted target exons, however, is in strong contrast to the reported transcriptomic profiles in literature, where a few hundred of AGA exons are reported to be induced in response to splicing modifiers such as NVS-SM1^18,20^. Consistent with these reports, we detected the induction of 125 and 304 AGA-ending exons when we treated SH-SY5Y cells for 24 h with 25 nM and 100 nM of NVS-SM1, respectively, in whole transcriptome profiles (10% false discovery rate [FDR]; Fig. 1D/E). Despite focusing on non-canonical ${5}^{{\prime} }$ ss, we detected 334 AGA exons with high levels of baseline inclusion in the absence of a compound ( > 0.6 percent spliced-in [PSI], Supplementary Fig. 2A). Notably, the induction of these high baseline AGA exons was not further boosted upon compound treatment.

To further refine the AGA exon target space, we sequenced the transcriptomes of SH-SY5Y, as well as iNGN2 cells, in a single experiment, following treatment with 25 nM of NVS-SM1 for 24 h. Compared to DMSO controls, we found 97 induced AGA exons in SH-SY5Y cells and 171 in iNGN2 cells, with 75 of these exons common to both (Fig. 1F). When focusing on the list of genes containing AGA-ending exons induced only in SH-SY5Y cells, these genes, on average, demonstrated a 90-times higher expression in SH-SY5Y, compared to iNGN2 cells, and vice versa (Supplementary Fig. 2B). These observations suggest that a large proportion of AGA exons detected in only one cell type are explained by cell type specific expression of genes containing induceable AGA exons. Indeed, when we filtered the 5,576 potential AGA exons for those harbored by highly expressed genes in SH-SY5Y cells and, at the same time, demonstrating low baseline inclusion in the absence compound, the number of potentially inducible AGA exons in SY-SY5Y cells dropped to 628.

**Fig. 2:**
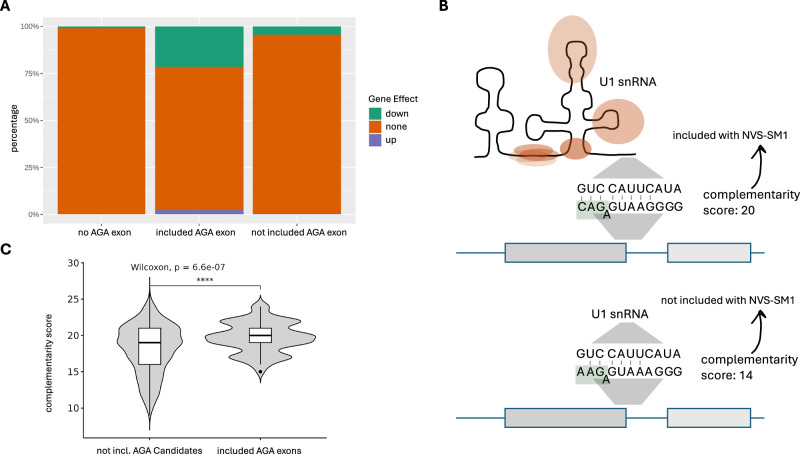
Compound effects on gene expression and U1 snRNA complementarity in exon inclusion. **A** Effect on gene expression (upregulation, downregulation or no effect) of NVS-SM1 treatment of SH-SY5Y cells. 8.5% of the genes carrying an AGA exon, not inducible upon NVS-SM1, are downregulated. Source data are provided as a Source Data file. **B** Example of two AGA exons that seem to be good candidates for NVS-SM1 mediated inclusion due to their primary sequence at the exon end. Here shown as complementarity score is the number of maximal H-bonds between the U1 binding site and the U1 snRNA. **C** Violin plot showing the complementarity between the U1 snRNA and U1 binding sites of AGA-ending exons included in response to NVS-SM1 (included AGA exons, *n* = 125) and those ${5}^{{\prime} }$ ss matching a ${5}^{{\prime} }$ -${{{\rm{A}}}}_{-3}{{{\rm{G}}}}_{-2}{{{\rm{A}}}}_{-1}{{{\rm{G}}}}_{1}{{{\rm{U}}}}_{2}-{3}^{{\prime} }$ motif, thus being potential candidates for NVS-SM1 mediated inclusion, but are not included (not included AGA Candidates, *n* = 1932). The complementarity score was calculated by summing up all H-bonds possibly formed between the two RNAs. Box plots indicate the median (center line), interquartile range (box), whiskers extending to 1.5 × the interquartile range, and individual points beyond this range are shown as outliers. Source data are provided as a Source Data file. Statistical significance was assessed using a two-sided Wilcoxon rank-sum test (*p* = 6.6 × 10^−7^).

Even when considering AGA exons with low baseline exon inclusion and high gene expression (where detectability is not an issue due to low gene expression), approximately half of the potentially inducible AGA exons remained uninduced following NVS-SM1 treatment. We reasoned that if the induction of AGA exons introduced stop codons into transcripts, a rapid degradation of mRNA by nonsense mediated decay (NMD) could prevent their detection by RNA-seq. However, only 8.5% of the genes carrying an uninduced AGA exon were downregulated (Fig. 2A), indicating that this effect would explain only a minority of the unobserved AGA exons. To confirm this observation, we knocked down major RNA decay pathways and analyzed the splicing patterns upon compound treatment. Compared to compound treatment alone, the knocked down cells showed only a tiny increase in AGA exon inclusion (Supplementary Fig. 2C, mean delta PSI $[\overline{\Delta \Psi }]=0.0036$), supporting the notion that RNA degradation does not explain the majority of unobserved AGA exons.

### Impact of U1 snRNA complementarity on compound-mediated exon inclusion

Even when exons do check all the high expression level, low baseline inclusion and exon end motif criteria, some still fail to show inclusion after compound treatment. One endogenous factor governing a ${5}^{{\prime} }$ splice-site’s efficiency is the overall complementarity of this splice-site to the U1 snRNA. High complementarity enhances the recognition of the splice site, whereas low complementarity favors exon skipping^24^. We therefore rationalized that complementarity to U1 snRNA may play a key role in compound-mediated splicing. Even if a modulator helps strengthen weak splice sites, a certain amount of baseline complementarity may still be required. To test this hypothesis, we calculated the maximum number of possible hydrogen bonds (H-bonds) formed between the U1 binding site of AGA exons and the U1 snRNA. Accounting for the possibility of an A_−1_ bulge formation in the pre-mRNA, we included the -4 position of the exon in the score calculation (Fig. 2B). Exemplary scores for some of the exons induced by NVS-SM1 can be found in Supplementary Table 1. This score, hereafter referred to as the complementarity score, was used as a proxy for the complementarity between the ${5}^{{\prime} }$ ss and U1 snRNA. For simplicity, we assumed that each potential H-bond contributes equally to the stability of the U1-${5}^{{\prime} }$ ss interaction, acknowledging that individual H-bonds can differ in strength. This approach provides a tractable estimate of sequence complementarity without accounting for such variations. Analyses highlighted a significant difference between the distributions of complementarity scores for AGA exons amenable to induction by NVS-SM1 compared to non-amenable AGA exons (*p* < 0.0001, Fig. 2C). We observed the same difference in analogous analyses of induced and uninduced GA exons upon Risdiplam treatment (Supplementary Fig. 8A). These findings suggested that the complementarity between the ${5}^{{\prime} }$ ss of target exons and U1 snRNA contributes quantitatively to exon induction upon compound treatment and is a crucial determinant of exon responsiveness to the compounds.

**Fig. 3:**
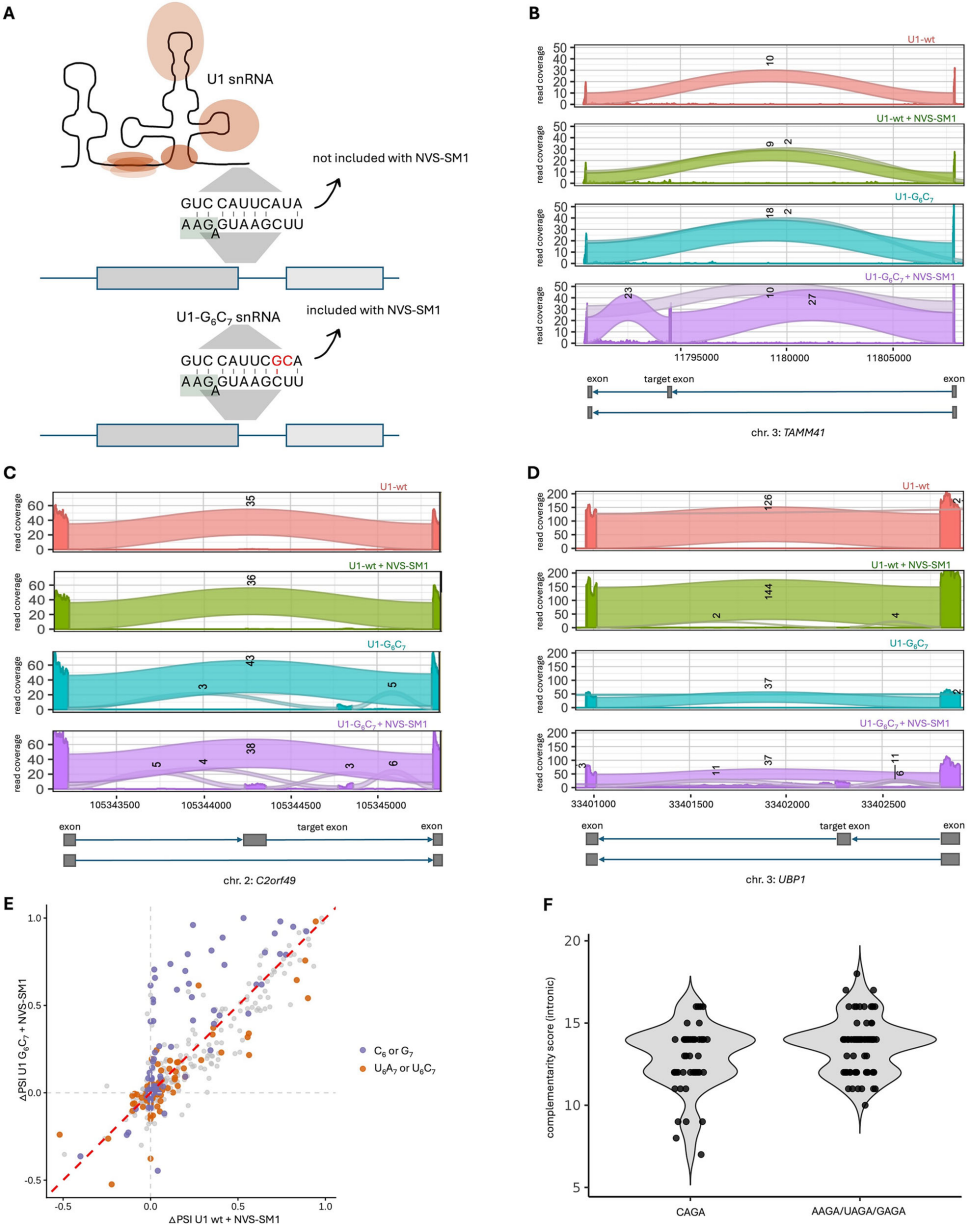
Complementarity between U1 snRNA and the U1 binding site is a critical factor for exon responsiveness. **A** Cartoon showing the complementarity of the exon highlighted in (B) with the canonical and the mutated U1 snRNA. Complementarity score with U1-wt was 19 and the score calculated for U1-G_6_C_7_ was 22. **B** Sashimi plots of RNA-seq results deriving from HeLa cells either transfected with canonical U1 snRNA (U1-wt) or with mutated U1 snRNA (U1-G_6_C_7_), grown in the absence or presence of NVS-SM1, each performed in biological triplicates. Plot highlights a cryptic exon on chromosome 3 within the gene *TAMM41*. Significant inclusion only detected in cells transfected with U1-G_6_C_7_ and treated with 100 nM NVS-SM1 (ΔPSI = 0.8, 10% FDR). **C** Sashimi plots of RNA-seq results deriving from the same experiment as explained in A. Plot highlights an exon on chromosome 2 within the gene *C2orf49*. Significant inclusion only detected in cells transfected with U1-G_6_C_7_ and treated with 100 nM NVS-SM1 (ΔPSI = 0.16, 10% FDR). **D** Sashimi plots of RNA-seq results deriving from the same experiment as explained in A. Plot highlights an exon on chromosome 3 within the gene *UBP1*. Significant inclusion only detected in cells transfected with U1-G_6_C_7_ and treated with 100 nM NVS-SM1 (ΔPSI = 0.52, 10% FDR). **E** Scatter plot comparing exon inclusion changes (ΔPSI) upon compound treatment in cells expressing canonical (U1-wt + NVS-SM1) or mutated U1 snRNAs (U1-G_6_C_7_ + NVS-SM1). The *x*-axis shows values for exons in cells transfected with canonical U1 and treated with 100 nM NVS-SM1, and the *y*-axis shows values for the same exons in cells transfected with U1-G_6_C_7_ under identical treatment conditions. ΔPSI between the two conditions. Exons with higher complementarity to the mutated U1 (carrying a C at position 6 or a G at position 7 of the ${5}^{{\prime} }$ ss) are highlighted in purple, whereas exons with higher complementarity to the canonical U1 are shown in orange. The diagonal line indicates equal ΔPSI between the two conditions. Source data are provided as a Source Data file. **F** Complementarity of intronic base-pairing (positions +1 through +8) of exons included upon NVS-SM1 treatment, grouped according to their -4 position. Left group shows all exons with a C_−4_A_−3_G_−2_A_−1_ sequence (*n* = 541) while the right group shows all other AGA exons (A_−4_A_−3_G_−2_A_−1_, U_−4_A_−3_G_−2_A_−1_ or G_−4_A_−3_G_−2_A_−1_) (*n* = 859). Statistical significance was assessed using a two-sided Wilcoxon rank-sum test (*p* = 0.02). Source data are provided as a Source Data file.

To verify this, we designed an experiment to modify U1 snRNA - pre-mRNA complementarity while leaving the small-molecule binding site unchanged. We ectopically expressed a U1 snRNA, harboring 6A>G and 7U>C mutations to improve pairing with the intronic region of pre-mRNAs (Fig. 3A). We refer to this mutant U1 snRNA as U1-G_6_C_7_, since the changes pair with positions +6 and +7 of the exon’s U1 binding site. Whole transcriptome profiles were sequenced in biological triplicates and analyzed for exons induced by the combination of U1-G_6_C_7_ transfection and NVS-SM1 treatment at 100 nM. Compared to the DMSO-treated cells transfected with U1-G_6_C_7_, 156 AGA exons were found significantly induced upon combining U1-G_6_C_7_ transfection and NVS-SM1 treatment (Supplementary Fig. 1D). Of these, 29 were not detected as significantly induced upon the control combination of U1-wt transfection and NVS-SM1 treatment (FDR = 10%, ΔPSI  > 0.05).

One cryptic exon, located in the *TAMM41* gene and exclusively induced by the combination of U1-G_6_C_7_ expression and NVS-SM1 treatment, demonstrated particularly high inclusion rates, substantiated by clear exon-exon junction reads (ΔPSI=0.8, Fig. 3B). Another cryptic exon in gene *C2orf49* was also found significantly induced upon combination treatment (ΔPSI = 0.16, Fig. 3C). In *UBP1*, a cryptic exon demonstrated only a slight, non-significant increase with U1-wt plus NVS-SM1 (ΔPSI = 0.014), yet a much stronger induction with U1-G_6_C_7_ plus NVS-SM1 (ΔPSI = 0.52, Fig. 3D). Additional examples can be found in Supplementary Fig. 9A–D. Notably, exons that had a stronger complementarity with the mutant U1 snRNA, including the three examples mentioned before, demonstrated higher inclusion in response to NVS-SM1 in cells expressing the respective mutant. Whereas exons better matching U1-wt, responded stronger in cells transfected with the canonical U1 snRNA. These trends were reflected in both when comparing ΔPSIs (Fig. 3E) and absolute PSIs between cells transfected with the different U1 snRNAs in combination to the compound treatments (Supplementary Fig. 10A).

To test whether this observation applies to other splicing modifiers as well, we repeated the experiment using 1 *μ*M of Risdiplam. We compared U1-G_6_C_7_-transfected cells treated with 1 *μ*M of Risdiplam to transfected cells treated with DMSO as a control and found 106 significantly induced GA exons (Supplementary Fig. 1E). Of these, 23 exons were not significantly induced upon the combination of U1-wt transfection and 1 *μ*M Risdiplam. As previously observed for NVS-SM1, this set of exons demonstrated a shift to higher complementarity scores with U1-G_6_C_7_ compared to canonical U1 snRNA (Supplementary Fig. 8B–F and 10B). Overall, these data show that baseline complementarity between U1 snRNA and the target exons contributes to compound-mediated exon induction.

### Role of the -4 exon end position in compound-mediated splicing

In an attempt to unify the A_−1_ bulge formation model^25,29^ and our observations on complementarity requirements, we tested how the -4 nucleotide at the exon’s ${3}^{{\prime} }$ end affects NVS-SM1 and Risdiplam activity. As illustrated in Fig. 2B, a cytosine at position -4 could pair with a guanine in position 11 of the U1 snRNA, assuming an A-1 bulge forms in the pre-mRNA, and thus restoring the canonical ${5}^{{\prime} }$ ss initiation sequence CAG. To test this model in a cellular context, we analyzed the 125 AGA exons induced by NVS-SM1 in SH-SY5Y cells and grouped them according to their -4 position, resulting in 50 exons with a ${5}^{{\prime} }$ -CAGA-${3}^{{\prime} }$ ending and 75 exons with either UAGA, GAGA or AAGA ends. Strikingly, when focusing on the complementarity of the intronic base-pairing (positions +1 through +8) to a canonical U1 snRNA, we found that low-complementarity sites favor a cytosine at position -4 (*p* = 0.02, Fig. 3F). This suggests that base-pairing at position -4 can help compensate for weak U1 binding in the intron.

**Fig. 4:**
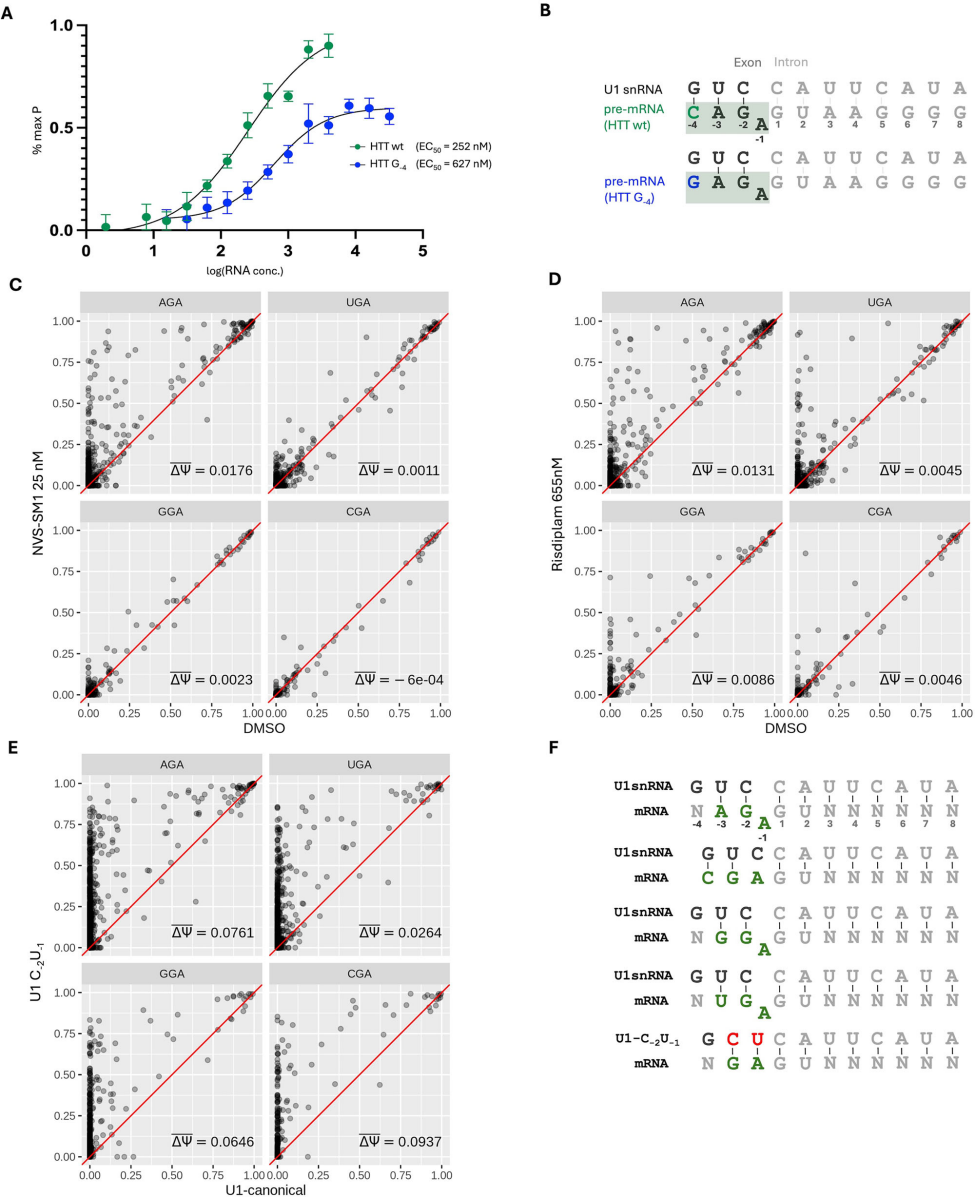
Bulge formation and sequence features of exons induced by NVS-SM1 and Risdiplam. **A** Fluorescence polarization (FP) binding curves of TAMRA-labeled U1 snRNA incubated with serial dilutions of the U1 binding site of the *HTT* pseudoexon, either the wild-type sequence (wt), or a mutated version (HTT G_−4_). Data are presented as mean  ± s.d. of *n* = 8 technical replicates from a single experiment. Curves were fitted using nonlinear regression with a sigmoidal dose-response model (EC_50_ = 252 nM for wt and 627 nM for HTT G_−4_). Source data are provided as a Source Data file. **B** Constructs used for the FP-assay. **C**–**E** Scatter plots showing PSI values of GA-ending exons, grouped according to their -3 position (AGA, UGA, GGA, or CGA). The red diagonal line indicates equal PSI values between the compared conditions and $\overline{\Delta \Psi }$ the mean change in inclusion. Each point represents an individual exon. Source data are provided as a Source Data file. **C** PSI values in cells treated with 25 nM NVS-SM1 or DMSO. **D** PSI values in cells treated with 655 nM Risdiplam or DMSO. **E** PSI values in cells transfected with either U1-C_−2_U_−1_ or canonical U1 snRNA. **F** Schematic of potential bulge formation in RNA duplexes between U1 snRNA and the exon-intron junction of GA-ending exons. U1-C_−2_U_−1_ shows sequence of the mutated U1 snRNA, modified to directly induce GA-ending exons in the absence of a compound.

To experimentally test the influence of the -4 position on the formation of an RNA duplex with a A_−1_ bulge, we employed a Fluorescence Polarization (FP) assay based on a TAMRA-labeled 12 nucleotide RNA representing the U1 snRNA sequence. With increasing concentrations of *HTT* pseudoexon ${5}^{{\prime} }$ ss, we observed an increase in fluorescence polarization, indicating binding of the labeled U1 sequence (EC_50_ = 252 nM, Fig. 4A/B). Repeating the experiment with a target RNA harboring a C>G mutation at the -4 position (-4C>G, Fig. 4B), we observed a significant decrease in FP signal compared to the wt RNA (EC_50_ = 627 nM, Fig. 4A). This finding corroborates the influence of the nucleotide at position -4 on the overall base pairing between U1 snRNA and ${5}^{{\prime} }$ ss, which can compensate for weak intronic U1 snRNA basepairing.

### Primary sequence features of GA exons induced by NVS-SM1 and Risdiplam

Considering the influence of the -4 position on the pairing between U1 snRNA and non-canonical GA exons, we analyzed the primary sequence features of exons induced by NVS-SM1. In line with the established GA-specificities of these compounds, we observed an enrichment for GA exon ends (positions −2 and −1) in the ${5}^{{\prime} }$ ss motifs of NVS-SM1 and Risdiplam responsive exons in SH-SY5Y cells. In NVS-SM1 treated cells, the A_−3_G_−2_A_−1_ motif was enriched (Fig. 1C). Analyzing the nucleotide distribution at position -2 of all induced A-ending exons, we found a near-exclusive occurrence of guanine (91%, i.e., 146 out of 160 upon NVS-SM1 treatment and 94%, i.e., 151 out of 161 upon Risdiplam treatment) (Supplementary Fig. 4A). Consistent with the observed motif, a robust enrichment for adenine in response to NVS-SM1 could be detected at the -3 position of induced GA-ending exons (86%, i.e., 125 out of 146). This motif preference correlated with exon responsiveness upon compound treatment, reflected by overall PSI values. AGA-ending exons are most efficiently induced by NVS-SM1, while CGA-ending exons, intriguingly, did not show any average inclusion changes (${\overline{\Delta \Psi }}_{{{\rm{CGA}}}}=-6\times 1{0}^{-4}$) (Fig. 4C). This pattern is consistent with the differential exon inclusion analysis, which revealed that NVS-SM1 induces several GGA and UGA exons (14 and 7, respectively) but no CGA exons (Supplementary Fig. 4B).

To explore whether CGA exons can in principle be induced, we transfected HeLa cells with a modified U1 snRNA harboring -2U>C and -1C>U mutations, designed to stabilize all GA-ending exons (U1-C_−2_U_−1_; Fig. 4F). Transcriptome sequencing of transfected cells identified exons specifically induced by U1-C_−2_U_−1_ compared to those induced by canonical U1 snRNA (Supplementary Fig. 1F). Under these conditions, CGA exons show increased inclusion comparable to the effect on other GA-ending exons, with the highest mean change among them (Fig. 4E, ${\overline{\Delta \Psi }}_{{{\rm{CGA}}}}=0.0937$). The differential analysis revealed inclusion of 96 CGA exons (9% of all induced GA-ending exons; Supplementary Fig. 4E). In contrast, when transfecting cells with the canonical U1 snRNA and treating cells with NVS-SM1, inclusion of CGA-ending exons is almost absent, with only one detected exon (less than 1% of induced GA-ending exons; Supplementary Fig. 4E). Similar to Branaplam, also Risdiplam disfavored CGA-ending exons (Fig. 4D and Supplementary Fig. 4B). These findings confirm that CGA exons are generally amenable to splicing modulation when U1 binding is stabilized but are refractory to small-molecule-mediated activation under native conditions. A likely explanation is that the C_−3_G_−2_A_−1_ sequence prevents formation of an A_−1_ bulge due to the energetically favorable G-C pairing at position -3 (Fig. 4F). Indeed, according to RNAcofold predictions, the probability that the G_−2_ of the CGA exons basepairs with the C of the U1 snRNA (an interaction required to stabilize a A_−1_ bulge) was of 0 (Supplementary Fig. 5B). These observations suggests that the bulge stabilization at position -1 is not just the result of compound binding, but actually a prerequisite for it. To further test this model, we transfected cells with an U1 snRNA variant (U1-C_−2_G_−1_) that disfavors bulge formation with AGA exons. RNAcofold predictions confirmed that the U1-C_−2_G_−1_ mutant retained the same binding free energy as the wild-type U1 for interaction with AGA exons while exhibiting a zero probability of forming the base pairs required to stabilize a bulge formation at position -1 (Supplementary Fig. 6). Expression of U1-C_−2_G_−1_ in cells moderately reduced the splicing response to NVS-SM1, further supporting that compound activity in the spliceosome depends on the ability to form an A_−1_ bulge (for AGA exons with ΔPSI  > 0.05 in U1wt, U1-C_−2_G_−1_ showed a mean ΔPSI reduction of 0.04) (Supplementary Fig. 4F).

**Fig. 5:**
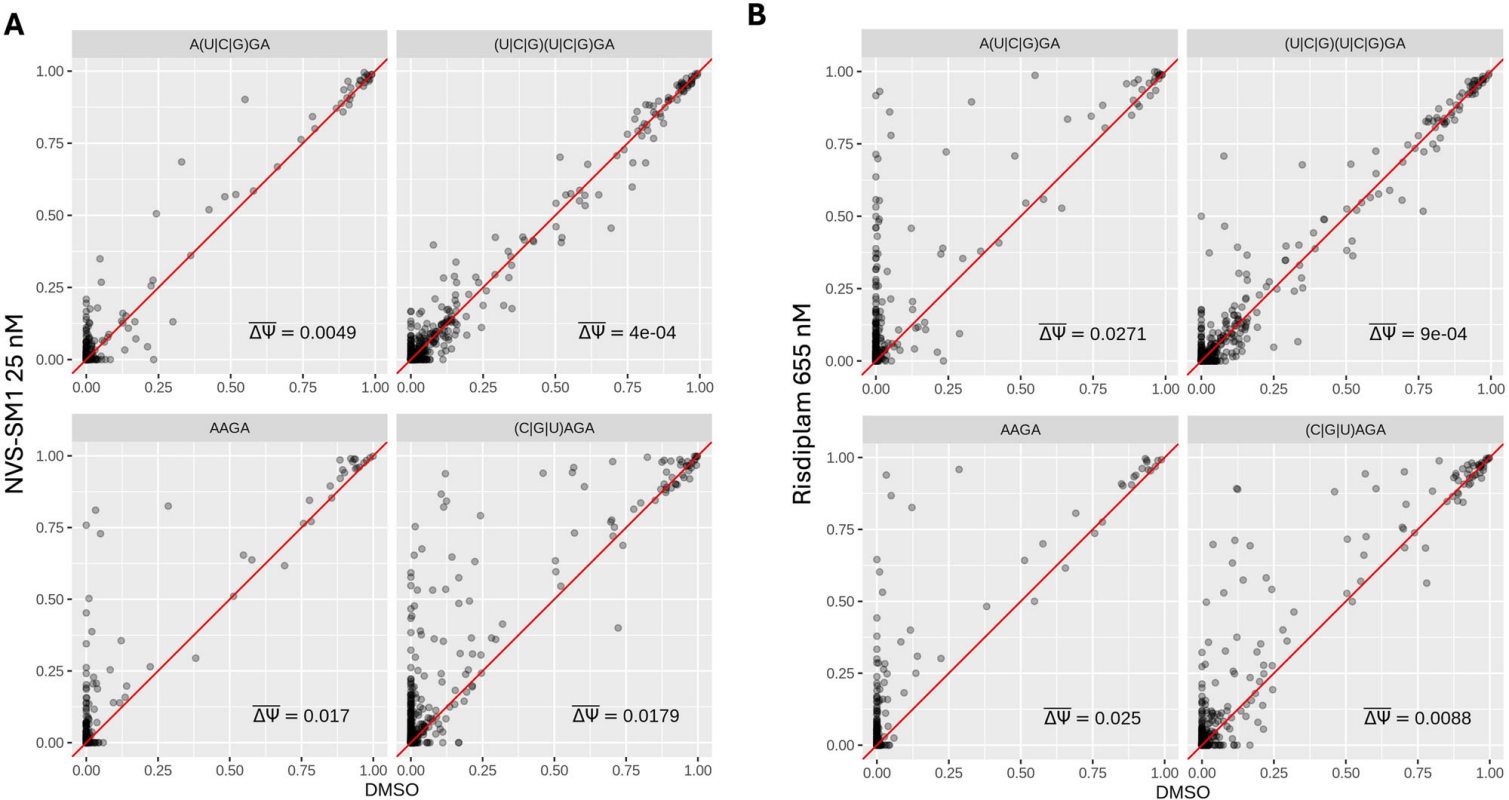
Necessity of an A at -3 or -4 position. Scatter plots showing PSI values of GA-ending exons in cells treated with a compound or DMSO. Exons are grouped based on the presence of adenine at the -3 and -4 positions: A at both positions (AAGA), A only at -3 ((C∣G∣U)AGA), A only at -4 (A(C∣G∣U)GA), or A absent at both positions ((C∣G∣U)(C∣G∣U)GA). The red diagonal line indicates equal PSI values between the compared conditions. The mean PSI change $\overline{\Delta \Psi }$ is shown for each panel. Source data are provided as a Source Data file. **A** Cells treated with 25 nM NVS-SM1. **B** Cells treated with 655 nM Risdiplam.

As mentioned, with NVS-SM1 treatment, AGA exons showed the strongest increase in inclusion compared to DMSO controls. Intriguingly, for GA exons lacking an adenine at position -3, we noticed a global compound response only when an adenine was present at position -4 (A(C∣G∣U)GA) (Fig. 5A). In contrast, GA-ending exons without adenine at either positions -4 or -3 ((C∣G∣U)(C∣G∣U)GA) display almost no change in inclusion ($\overline{\Delta \Psi }=4\times 1{0}^{-4}$, Fig. 5A). Analyses of sequence composition of statistically significantly included exons supported these observations: while induced AGA exons exhibit an even nucleotide distribution at position -4 (Supplementary Fig. 4C), significantly induced GGA and UGA exons almost exclusively feature an adenine at position -4 (Supplementary Fig. 4D). Risdiplam showed the same pattern, where only GA-ending exons containing a second adenine at either position -3 or -4 also showed a response to the compound ($\overline{\Delta \Psi }=9\times 1{0}^{-4}$, Fig. 5B and Supplementary Fig. 4C). The strong preference for AGA-, AGGA-, and AUGA-ending exons thus reflects a broader requirement for a secondary adenine at positions -3 or -4. Furthermore, RNAcofold predictions showed that an adenine in position -4 has a zero predicted probability of interacting with matching guanine of the U1 snRNA (Supplementary Fig. 7), indicating that it is unlikely to contribute to stabilization of the U1 - ${5}^{{\prime} }$ ss duplex. Therefore, the presence of a second adenine at position -4 of exons is more likely to favor compound binding rather than U1 engagement.

### NVS-SM1 interaction with A-1 bulge in RNA duplexes

**Fig. 6:**
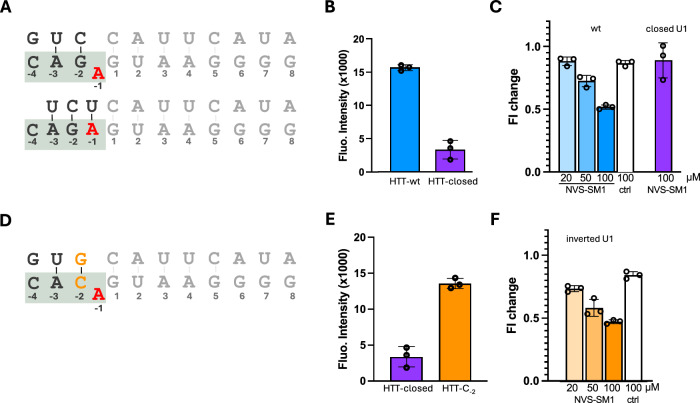
Biochemical Assays to investigate bulge formation at U1 binding site of the *HTT* pseudoexon. **A** RNA constructs used for the 2-AP assay shown in (**B**) and (**C**). RNA constructs carrying either wild-type sequence of the RNA complex formed when *HTT* pseudoexon is recognized (wt, top) or a mutated version, forming a closed duplex (bottom, closed U1). Position of the 2-AP mutation highlighted in red. **B** Fluorescence Intensity (FI) measured in the 2-Aminopurine (2-AP) assay with wt or closed U1 RNA construct (sequences shown in (A)). Data are presented as mean  ± s.d. of *n* = 3 technical replicates. Individual data points representing technical replicates are overlaid on bar plots. Source data are provided as a Source Data file. **C** Changes in FI upon titration of NVS-SM1 or inactive compound (ctrl, shown as neg.ctrl in Supplementary Fig. 1G) to RNA duplexes, relative to the DMSO condition. Data are presented as mean  ± s.d. of *n* = 3 technical replicates. Individual data points representing technical replicates are overlaid on bar plots. Source data are provided as a Source Data file. **D** RNA constructs used for the 2-AP assay shown in (**E**) and (**F**). RNA construct carries a mutated version of the RNA complex formed when *HTT* pseudoexon is recognized, changing primary sequence while maintaining possibility of bulge formation. 2-AP mutation highlighted in red. **E** Fluorescence Intensity (FI) measured in the 2-Aminopurine (2-AP) assay with the closed U1 or inverted U1 RNA construct (sequences shown in (**A**) and (**D**)). Data are presented as mean  ± s.d. of *n* = 3 technical replicates. Individual data points representing technical replicates are overlaid on bar plots. Source data are provided as a Source Data file. **F** Changes in FI upon titration of NVS-SM1 or inactive compound (ctrl, shown as neg.ctrl in Supplementary Fig. 1G) to RNA duplexes, relative to the DMSO condition. Data are presented as mean  ± s.d. of *n* = 3 technical replicates. Individual data points representing technical replicates are overlaid on bar plots. Source data are provided as a Source Data file.

To understand the dynamics of A_−1_ bulge structure stabilization, we designed a 13 nucleotide RNA probe containing the ${5}^{{\prime} }$ ss sequence of the *HTT* pseudoexon (positions -4 to +9), in which we replaced the adenine at position -1 with the fluorescent analogue 2-aminopurine (2-AP). The fluorescence of 2-AP depends on its microenvironment and, while un-impacted by events such as hydrogen-bond formation, is being quenched by base-stacking and clashes with neighboring nucleobases^30^. Therefore, 2-AP can act as a sensitive reporter to monitor changes to the secondary or tertiary structure of RNA, when incorporated into a nucleotide strand^31^. Moreover, those characteristics can be applied to detect and monitor ligand-engagement to RNA^19,32^. Thus, the 2-AP probe enabled monitoring both of A_−1_ bulge secondary structure formations. We incubated the 2-AP probe with wild-type U1 snRNA or modified U1 snRNA oligonucleotides, respectively. The modified U1 oligo, harboring the mutations -3G>U, -2U>C and -1C>U, reverse complements the 2-AP probe and thus forms an RNA duplex without any mis-match induced secondary structures. Hence, we termed this modified U1 snRNA oligo “closed U1” (Fig. 6A).

As expected, adding the closed U1 construct caused strong quenching of 2-AP fluorescence, indicating base stacking in the RNA duplex (Fig. 6B). Conversely, when adding the canonical U1 RNA oligo, the 2-AP fluorescence was significantly higher compared to the closed construct, indicating that the 2-AP base was not stacked in the RNA duplex, thus confirming the presence of a bulge structure (Fig. 6A). Next, we monitored the 2-AP fluorescence intensity (FI) changes in the presence of canonical U1 RNA oligo, with increasing concentrations of either NVS-SM1 or a structurally related, splicing-inactive compound (ctrl; Supplementary Fig. 1G). While the inactive compound did not cause any fluorescence decrease, NVS-SM1 caused a concentration-dependent quenching of the 2-AP FI, confirming its interaction with the A_−1_ bulge in the RNA duplex (Fig. 6C).

### Primary and secondary RNA structures define the splicing modulator target exon space

To better understand how RNA structure and sequence affect compound binding, we adapted the 2-AP assay to test whether NVS-SM1 could bind the same secondary structures in the context of different primary sequences. Therefore, we modified the HTT ${5}^{{\prime} }$ ss 2-AP probe with a -2G>C mutation to mimic an ACA-ending exon. To match this oligo probe, we designed a 12 base oligonucleotide with the U1 snRNA sequence harboring a -2C>G mutation, called “inverted U1” (Fig. 6D). The combination of both the modified HTT ${5}^{{\prime} }$ ss probe and the inverted U1 oligo allows for and favors the formation of a bulged-out 2-AP reporter nucleotide (Fig. 6C/F). The baseline fluorescence intensity of the HTT-C_−2_ RNA complex was found similar to the FI observed for the HTT-wt complex (Fig. 6F), suggesting a resembling formation of a bulged-out adenine reporter nucleotide at position -1 of the RNA duplex. Subjecting this reporter complex to increasing concentrations of NVS-SM1, we observed a dose-dependent quenching of the 2-AP probe, this time embedded into a different sequence context (Fig. 6F). These data demonstrate that binding of NVS-SM1 to adenine bulge structures occurs independent of the primary RNA sequence. This observation, in line with our genetic perturbation experiments, highlights the effect of the highly conserved U1 snRNA sequence as a major determinant, governing the specificity of small molecule splicing modulators, such as NVS-SM1.

### Contribution of regulatory elements

**Fig. 7:**
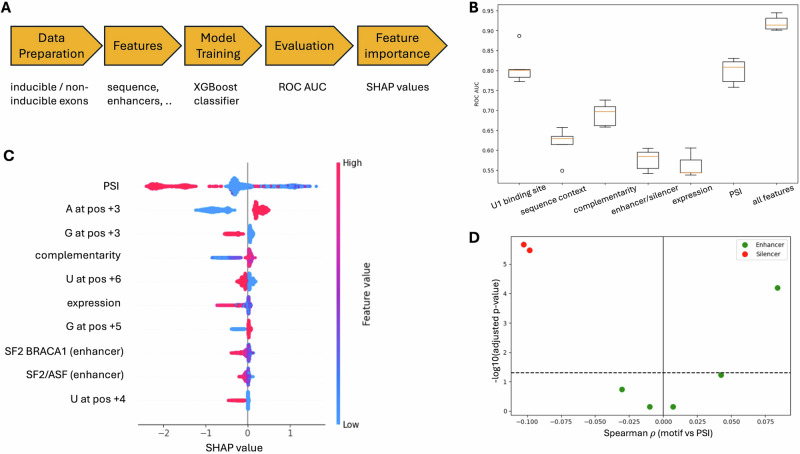
Machine-learning model and contributions of different features to prediction. **A** Schematic overview of the machine-learning workflow. The model integrates sequence features, enhancer and silencer scores, and expression information to distinguish inducible AGA exons from non-inducible AGA exons. An XGBoost classifier was trained and evaluated using cross-validated ROC AUC, followed by SHAP-based feature interpretability. **B** Comparison of model performance across different feature sets using 5-fold cross-validated ROC AUC. Each boxplot represents the distribution of AUC values across folds for one feature set (*n* = 5 cross-validation folds). Box plots indicate the median (center line), interquartile range (box), and whiskers extending to 1.5 × the interquartile range. Source data are provided as a Source Data file. **C** SHAP summary plot displaying the top features contributing to model predictions. Each point represents one splice site. The horizontal position indicates the direction and magnitude of the contribution to the prediction, and the color represents the original feature value. Source data are provided as a Source Data file. **D** Volcano plot showing the correlation between enhancer and silencer motif abundance and basal PSI in DMSO samples. Each point represents a motif, colored by category. The *x*-axis displays the Spearman correlation coefficient (*ρ*), and the *y*-axis shows $-{\log }_{10}$ adjusted *p* values. Spearman correlations were calculated and statistical significance was assessed using a two-sided test with Benjamini-Hochberg correction for multiple comparisons. Horizontal and vertical reference lines indicate the significance threshold (adjusted *p* = 0.05) and zero correlation, respectively. Source data are provided as a Source Data file.

We further characterized the contribution of regulatory elements, beyond the U1 snRNA binding sites, to compound activity. Analyses of the abundance of predicted exonic splicing enhancer (ESE) and exonic splicing silencer (ESS) motifs showed no significant differences between induced AGA exons and non-induced AGA exons. To integrate broader regulatory information, we trained a gradient-boosted decision tree model (XGBoost, Fig. 7A, B) to classify AGA exons between inducible and non-inducible using sequence context and motif predictions. Overall, Shapley additive explanations values (SHAP) of the model confirmed our previous observations on the contribution of the different U1 snRNA positions to compound mediated exon induction (Fig. 7C).

Moreover, a model based solely on enhancer and silencer predictions performed only marginally above random (Fig. 7B), confirming that these predictions alone provide a limited explanatory power. In contrast, a model trained on the surrounding sequence context, while explicitly excluding the U1 snRNA binding site, achieved a ROC AUC above 0.6 (Fig. 7B), demonstrating that additional regulatory information embedded in the flanking sequence contributes to compound responsiveness. Notably, a model restricted to the U1 binding site itself (positions −4 to +8) performed even better, reaching a ROC AUC of 0.8 (Fig. 7B). This underscores that this region remains the dominant determinant of responsiveness, while the broader sequence context provides secondary but meaningful regulatory cues. The SHAP analysis identified baseline inclusion (PSI in DMSO) as one of the most influential features. Specifically, AGA exons with a high baseline PSIs were not further induced by compound treatment. Interestingly, we found that baseline PSI was significantly correlated with the number of ESE and anticorrelated with the number of ESS (Fig. 7D).

Overall, the interpretation of this model aligns with our previous analyses presented in this study, which highlighted the several key characteristics, such as complementarity with the U1 snRNA. Further, these results suggest that compound responsiveness is also shaped by subtle sequence embedded features and integrated regulatory effects.

## Discussion

This study provides insights into the mechanisms underlying the specificity of small molecule splicing modifiers, particularly focusing on NVS-SM1 (Branaplam) and its interaction with the splicing machinery. Although these compounds target exons with specific exon-end, not all of these exons are equally responsive. This suggests that additional determinants governing compound specificity are required to render an exon responsive to small molecule splicing modifiers. By integrating transcriptomic profiling, biochemical assays, U1 perturbation experiments, regulatory motif annotation, and machine-learning-based feature interpretation, we identify multiple layers of regulation that together determine whether an exon can be modulated by small molecules.

First, we highlighted that the landscape of inducible exons is shaped significantly by cell type specific expression, alongside with primary sequence. Furthermore, we analyzed the contributions of the downstream intronic nucleotides within the U1 snRNA binding site of the target exon. Their functional importance has remained largely unexplored in the context of small-molecule splicing modulation, particularly across different exon architectures. It has not only remained unclear whether these positions consistently influence compound responsiveness across different exons, but also how they contribute mechanistically to modulator activity. Our investigation into their complementarity between the ${5}^{{\prime} }$ ss and the U1 snRNA revealed a crucial role that the U1 snRNA plays in mediating compound-mediated exon inclusion. Exons with higher complementarity proved more likely to be targetable by splicing modifiers, whereas those with lower complementarity tend to be skipped, regardless of the intervention. Our experiments with mutated U1 snRNA showed that changing U1-${5}^{{\prime} }$ ss pairing can reprogram compound specificity. This, in turn, highlights the strong dependence of the observed compound specificity on the highly conserved U1 snRNA sequence and explains why the actual specificity of these compounds tends to exceed theoretical expectations.

To gain a deeper understanding of what governs the apparent primary sequence specificity of NVS-SM1 and Risdiplam, we took a closer look at potential bulge formation at the -1 position of the exon end. In line with previously reported biophysical data^27^, our cellular and biochemical data corroborates the necessity of a bulge formation at the -1 position of the exon end. In other biological contexts, covariation helps to identify RNA structural specificity; however, in this case, covariation does not occur due to the high conservation of the U1 snRNA sequence. Thus, we used mutated U1 snRNA in our assays to test if we could shift the sequence specificity of the compounds. Notably, we were able to induce compound binding to a previously non-amenable CA-ending exon when the U1 snRNA was mutated to maintain a bulged-out adenine at the −1 position of the pre-mRNA ${5}^{{\prime} }$ ss. Also, our transcriptomic analyses showed that exons lacking the ability to form a bulge at the -1 position (CGA-ending exons), even if they fulfill the N_−3_G_−2_A_−1_ motif, are refractory to compound modulation. Furthermore, expressing a U1 snRNA variant that disfavors this bulge formation with AGA exons reduces the effect of NVS-SM1 on AGA exons. These findings further emphasizes that the formation of the required U1-${5}^{{\prime} }$ ss RNA duplex secondary structure is not a merely the result of compound binding, but rather a prerequisite for compound binding.

Consistent with observations by Ishigami et al.^21^, we also identified that the -4 position as a critical determinant of compound responsiveness. Our results corroborate that this position substantially influences drug activity and provide an explanation for why many N_−3_G_−2_A_−1_-ending exons fail to be induced. For AGA exons, the presence of a C in the -4 position compensates for low U1 snRNA intronic complementarity. Furthermore, our findings lead us to propose that the AGA or ANGA sequence specificity arises from two key factors. Firstly, it is essential to maintain a specific secondary structure that allows for bulge formation, resulting in the requirement of a G at the -2 position. Secondly, two adenines appear to be essential, one at position −1 and one at either position −3 or −4. Since an adenine at position -4 is unlikely to contribute to U1-${5}^{{\prime} }$ ss duplex stabilization, these adenines are likely forming interactions with the compounds, thereby contributing to the observed primary sequence specificity. Together, these results define primary sequence codependencies that shape bulge formation and the surrounding U1-${5}^{{\prime} }$ ss duplex architecture as the primary determinants of NVS-SM1 and risdiplam responsiveness.

However, the presence of unresponsive exons that do match our identified characteristics of inducibility suggests additional features and regulatory elements. Previous publications have highlighted the importance of such elements. For example, the purine-rich element in SMN2 exon 7^33^, have been shown to shape compound binding. Further work on extended exonic sequence similarly shows that local context can influence drug activity^34^. Although our genome-wide analyses did not reveal any motif beyond the U1 binding site or splicing enhancers that were broadly present in compound-induced exons, we cannot exclude that individual regulatory elements may still modulate responsiveness in specific exons. Indeed, in our machine learning model, the feature importance analysis highlighted a contribution of local sequence context to compound modulation. However, baseline AGA exon inclusion in the absense of a compound was correlated with the number of ESE and anticorrelated with the number of ESS, suggesting that these regulatory elements may compensate for AGA weak splice sites under normal physiological conditions.

Overall, our findings shed light on the intricate interplay between primary sequence and secondary structure requirements in governing the overall observed specificity of small molecule splicing modifiers in cells. Understanding this is essential to decipher the rules governing compound specificity, improve predictive models for target exons, and potentially tune the activity of splicing modulators in a controlled manner. On one hand, the specificity of a U1 snRNA can be influenced by the addition of a splicing-modulating compound. Genetic manipulation of the U1 snRNA, on the other hand, can change the specificity of a splicing modulating compound. Since our study focuses on NVS-SM1 and Risdiplam, future studies will be needed to similarly elucidate the target landscape of other splicing modulators^35^. The insights presented by our study may aid future development of small molecules targeting RNA splicing as well as RNA secondary structures. Moreover, these findings will help to refine prediction algorithms, paving the way for identification of additional targets and rational design of splicing-modifying compounds.

## Methods

### Cell culture

All cells were obtained from commercial sources, maintained in-house and periodically tested for mycoplasma contamination. SH-SY5Y neuroblastoma cells were cultured using Advanced MEM media supplemented with 10% heat-inactivated fetal bovine serum (HI FBS) and 1X Glutamine (5 mL per 500 mL of media). Upon reaching confluency, the cells were sub-cultured and plated into 12-well plates at a density of 0.6 × 10^6^ cells/mL, with 1 mL per well. On the second day post-plating, the cells were treated with compounds at different concentrations, DMSO was used as a control. Cells were harvested 24 h post-treatment. For iNGN2 cells, day 3 iNGN2 neurons were thawed and plated onto six-well plates, pre-coated with poly-D-lysine (PDL) and a mixture of 1X Matrigel and laminin (1:300, Sigma-Aldrich, L2020-1MG). The coating procedure involved incubating the plates at 37 °C for at least 1 to 1.5 h prior to cell plating. Neurons were maintained by providing half of the usual volume of culture medium every other day until day 10. On day 10, they were treated with compounds at different concentrations. DMSO was used as a control. Cells were harvested 24 h post-treatment. HeLa293 cells were maintained in DMEM with 10% fetal bovine serum (FBS) and 1% GlutaMax at 37 °C with 5% CO_2_. They were plated on a 24-well plate (80.000 cells/well) and treated with potential compounds or DMSO as control the next day. Cells were harvested 24 h post-treatment.

### Expression of U1 snRNA

For the ectopical expression of U1 snRNA, we used a plasmid either carrying the sequence of the canonical U1 snRNA or a mutated U1 snRNA version (e.g., U1-G_6_C_7_). Plasmid constructs encoding U1 snRNA variants were generated by GeneArt gene synthesis (Thermo Fisher Scientific) and delivered in standard cloning vectors lacking mammalian promoters. The sequences were based on the human U1 snRNA genomic sequence described previously^36^. All constructs were verified by sequencing prior to use. HeLa cells were plated on a 24-well plate (80.000 cells/well) and transfected the following day with 0.3 *μ*g DNA and 0.9 ml Lipofectamine^TM^ 2000 (Thermo Fisher Scientific), according to the supplier’s protocol. After 24 h, medium was changed and potential compounds added or DMSO as a control. After 24 h incubation, RNA purification was performed using the *Quick*-RNA Miniprep Kit (Zymo Research).

### NMD inhibition

We inhibited major RNA decay pathways in SH-SY5Y cells by depleting XRN1 and EXOSC4. The culture medium was aspirated and replaced with siRNA delivery medium containing Accell siRNA targeting XRN1 and EXOSC4 or with a non-targeting control (NTC). The following reagents were used: Accell Human XRN1 siRNA SMARTpool (E-013754-00-0050), Accell Human EXOSC4 siRNA SMARTpool (E-013760-00-0050), and Accell Non-targeting Pool (D-001910-10-50) (all from Horizon Discovery Biosciences). Cells were incubated for 72 h to achieve gene knockdown. Following this incubation, either DMSO or the splicing compound was added to the cells for an additional 24 h. Knockdown efficiency was verified by qRT-PCR.

### RNA sample preparation and sequencing

RNA samples were collected from SH-SY5Y and iNGN2 cells treated with DMSO or compounds using the RNeasy Plus 96 Kit (Qiagen) according to the manufacturer’s instructions. RNA purification from HeLa cells purification was performed using the *Quick*-RNA Miniprep Kit (Zymo Research). All RNA-seq experiments were performed in biological triplicates. RNA quality was assessed using the TapeStation 4200, with all samples exhibiting an RNA integrity number (RIN) greater than 9. Next-generation sequencing libraries were prepared with the TruSeq Stranded mRNA Sample Preparation Kit (Illumina) from 200 ng of input RNA, following the manufacturer’s protocol. The libraries were then pooled and loaded onto a NovaSeq 6000 (Illumina) for paired-end 51 bp sequencing, yielding an average of 30-40 million reads per sample. Quality control analyses confirmed high reproducibility among biological replicates and clear separation between treatment groups. Principal component analysis (PCA) of exon inclusion levels demonstrated clustering by treatment condition (Supplementary Fig. 11 and 12), and pairwise sample-sample correlations exceeded 0.95 across replicates (Supplementary Figs. 13, 14, 15, 16, 17 and 18), confirming data consistency prior to downstream analyses.

### Detection of novel exons and compound-mediated splicing

RNA-seq data were mapped to the hg38 human reference genome using STAR. To identify unannotated exons in the RNA-seq data, we used the split alignments reported by STAR. For a given RNA-seq sample, we mapped introns based on reads whose mapping to the reference genome was split between non-consecutive nucleotides (split reads). For two consecutive annotated exons, these reads would partially map to one exon and partially to the second exon, thus leaving an unmapped region in between that corresponds to the intronic coordinate. As the mapping of all intronic regions of a gene defines the coordinates of the non-terminal exons of a gene, these regions were considered potential novel exons. We considered introns for a given sample when they were supported by at least 2 reads and filtered for novel exons that were supported by at least one third of the samples in each experiment. These potential novel exons, if unannotated, were included to the reference transcript annotation for downstream analyses. For each exon in each sample, we counted the number of reads whose mapping supported the inclusion of the exon and the number of reads whose mapping supported the exclusion of the exon. We estimated percent spliced in values as described in ref. ^37^ and used the Bioconductor package DEXSeq to test for differences between conditions as described in ref. ^38^.

### FP binding assay

In the FP binding experiments, 20 nmol/L TAMRA-labeled U1 snRNA target oligonucleotides were incubated with increasing concentrations of probe oligonucleotides carrying the sequence of the U1 binding site of the pseudoexon in the *HTT* gene that is being included in response to NVS-SM1. 50 mM Tris-HCl (pH 7.4), 5 mM MgCl_2_, 75 mM KCl and 0.05% Tween were used as buffer. The samples were thoroughly mixed, incubated for 5 min at 95 °C to unfold possible RNA structures and slowly cooled down to room temperature (RT). Splicing modifiers were added and incubated for another 1 h at RT. In white 384-well microplates with 30 *μ*L per well using a PHERAstar, the parallel emission intensity (S) and perpendicular emission intensity (P) were measured. FP values were calculated using the following equation, as described previously^39,40^: *FP*(*mP*) = 1000*(*S* − G*P)/(*S* + G*P) All samples were measured in technical triplicates. The FP values were plotted against the concentration of the probe RNA oligonucleotide in each experiment. Using GraphPad Prism 9.0, a sigmoidal dose-response curve was added to determine EC_50_ values. Sequences of all used oligonucleotides are provided in Supplementary Table 2.

### 2-AP assay

Oligonucleotides carrying the sequence of the exon-binding part within the U1 snRNA and those with sequences corresponding to the U1 binding site that contain a 2-AP substitution at the -1 position, were mixed in a 1 × Assay Buffer (0.185 M NaCl, 8 mM NaH_2_PO_4_, pH 6.3). In the absence or presence of potentially binding compounds, they were heated to 70 °C for 3 min and slowly cooled down to allow folding. In the absence of a compound DMSO is used as control. Fluorescence excitation and emission wavelengths (310 nm and 390 nm, respectively) were measured in a 384-well plate at RT with a Tecan plate reader (gain, 155; integration time, 20 *μ*s). All assays were carried out in technical triplicates. Sequences of all used oligonucleotides re provided in Supplementary Table 2.

### RNAcofold predictions

Intermolecular base-pairing probabilities were calculated using RNAcofold (ViennaRNA Package^41^). The resulting AB dot-plot files were parsed to extract intermolecular pairing probabilities, which were plotted as heatmaps. Minimum free-energy values were taken directly from the RNAcofold output.

### Analysis of exonic splicing enhancers and silencers

ESEs were identified using PSSMs for the SR-protein motifs SF2/ASF, SF2/ASF (IgM/BRCA1 variant), SC35, SRp40, and SRp55^42,43^. For each exon the last 50nt were scanned, and scored by summing nucleotide-specific PSSM weights. For each motif, we recorded the number of scoring above the motif’s canonical threshold, as well as the maximum and mean PSSM score across the exon. ESS content was assessed by scanning for hexamer motifs belonging to the published FAS-hex2 (176 hexamers) and FAS-hex3 (103 high-confidence hexamers) datasets^44^. For each exon, we computed the density ESS hits within the last 50nt.

### Machine-learning classification of induced exons

To predict exon inducibility, we trained gradient-boosted decision tree models (XGBoost) using different feature groups. For each feature set, we performed 5-fold stratified cross-validation and computed ROC AUC values. Feature vectors were generated by flattening all list- or tensor-based entries into numerical arrays. Hyperparameter optimization identified a best-performing model with regularization, subsampling, and class-imbalance correction.

### Computational workflow

Computational analyses were performed using custom R scripts to characterize compound-induced splicing changes, followed by Python-based machine-learning analyses to predict exon inducibility and interpret feature importance. Processed RNA-seq-derived datasets were used as input for all analyses with the following workflow: (1) Load processed splicing datasets. (2) Quantify exon inclusion and compute PSI values per exon and sample. (3) Identify compound-induced splicing changes using differential exon usage analysis. (4) Extract exon sequence and splicing features to find patterns driving inducibility. (5) Train decision tree models to predict compound-mediated inclusion. (6) Interpret feature contributions using SHAP. All computational analyses were performed using code made publicly available in the repository (Code Availability Section), which contains the scripts and notebooks used to analyze the data. The repository provides access to the analysis code used to generate the reported results. Processed data underlying the figures and analyses are included in the corresponding Zenodo entry, which the code uses as input for the provided analysis workflows. The computational environment and dependencies required to reproduce the analyses are documented within the repository. The repository is distributed under the MIT License.

### Reporting summary

Further information on research design is available in the Nature Portfolio Reporting Summary linked to this article.

## Supplementary information

Supplementary Information

Reporting Summary

Transparent Peer Review file

## Source data

Source Data

## Data Availability

The RNA-seq data generated in this study have been deposited in the NCBI Sequence Read Archive (SRA) under BioProject accession code PRJNA1293306 (raw sequencing data and sample metadata). Processed data generated in this study are available in Zenodo under 10.5281/zenodo.18416224^45^ (exon inclusion (PSI) values per sample, processed count tables, and feature tables used in the machine learning analyses). These datasets provide the minimum data necessary to interpret, verify, and reproduce the findings of this study. Source data are provided with this paper.

## Materials availability

Plasmids and constructs generated in this study are available from the corresponding authors, subject to standard material transfer agreements and institutional policies.

## Code availability

All scripts used for data processing, analysis, and figure generation are publicly available at GitHub (https://github.com/Novartis/CompExonSpace)^46^. The repository includes documentation and instructions for reproducing the analyses.
