# Supplementary material for: Specificity and exon target space of splicing modifying compounds
Source: Nat Commun. 2026 Jul 4;17:8391. doi: 10.1038/s41467-026-74594-9 (PMC13473598; doi:10.1038/s41467-026-74594-9)
Supplement: Supplementary file 1 [file 41467_2026_74594_MOESM1_ESM.pdf]

Supplementary Information

Supplementary Table 1. List of some NVS-SM1-induced exons with corresponding complementarity scores. Scores were calculated by summing the maximum number of possible H-bonds formed between the 5'ss of AGA exons and U1 snRNA. We also considered the possibility of an A_{-1} bulge formation, thus including the -4 position of the exon in the score calculation.

Gene name	chr.	start	end	U1 binding site	Complementarity score
HTT	4	3213622	3213736	CAGAGUAAGGGG	20
KCNT2	1	196317148	196317173	CAGAGUAAGAAA	21
LRRC42	1	53947862	53947981	UAGAGUAAGUUA	19
MLLT10	10	21728632	21728675	GAGAGUAAGAGA	18
ARHGAP1	11	46697021	46697069	CAGAGUAAGAGU	20
WNK1	12	895173	895196	GAGAGUAGGUGA	19

Supplementary Table 2. Oligonucleotides used in biochemical assays. Sequences are provided in 5 to 3 orientation. Modified nucleotides are indicated where applicable (2-aminopurine (2-AP), TAMRA label).

Name	Sequence (5–3)	Description	Assay
TAMRA-U1	[TAMRA]-GAUACUUACCUG	TAMRA-labeled U1 snRNA fragment	FP
HTT wt	GCAGAGUAAGGGG	*HTT* pseudoexon $5'$ss wild-type	FP
HTT C-4G	GGAGAGUAAGGGG	*HTT* pseudoexon $5'$ss, -4 C$>$G mutation	FP
HTT wt (2-AP)	GCAG[2AP]GUAAGGGG	*HTT* pseudoexon $5'$ss, 2-AP mutation	2-AP
HTT inverted (2-AP)	GCAC[2AP]GUAAGGGG	Modified *HTT* pseudoexon, 2-AP mutation	2-AP
U1 wt	GAUACUUACCUG	Wild-type U1 snRNA oligo	2-AP
U1 closed	GAUACUUACUCU	Closed U1 construct (fully complementary)	2-AP
U1 inverted	GAUACUUACGUG	Modified U1 snRNA oligo for inverted pairing	2-AP

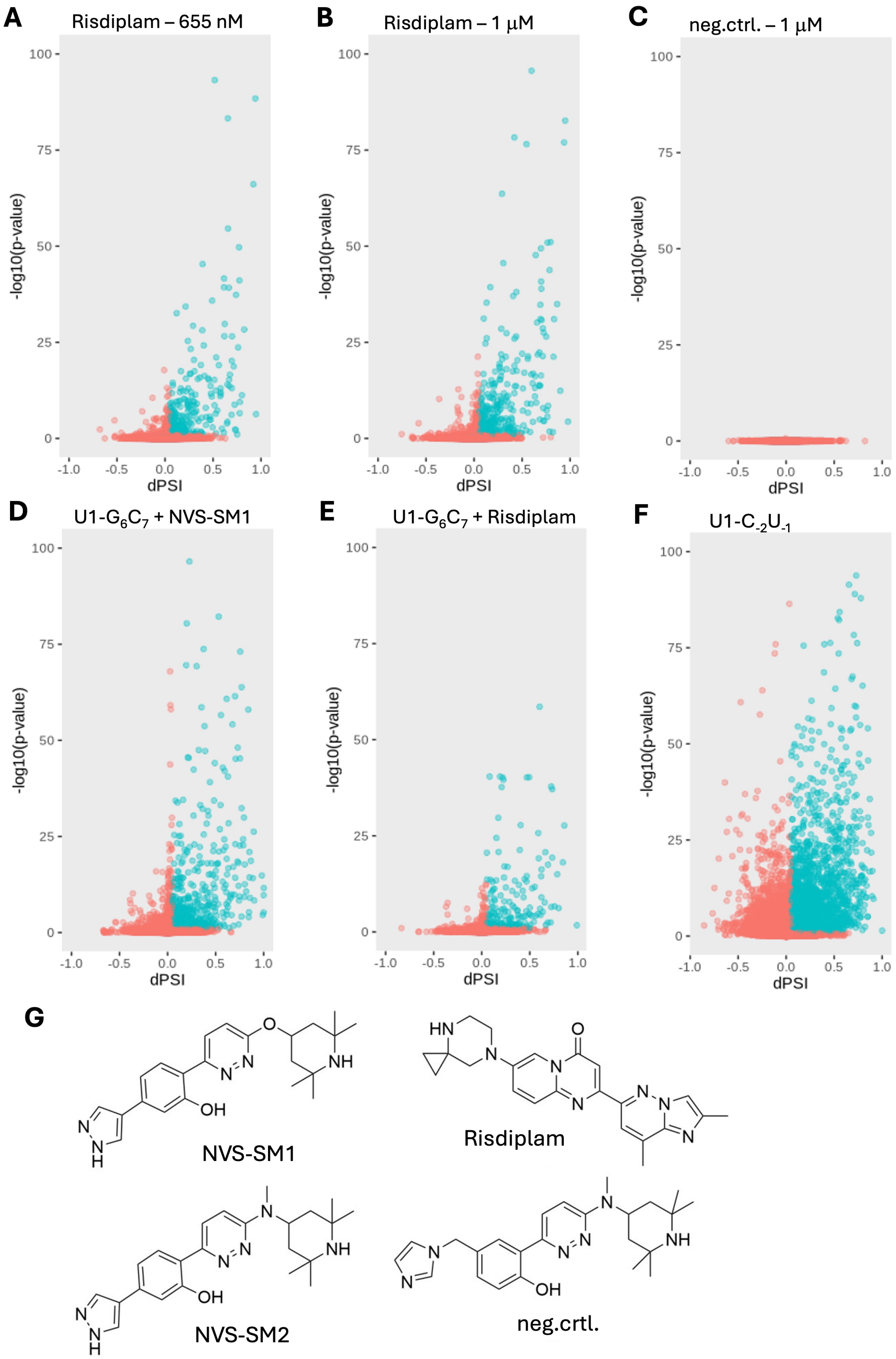

Supplementary Figure 1. Differential splicing analysis between whole transcriptome profiles of SH-SY5Y cells treated with (A) 655 nM Risdiplam, (B) 1 µM Risdiplam or (C) 1 µM of inactive compound, and SH-SY5Y cells treated with DMSO. Differential splicing analysis between whole transcriptome profiles of HeLa cells transfected with U1-G$_6$C$_7$ and treated with (D) 100 nM NVS-SM1 or (E) 1 µM Risdiplam, and transfected HeLa cells treated with DMSO. (F) Differential splicing analysis between whole transcriptome profiles of HeLa cells transfected with U1-C$_{-2}$U$_{-1}$ and treated with DMSO and HeLa cells transfected with canonical U1. For panels (A–F), exons highlighted in turquoise are considered included in response to treatment (dPSI > 0.05, adjusted p < 0.1). Statistical significance was assessed using a likelihood ratio test, and adjusted p values are reported. (G) Chemical structures of the reference compounds used in this study: NVS-SM1, NVS-SM2, Risdiplam, and an inactive compound used as control (neg.ctrl.).

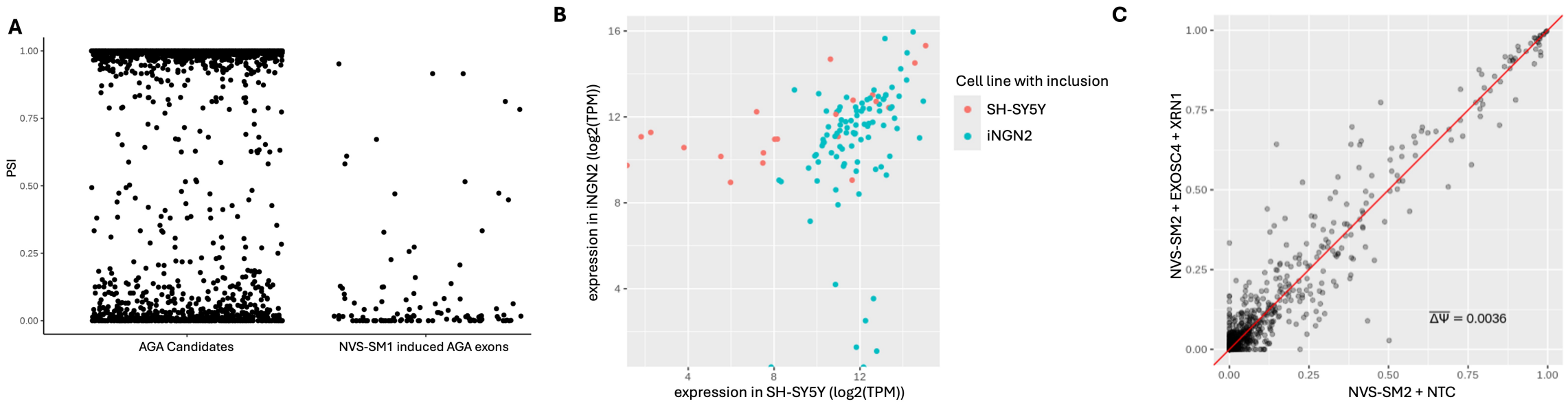

Supplementary Figure 2. (A) Detected baseline inclusion in SH-SY5Y cells in the absence of a compound. Left group (AGA Candidates) represents all 5'ss matching a $5'\text{-A}_{-3}\text{G}_{-2}\text{A}_{-1}\text{G}_{1}\text{U}_{2}\text{-}3'$ motif, identified as potential targets for NVS-SM1 in the human RNA-seq data collection from the Genotype-Tissue Expression Project. Right group (NVS-SM1 induced AGA exons) contains all AGA exons detected to be significantly induced in response to 25 nM NVS-SM1 treatment. Each point represents an individual exon. (B) Expression levels (log2(TPM)) of genes containing AGA-ending exons significantly induced only in SH-SY5Y cells or only in iNGN2 cells, respectively. Expression in iNGN2 cells shown on y-axis and expression in SH-SY5Y cells shown on x-axis. Each point represents one gene. (C) Scatter plot showing PSI values of AGA-ending exons in SH-SY5Y cells treated with 100 nM of the NVS-SM2, an analog of NVS-SM1 (Supplementary Figure 1G), in the presence of NMD inhibiting siRNAs (EXOSC4 + XRN1) or non-targeting control (NTC). Each point represents one exon. The red diagonal line indicates equal PSI values between the compared conditions and $\overline{\Delta\Psi}$ the mean change in inclusion.

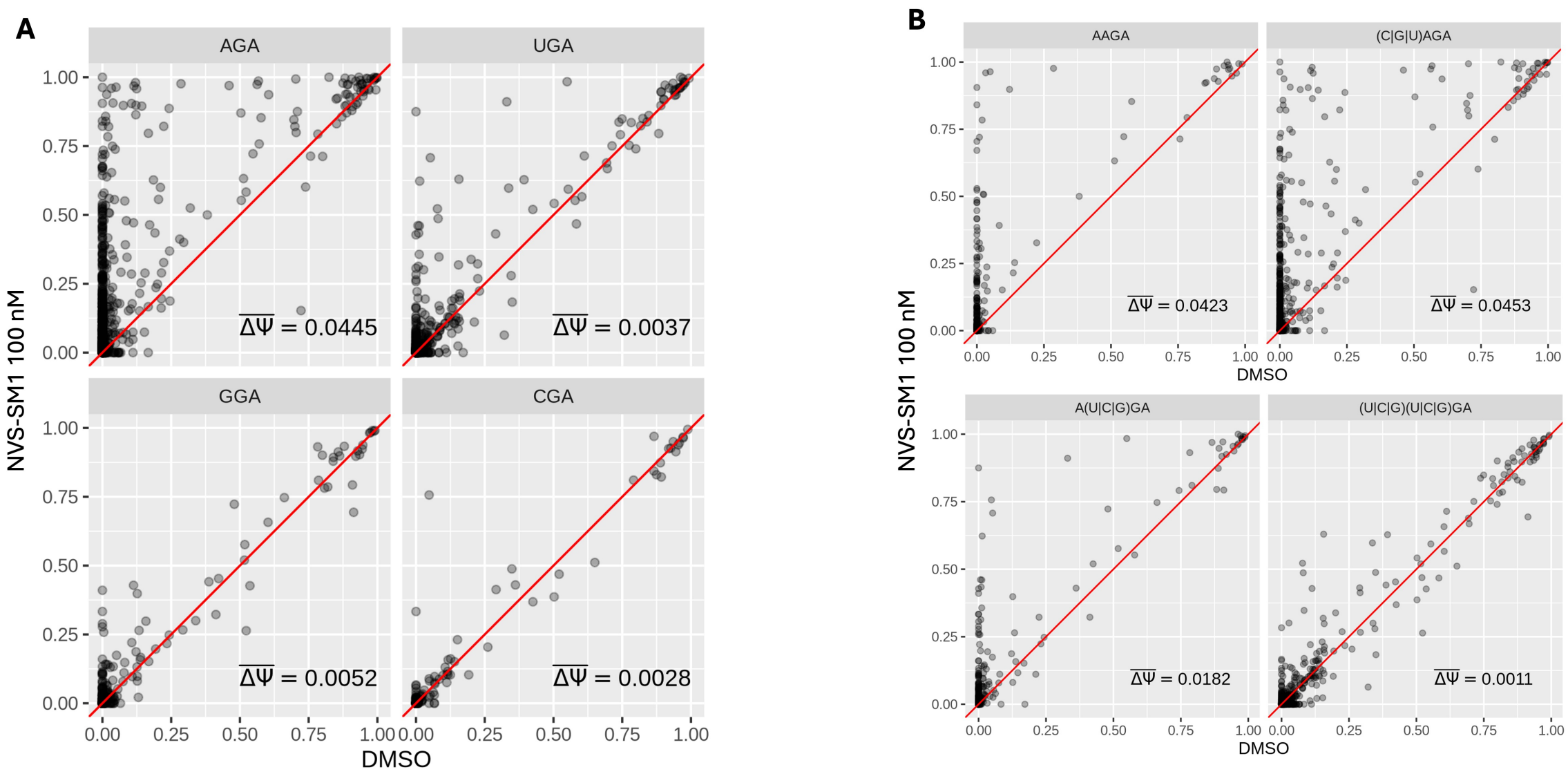

Supplementary Figure 3. Scatter plots showing PSI values of GA-ending exons in cells treated with 100 nM NVS-SM1 or DMSO. Each point represents one exon. (A) Exons grouped by their –3 position (AGA, UGA, GGA, or CGA). (B) Exons grouped according to adenine presence at the –3 and –4 positions: A at both positions (AAGA), A only at –3 ((C|G|U)AGA), A only at –4 (A(C|G|U)GA), or A absent at both ((C|G|U)(C|G|U)GA). The red diagonal line indicates equal PSI values between the compared conditions and $\overline{\Delta\Psi}$ the mean change in inclusion.

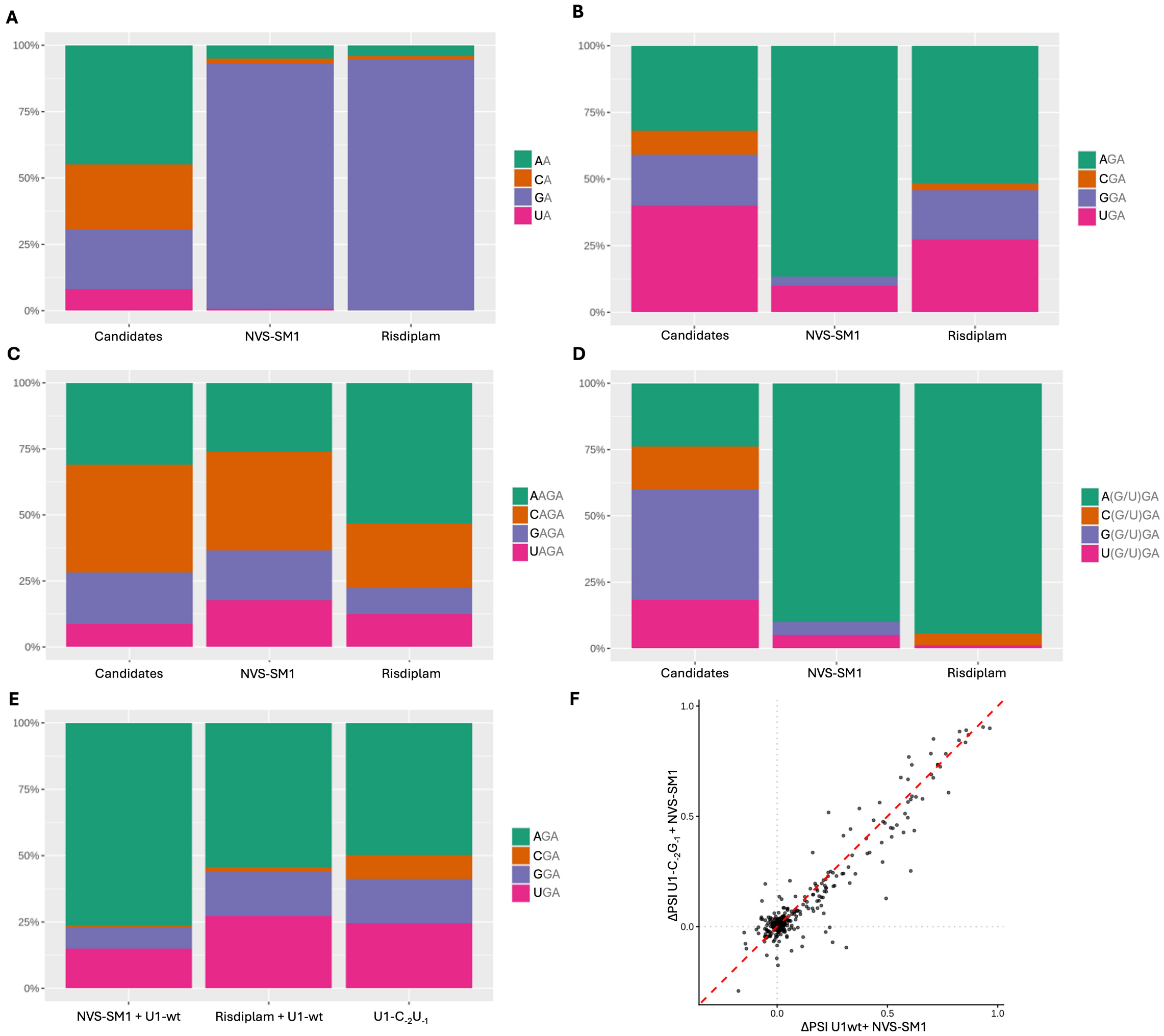

Supplementary Figure 4. (A) Frequency of each nucleotide at position -2 within all A-ending exons (Candidates) or all induced A-ending exons upon NVS-SM1 or Risdiplam treatment. (B) Frequency of each nucleotide at position -3 within all GA-ending exons (Candidates) or all induced GA-ending exons upon NVS-SM1 or Risdiplam treatment in SH-SY5Y cells. (C) Frequency of each nucleotide at position -4 within all AGA exons (Candidates) or all induced AGA exons upon NVS-SM1 or Risdiplam treatment. (D) Frequency of each nucleotide at position -4 of all UGA or GGA exons (Candidates) or all induced UGA or GGA exons upon NVS-SM1 or Risdiplam treatment. (E) Frequency of each nucleotide at position -3 of GA-ending exons induced in response to NVS-SM1 or Risdiplam treatment in HeLa cells transfected with wt U1 snRNA or in cells transfected with U1-$C_{-2}U_{-1}$ and treated with DMSO. Frequencies are calculated over all exons in each group. (F) Scatter plot comparing exon inclusion changes (ΔPSI) upon compound treatment in cells expressing canonical (U1-wt + NVS-SM1) or mutated U1 snRNA (U1-$C_{-2}G_{-1}$ + NVS-SM1). Each dot represents one splicing event. The red diagonal line indicates equal values between the compared conditions.

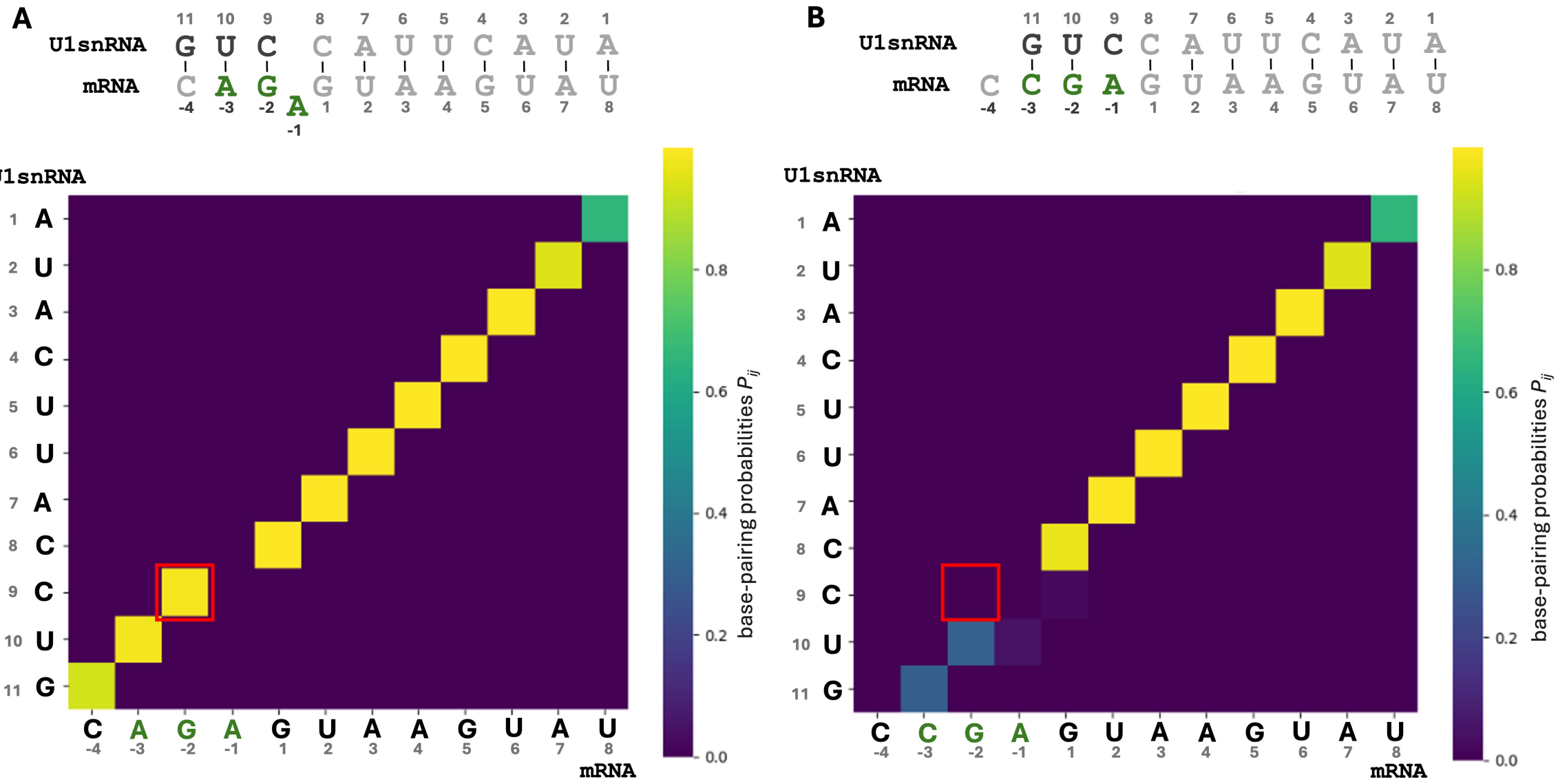

Supplementary Figure 5. Predicted U1–$5'$ splice site base-pairing probabilities for AGA and CGA exons. Cofold probability heatmaps showing predicted intermolecular pairing between U1 snRNA (rows) and the $5'$ splice-site region of the AGA exon (A) or CGA exon (B). The red box marks the expected interaction between the 2 G of the exon and the 9 C within the U1 snRNA that usually stabilizes the A−bulge. The corresponding base-pairing probabilities ρ for this position are: (A) 0.99 and (B) 0. No statistical test was performed.

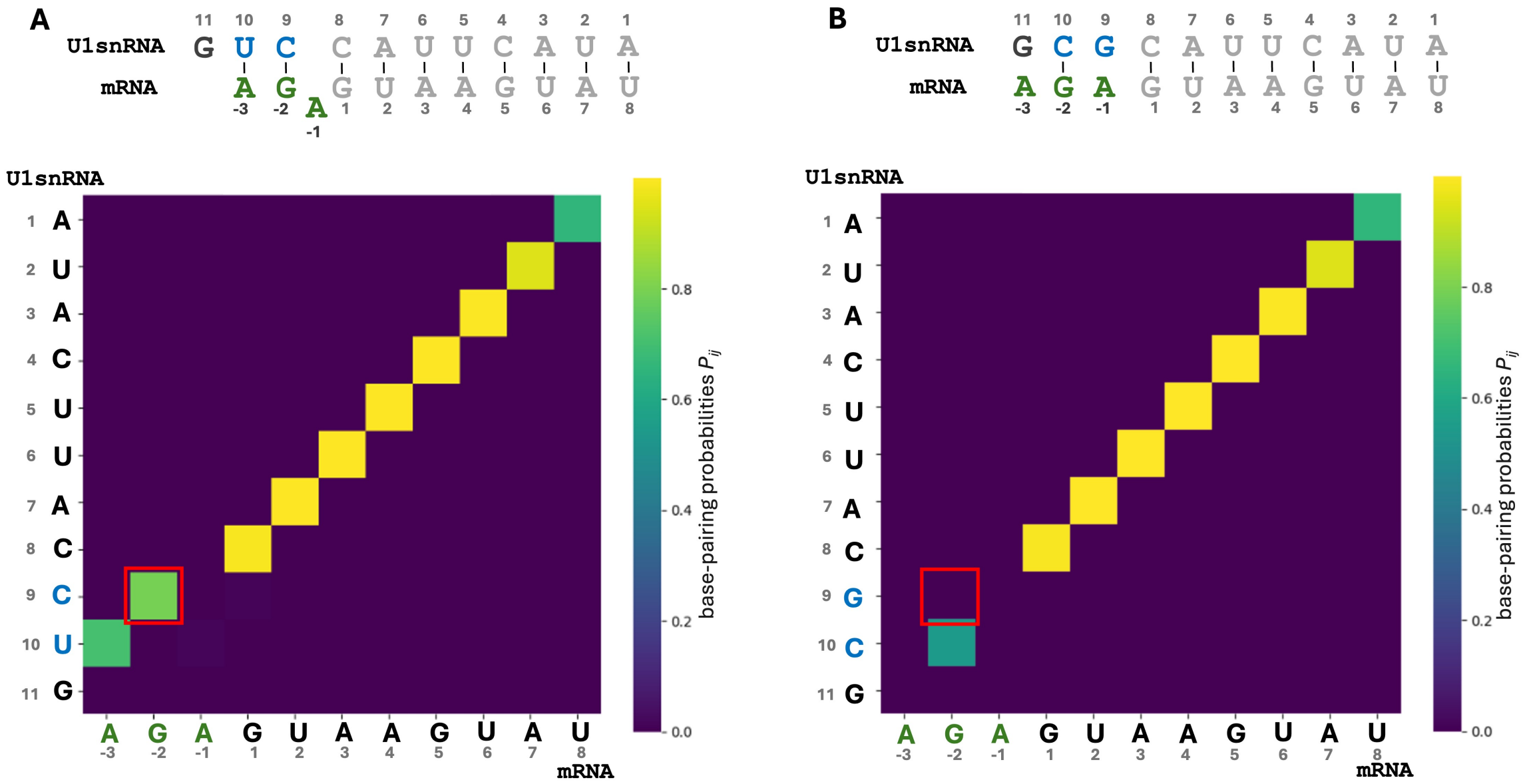

Supplementary Figure 6. Predicted U1–$5'$ splice site base-pairing probabilities for canonical and mutant U1 snRNA with an AGA exon. Cofold probability heatmaps showing predicted intermolecular pairing between U1 snRNA (rows) and the $5'$ splice-site region of the AGA exon (columns). (A) shows predictions using the canonical U1 snRNA sequence (free energy: 8.78 kcal/mol) and (B) shows predictions using the mutated U1 snRNA U1-C$_{-2}$G$_{-1}$ (free energy: 8.65 kcal/mol). The red box marks the expected interaction between the 2 nucleotide of the exon and the C within the U1 snRNA that usually stabilizes the A−bulge. The corresponding base-pairing probabilities ρ for this position are: (A) 0.79 and (B) 0. No statistical test was performed.

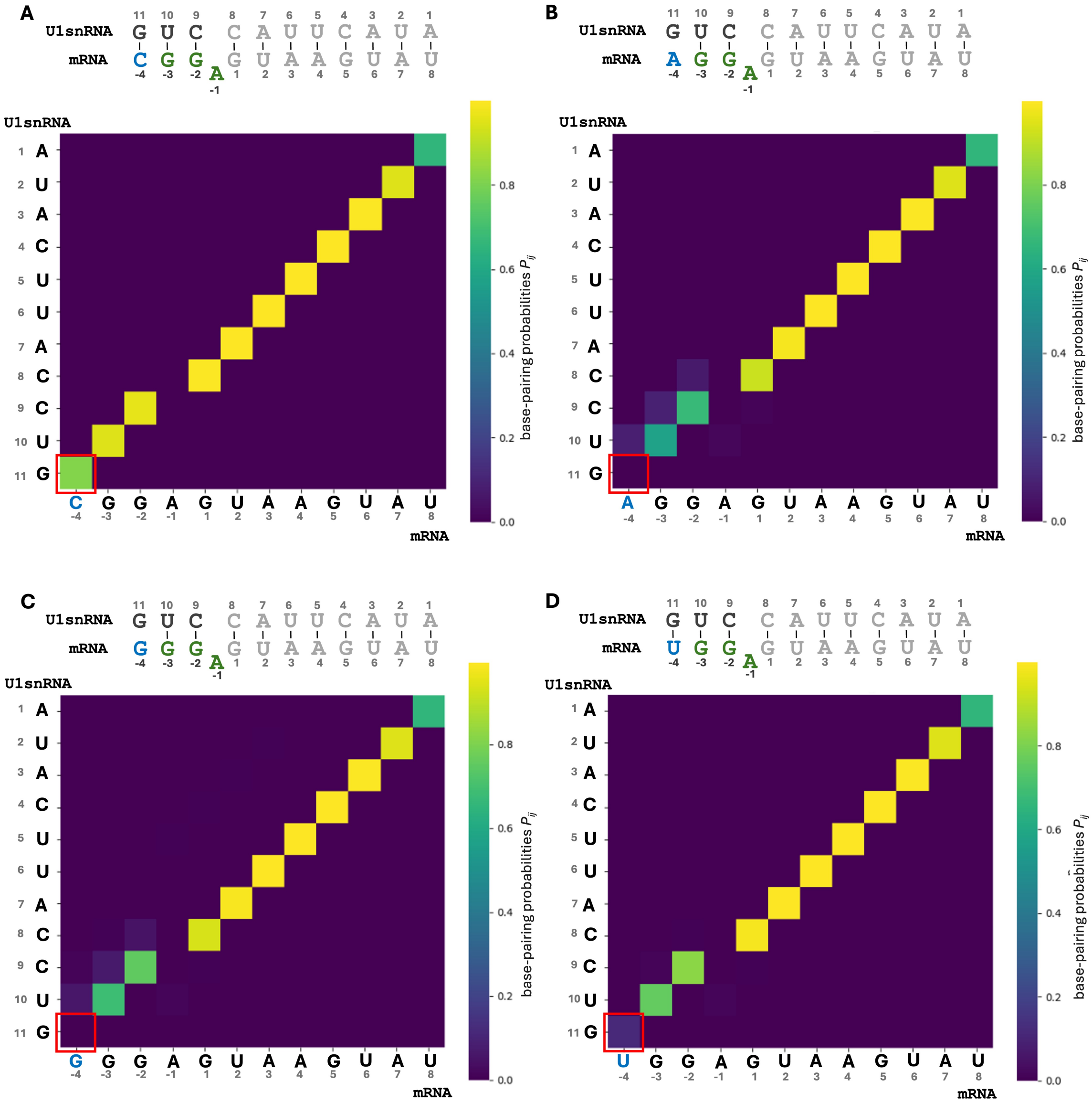

Supplementary Figure 7. Base-pairing probability maps for U1–5′ splice site interactions with different nucleotides at the 4 position. Cofold probability heatmaps show predicted intermolecular pairing between U1 snRNA (rows) and the 5 splice-site sequence (columns). A–D show the predicted interaction patterns for 4C, 4A, 4G, and 4U variants, respectively. The red box highlights the predicted pairing between the 4 nucleotide of the 5′ splice site and the 11G residue in U1 snRNA. The corresponding base-pairing probabilities ρ for this position are: (A) 0.81, (B) 0, (C) 0, and (D) 0.11. No statistical test was performed.

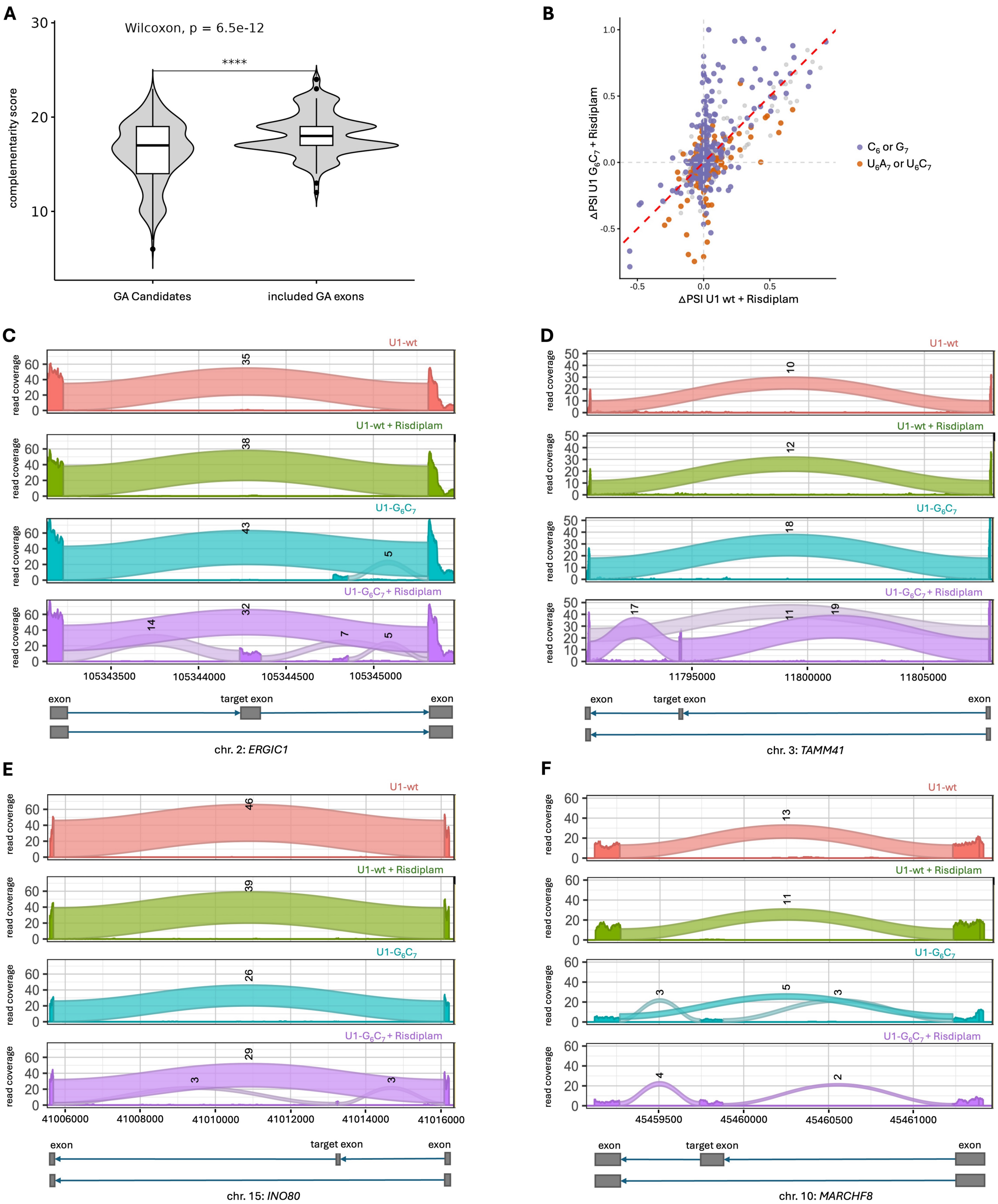

Supplementary Figure 8. (A) Violin plot showing the complementarity between the U1 snRNA and U1 binding sites of GA-ending exons included in response to Risdiplam (included GA exons) and those GA-ending exons that are potential targets for Risdiplam but are not included (GA candidates). The complementarity score was calculated by summing all possible H-bonds formed between the two RNAs. Box plots indicate the median (centre line), interquartile range (box), and whiskers extending to 1.5× the interquartile range; individual points beyond this range are shown as outliers. Statistical significance was assessed using a two-sided Wilcoxon rank-sum test ($p = 6.5 \times 10^{-12}$). The analysis was performed using a false discovery rate (FDR) threshold of 10%. (B) Scatter plot comparing exon inclusion changes (ΔPSI) upon compound treatment in cells expressing canonical (U1-wt + Risdiplam) or mutated U1 snRNA (U1-G_6C_7 + Risdiplam). The x-axis shows values for exons in cells transfected with canonical U1 and treated with 100 nM Risdiplam, and the y-axis shows values for the same exons in cells transfected with U1-G_6C_7 under identical treatment conditions. Exons with higher complementarity to the mutated U1 (carrying a C at position 6 or a G at position 7 of the U1 binding site) are highlighted in purple, whereas exons with higher complementarity to the canonical U1 are shown in orange. The diagonal line indicates equal ΔPSI between the two conditions. (C) Sashimi plots of RNA-seq results deriving from the same experiment as explained in Figure 3A. Plot highlights an exon within the gene *ERGIC1*. Significant inclusion only detected in cells transfected with U1-G_6C_7 and treated with 1 µM Risdiplam (dPSI = 0.36, FDR=10%). (D) Sashimi plots of RNA-seq results deriving from the same experiment as explained in Figure 3A. Plot highlights an exon within the gene *TAMM41*. Significant inclusion only detected in cells transfected with U1-G_6C_7 and treated with 1 µM Risdiplam (dPSI = 0.73, FDR=10%). (E) Sashimi plots of RNA-seq results deriving from the same experiment as explained in Figure 3A. Plot highlights an exon within the gene *INO80*. Low, but not significant inclusion detected in cells transfected with canonical U1 snRNA and 1 µM Risdiplam. Significant inclusion only detected in cells transfected with U1-G_6C_7 and Risdiplam treatment (dPSI = 0.17, FDR=10%). (F) Sashimi plots of RNA-seq results deriving from the same experiment as explained in Figure 3A. Plot highlights an exon within the gene *MARCHF8*. Significant inclusion detected in cells transfected with U1-G_6C_7 (dPSI = 0.79, FDR=10%) and boosted inclusion in cells transfected with U1-G_6C_7 and additional Risdiplam treatment (dPSI = 1.0, FDR=10%).

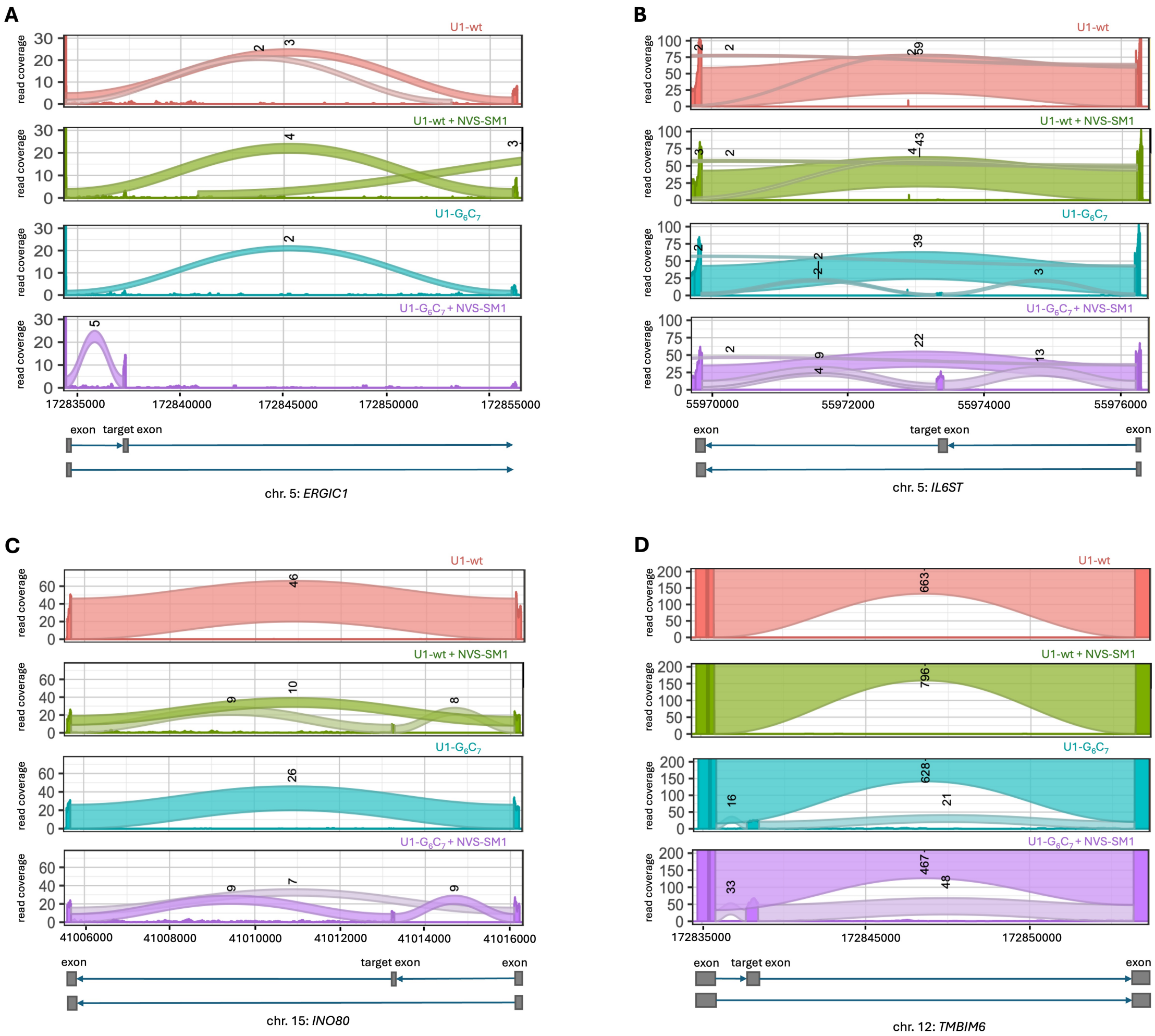

Supplementary Figure 9. (A) Sashimi plots of RNA-seq results deriving from the same experiment as in Figure 3A. Plot highlights an exon within the gene *ERGIC1*. Significant inclusion only detected in cells transfected with U1-G_6C_7 and treated with 100 nM NVS-SM1 (dPSI = 0.13, FDR = 10%). (B) Sashimi plots of RNA-seq results deriving from the same experiment as explained in Figure 3A. Plot highlights an exon within the gene *IL6ST*. Significant inclusion detected in cells transfected with U1-G_6C_7 (dPSI = 0.1, FDR=10%) and boosted inclusion in cells additionally treated with 100 nM NVS-SM1 (dPSI = 0.43, FDR=10%). (C) Sashimi plots of RNA-seq results deriving from the same experiment as explained in Figure 3A. Plot highlights an exon within the gene *INO80*. Significant inclusion detected in cells transfected with canonical U1 snRNA (U1-wt) and NVS-SM1 treatment (dPSI = 0.6, FDR=10%) and boosted inclusion in cells transfected with U1-G_6C_7 and NVS-SM1 treatment (dPSI = 0.7, FDR=10%). (D) Sashimi plots of RNA-seq results deriving from the same experiment as explained in Figure 3A. Plot highlights an exon within the gene *TMBIM6*. Significant inclusion detected in cells transfected with U1-G_6C_7 (dPSI = 0.06, FDR=10%) and boosted inclusion in cells additionally treated with 100 nM NVS-SM1 (dPSI = 0.12, FDR=10%).

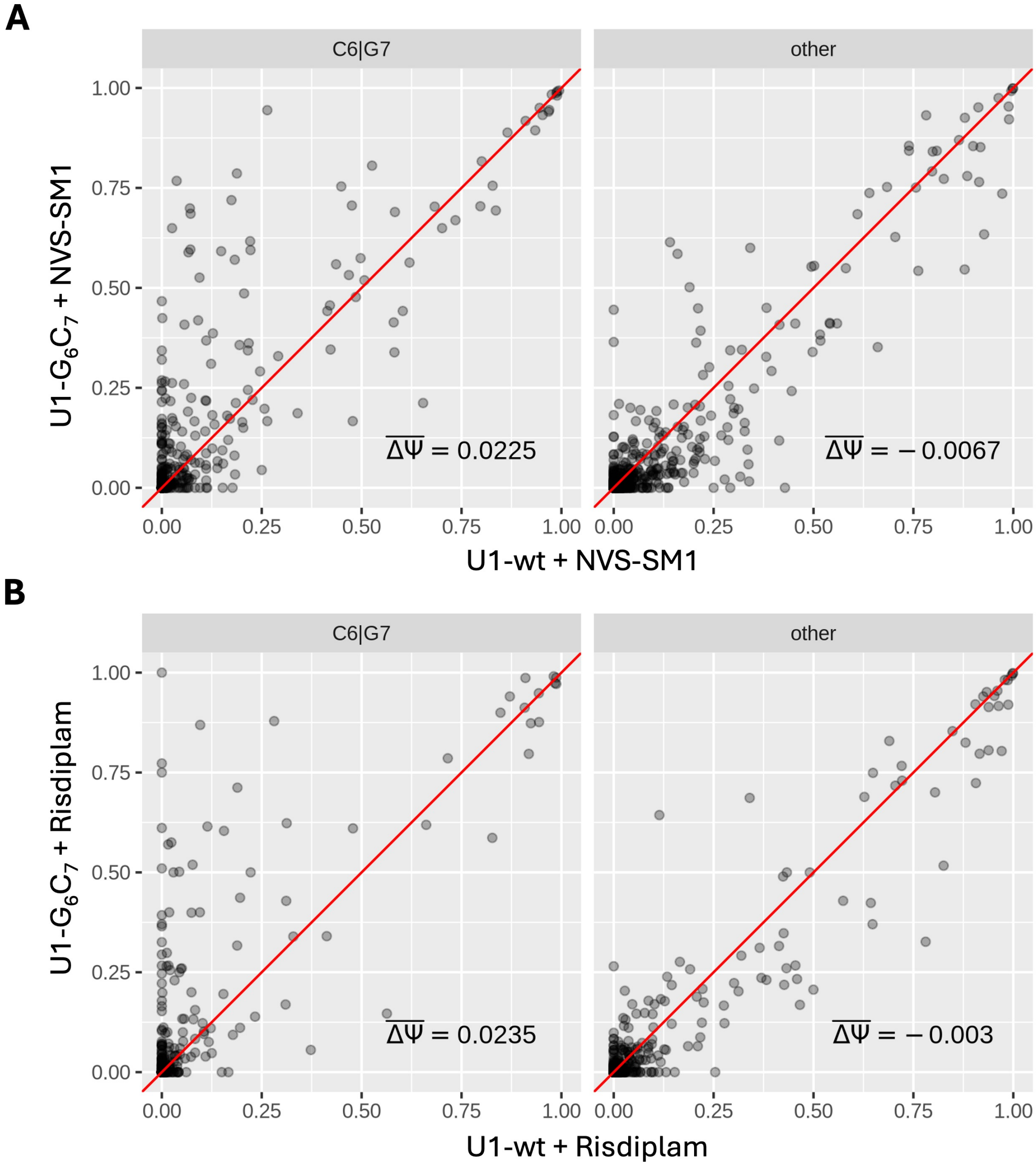

Supplementary Figure 10. Scatter plots showing PSI values of GA-ending exons, grouped by the presence of either a C at position +6 or a G at +7 (C6|G7) versus exons lacking one of these nucleotides (other). (A) Cells treated with NVS-SM1 and transfected with either U1-G_6C_7 or canonical U1 snRNA. (B) Cells treated with Risdiplam and transfected with either U1-G_6C_7 or canonical U1 snRNA. The red diagonal line indicates equal PSI values between the compared conditions and $\overline{\Delta\Psi}$ the mean change in inclusion. Each dot represents one splicing event.

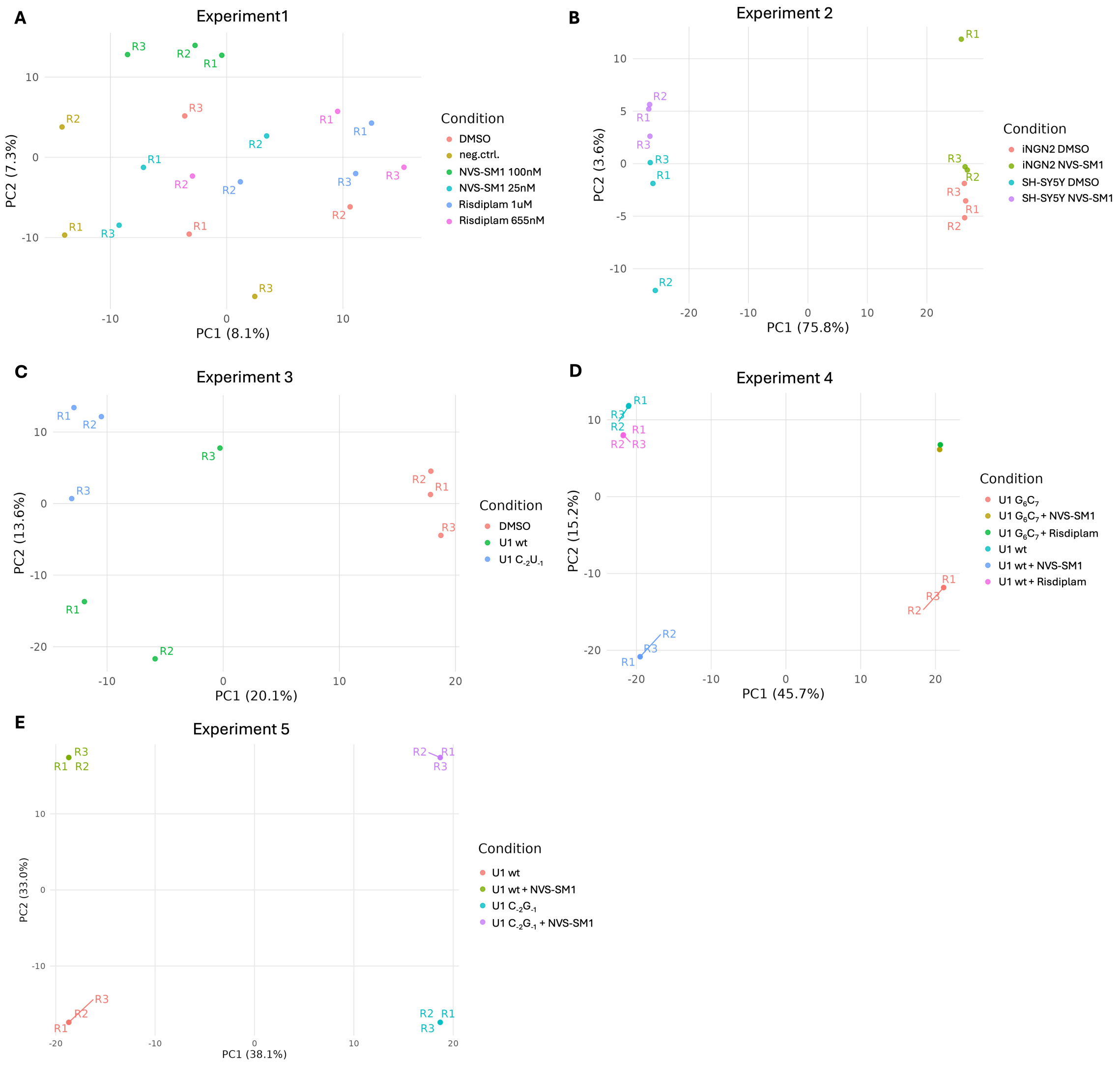

Supplementary Figure 11. Principal component analysis (PCA) of exon inclusion profiles. PCA was performed on the 1,000 most variable exons across samples. (A) SH-SY5Y cells treated with DMSO, negative control compound, Risdiplam, or NVS-SM1 at different concentrations, each in biological triplicates (R1–R3). (B) Comparison of compound-treated and untreated samples in different cell types (SH-SY5Y and iNGN2). (C) HEK293 cells expressing U1-wt or U1-C$_{-2}$U$_{-1}$ snRNA, with and without compound treatment. (D) HEK293 cells expressing either canonical U1 or mutated U1 snRNA (U1-G$_6$C$_7$), with and without compound treatment. (E) HEK293 cells expressing either canonical U1 or mutated U1 snRNA (U1-C$_{-2}$G$_{-1}$), with and without NVS-SM1 treatment (100 nM). Each point represents one replicate, and proximity indicates similarity in exon inclusion patterns.

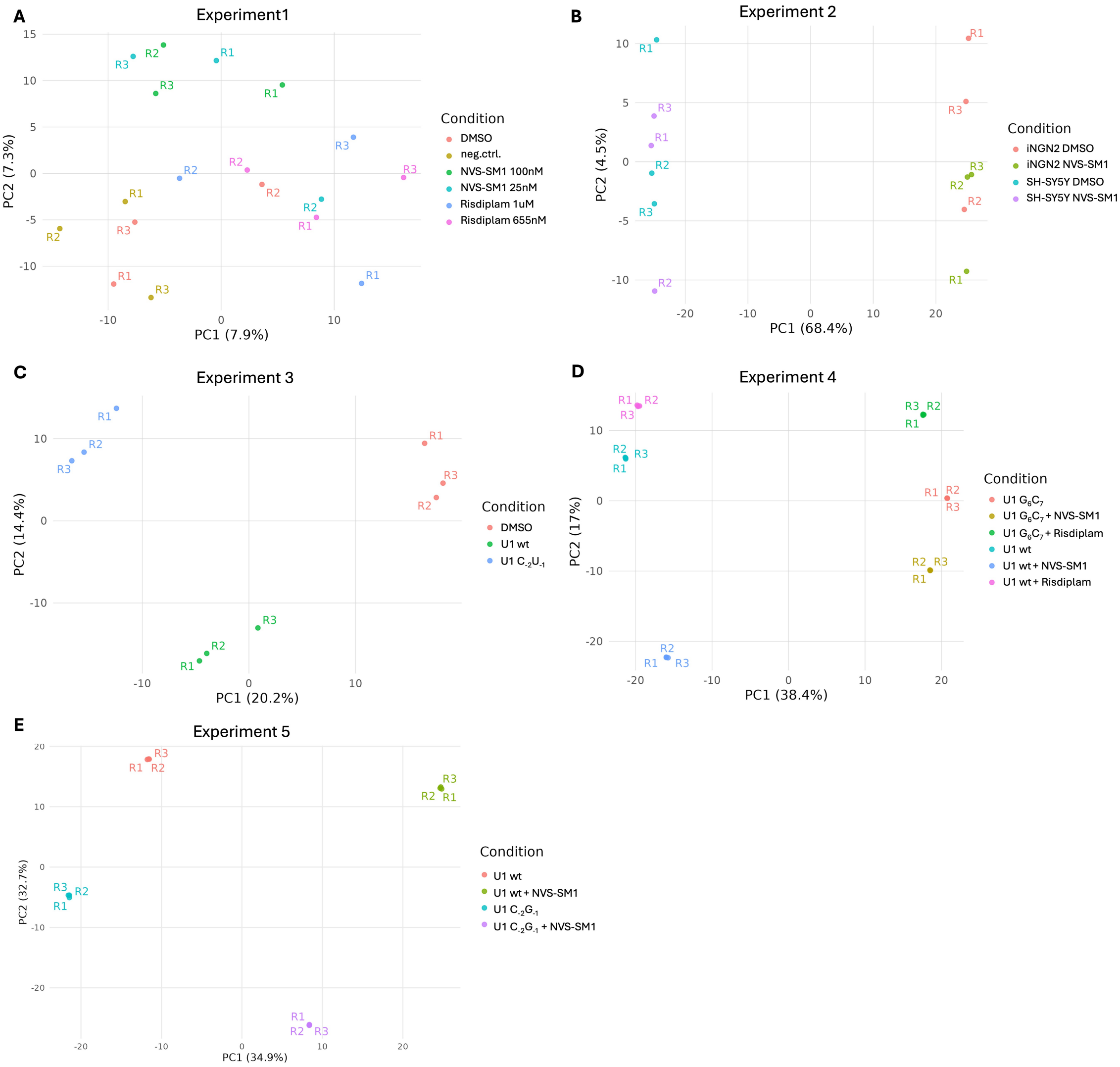

Supplementary Figure 12. PCA of GA-ending exons. (A) SH-SY5Y cells treated with DMSO, negative control compound, Risdiplam, or NVS-SM1 at different concentrations, each in biological triplicates (R1–R3). (B) Comparison of compound-treated and untreated samples in different cell types (SH-SY5Y and iNGN2). (C) HEK293 cells expressing U1-wt or U1-$C_{-2}U_{-1}$ snRNA, with and without compound treatment. (D) HEK293 cells expressing either canonical U1 or mutated U1 snRNA, with and without compound treatment. (E) HEK293 cells expressing either canonical U1 or mutated U1 snRNA (U1-$C_{-2}G_{-1}$), with and without NVS-SM1 treatment (100 nM). Each point represents one replicate, and proximity indicates similarity in exon inclusion patterns.

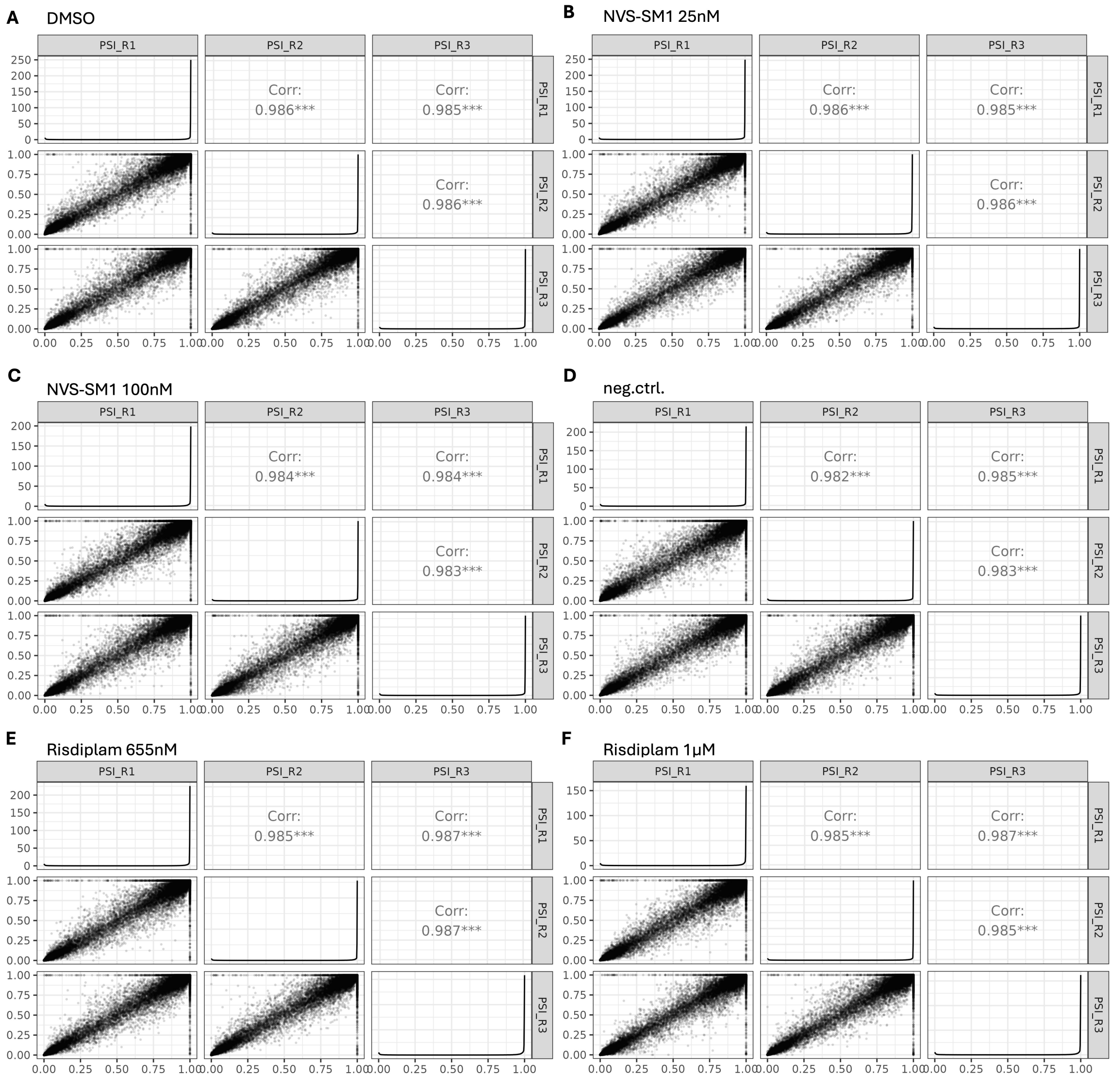

Supplementary Figure 13. Reproducibility of exon inclusion measurements across biological replicates. Pairwise correlations of PSI values between triplicates for SH-SY5Y cells treated with (A) DMSO, (B) 25 nM NVS-SM1, (C) 100 nM NVS-SM1, (D) negative control compound, (E) 655 nM Risdiplam, and (F) 1 µM Risdiplam. Each panel shows pairwise scatter plots of PSI values between replicates (R1–R3), with Pearson correlation coefficients indicated. High correlations across all conditions demonstrate the reproducibility of exon inclusion quantification.

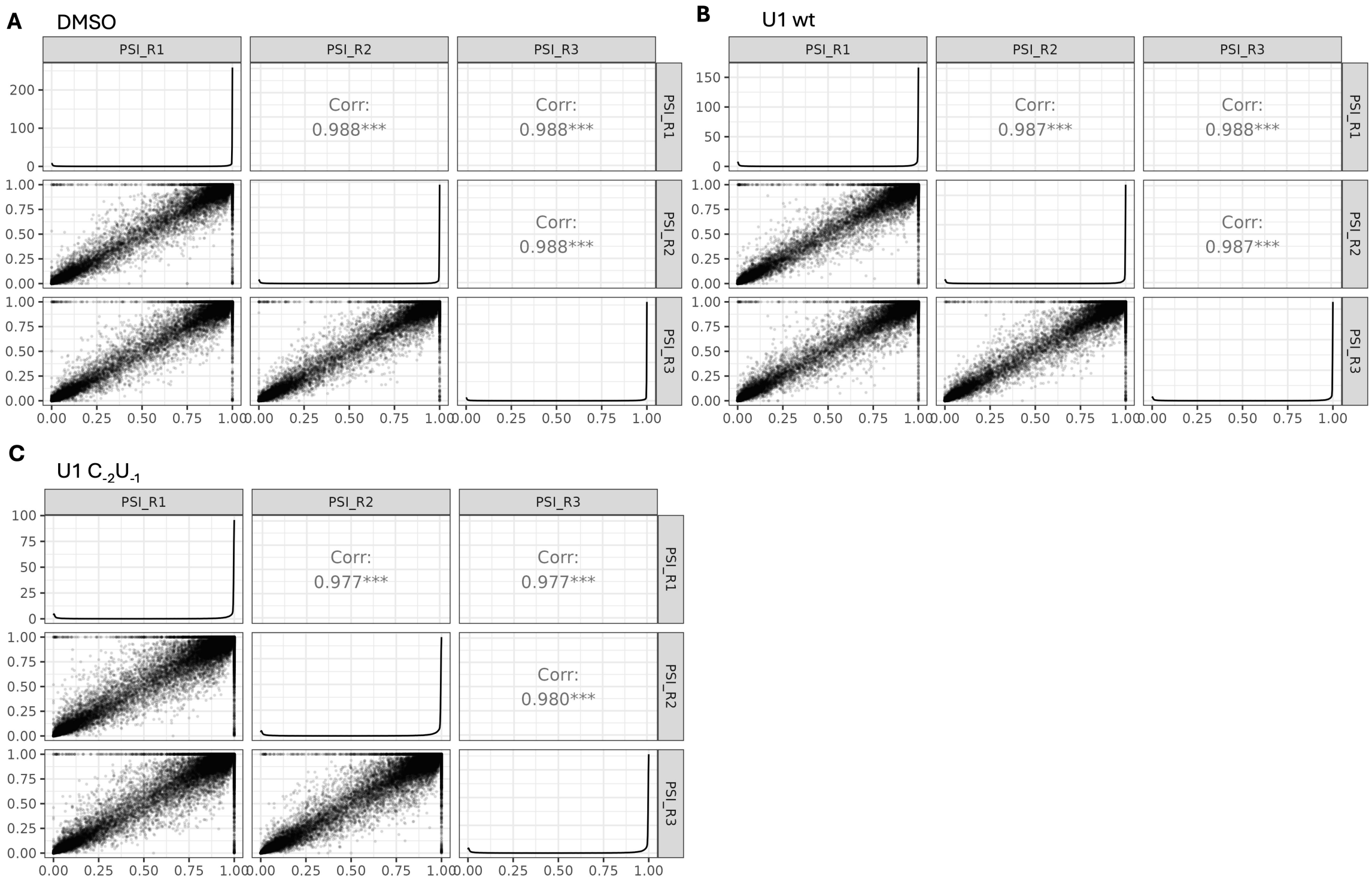

Supplementary Figure 14. Reproducibility of exon inclusion measurements across biological replicates in U1-transfected cells. Pairwise correlations of PSI values between replicates for cells transfected with (A) DMSO control, (B) canonical U1 snRNA (U1 wt), and (C) mutated U1 snRNA (U1-C$_{-2}$U$_{-1}$). Each panel shows pairwise scatter plots of PSI values between replicates (R1–R3), with Pearson correlation coefficients indicated. High correlation coefficients across all samples confirm the reproducibility of exon inclusion quantification.

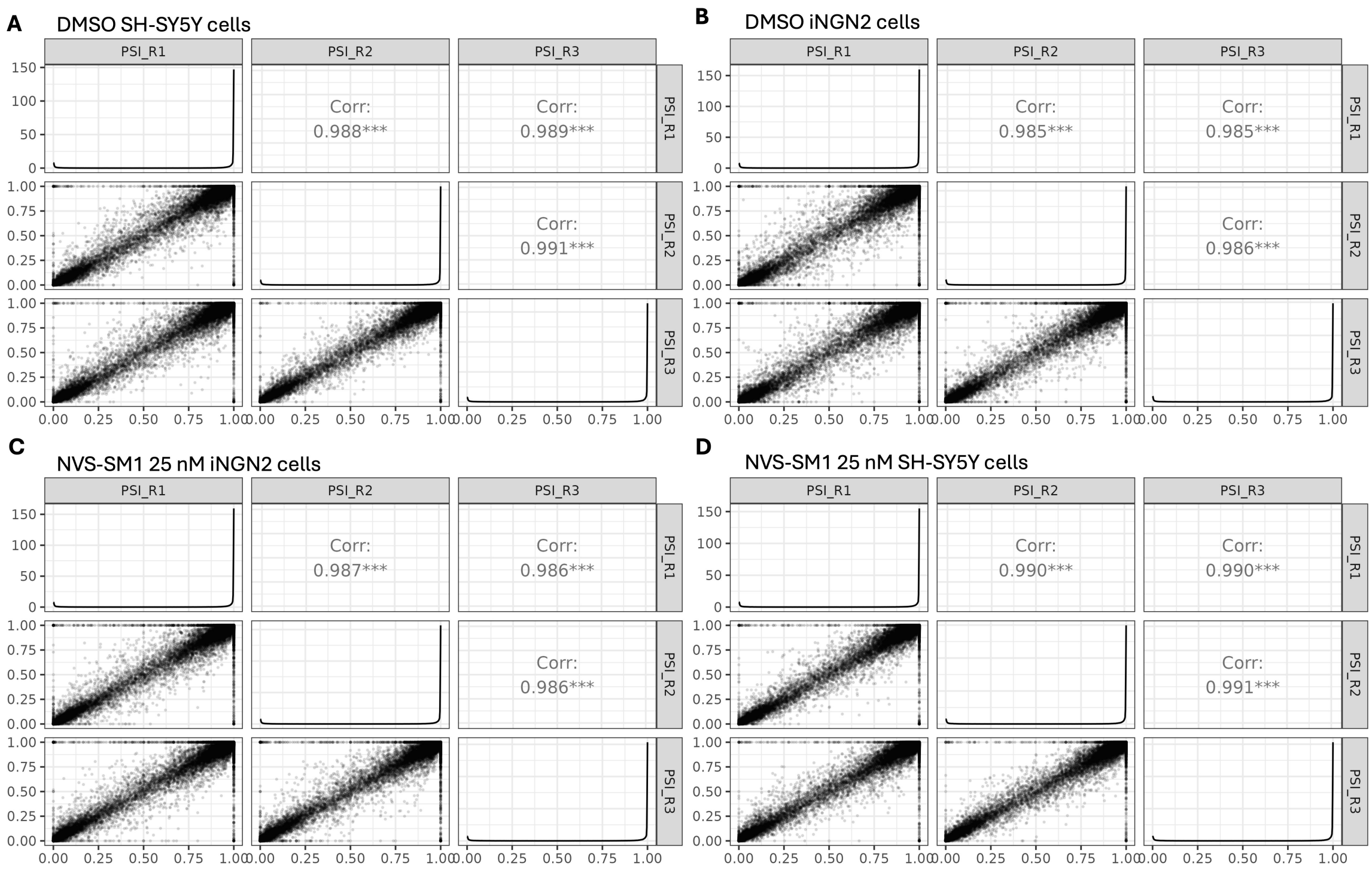

Supplementary Figure 15. Reproducibility of exon inclusion measurements across biological replicates in SH-S5Y5 and iNGN2 cells. Pairwise correlations of PSI values between replicates for SH-S5Y5 and iNGN2 cells treated with either DMSO (A and B) or 25 nM NVS-SM1 (C and D). Each panel shows pairwise scatter plots of PSI values between replicates (R1–R3), with Pearson correlation coefficients displayed. High correlations across all conditions confirm the robustness and reproducibility of exon inclusion quantification.

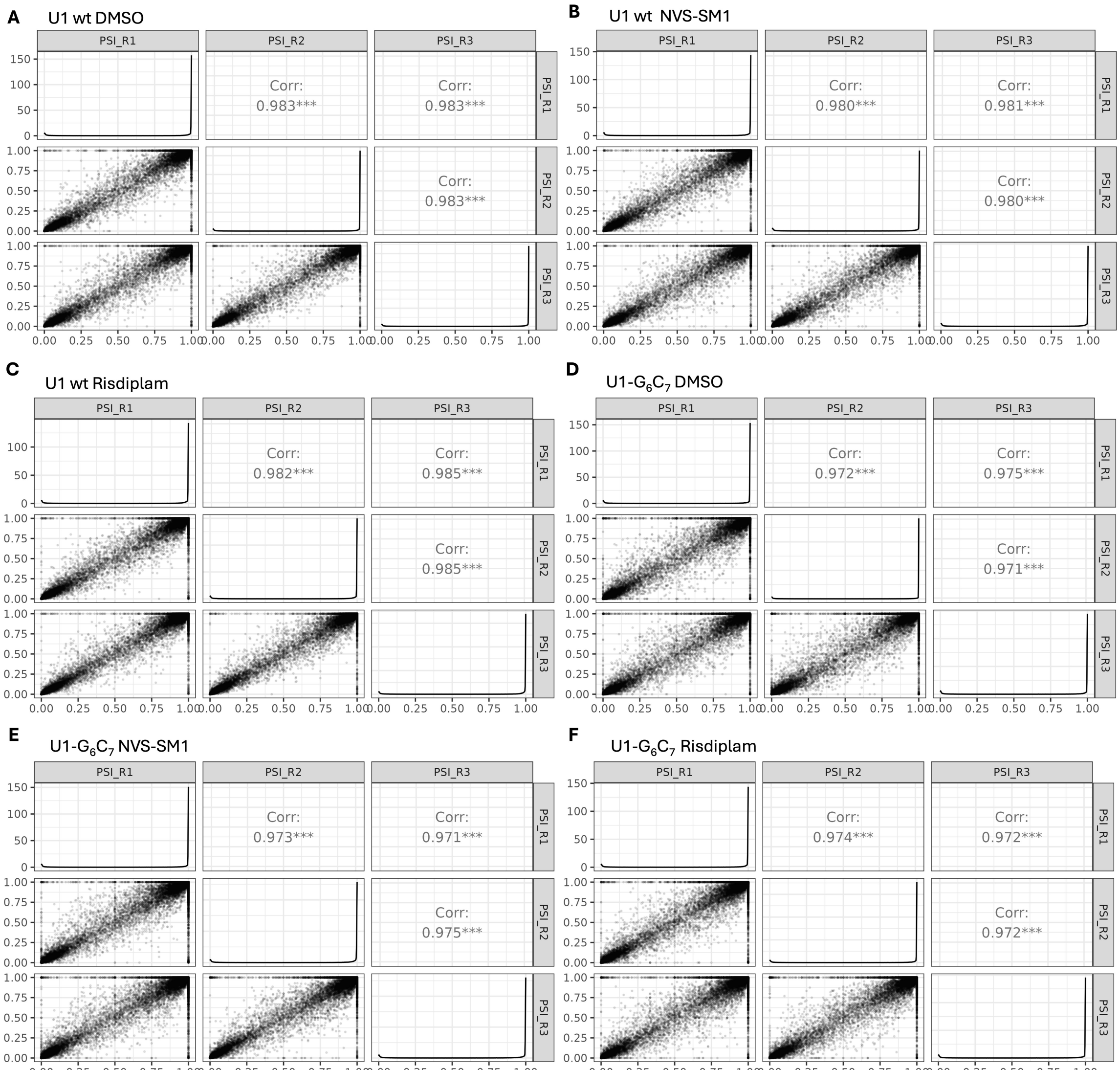

Supplementary Figure 16. Reproducibility of exon inclusion measurements across biological replicates in HEK293 cells expressing canonical or mutant U1 snRNA. Pairwise correlations of PSI values between replicates for cells expressing either wild-type U1 (A–C) or U1-G_6C_7 (D–F), treated with DMSO, NVS-SM1, or Risdiplam as indicated. Each panel shows pairwise scatter plots of PSI values between replicates (R1–R3), with Pearson correlation coefficients displayed. High correlations across all conditions confirm the robustness and reproducibility of exon inclusion quantification.

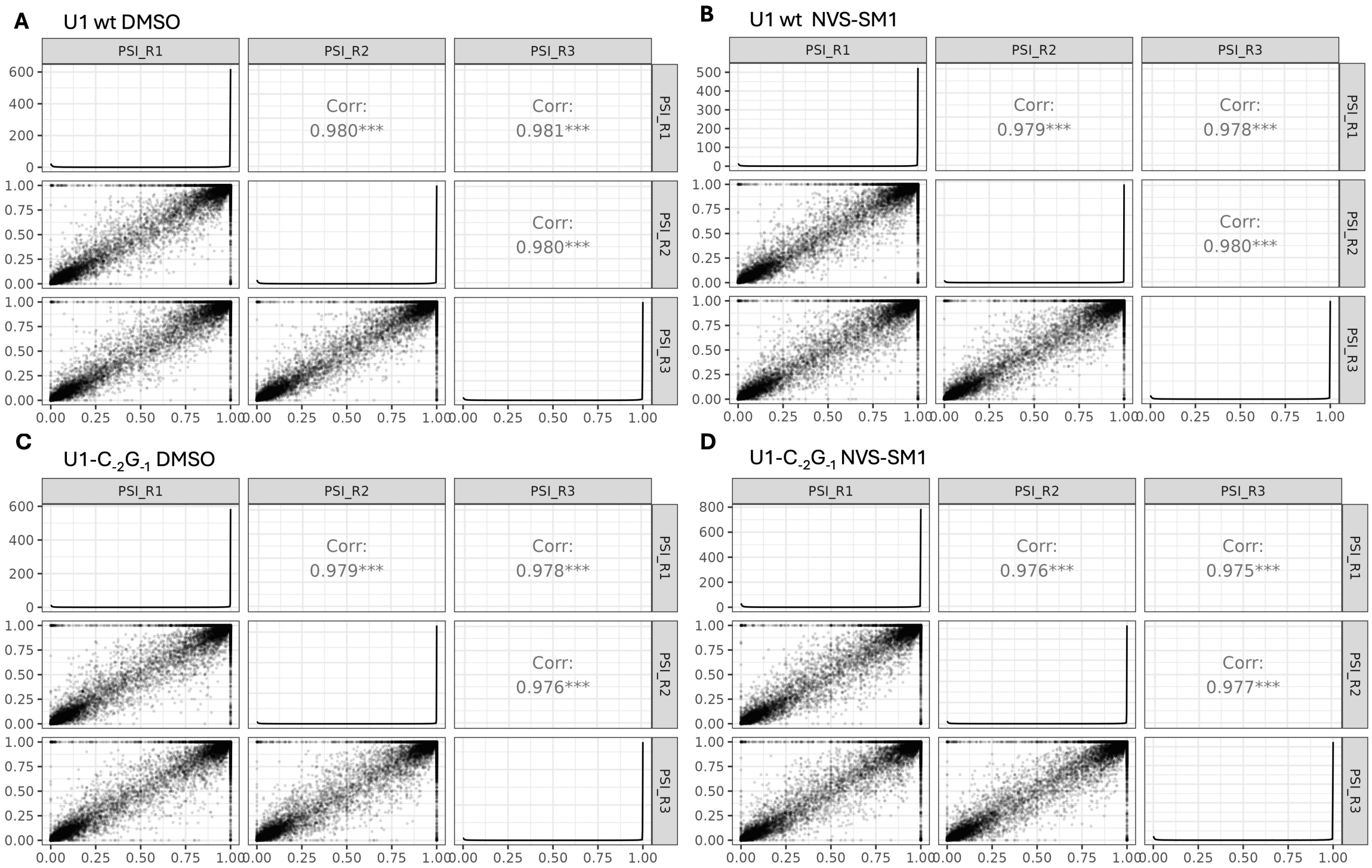

Supplementary Figure 17. Reproducibility of exon inclusion measurements across biological replicates in SH-SYS5 cells expressing canonical or mutant U1 snRNA. Pairwise correlations of PSI values between replicates for cells expressing either wild-type U1 (A–B) or U1-$C_{-2}G_{-1}$ (C–D), treated with DMSO or NVS-SM1 as indicated. Each panel shows pairwise scatter plots of PSI values between replicates (R1–R3), with Pearson correlation coefficients displayed. High correlations across all conditions confirm the robustness and reproducibility of exon inclusion quantification.

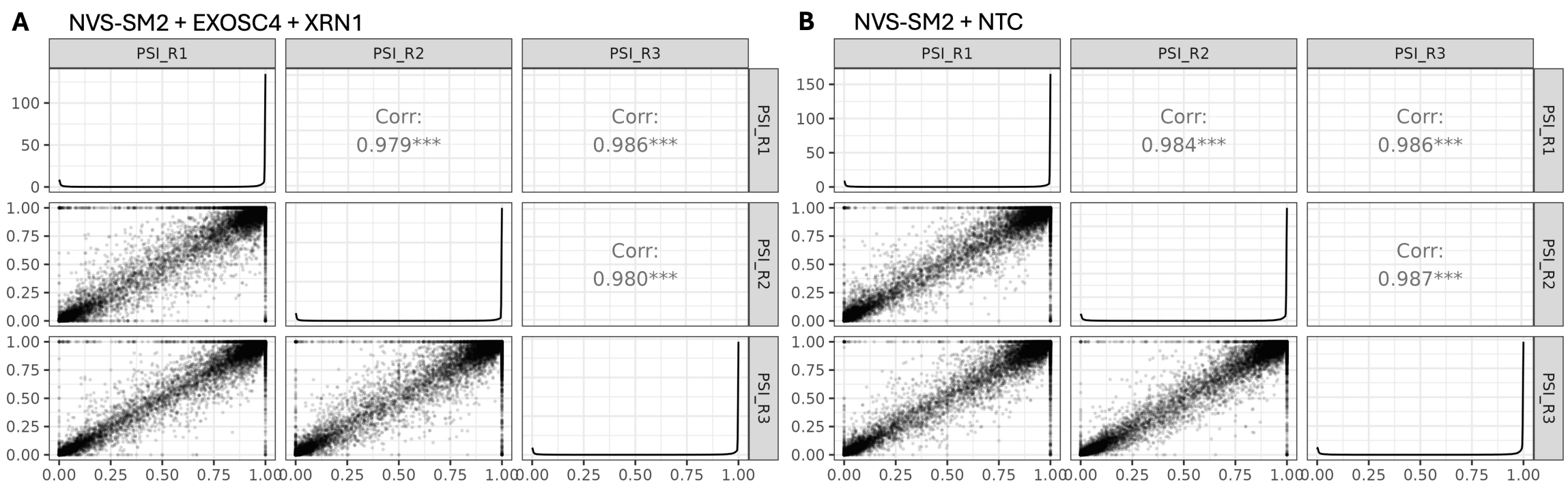

Supplementary Figure 18. Reproducibility of exon inclusion measurements across biological replicates in SH-SYS5 cells expressing canonical or mutant U1 snRNA. Pairwise correlations of PSI values between replicates for cells with XRN1 and EXOSC4 knockdown (A) or transfected with non-targeting control (B), treated with 100 nM of NVS-SM2. Each panel shows pairwise scatter plots of PSI values between replicates (R1–R3), with Pearson correlation coefficients displayed. High correlations across all conditions confirm the robustness and reproducibility of exon inclusion quantification.
