# Supplementary material for: Specificity and exon target space of splicing modifying compounds
Source: Nat Commun. 2026 Jul 4;17:8391. doi: 10.1038/s41467-026-74594-9 (PMC13473598; doi:10.1038/s41467-026-74594-9)
Supplement: Supplementary file 3 [file 41467_2026_74594_MOESM3_ESM.pdf]

nature portfolio

Peer Review File

Specificity and exon target space of splicing modifying compounds

Corresponding Author: Dr Alejandro Reyes

This file contains all reviewer reports in order by version, followed by all author rebuttals in order by version.

Version 0:

Reviewer comments:

Reviewer #1

(Remarks to the Author)
In this manuscript, Lenkeit et al. investigate the sequence determinants of the splice-modifying drugs risdiplam and branaplam using transcriptome profiling and various biochemical assays. They characterize the sequence features of exons activated by each drug, and observe that the presence of AGA at positions −3 to −1 is insufficient for activation by branaplam activity. They conclude that sufficiently stable binding of the U1 snRNP to 5′ splice sites is also required for sensitivity to branaplam. These hypotheses are tested in a variety of ways, including by using ectopic expression of mutated U1 snRNAs followed by transcriptome profiling, as well as by in vitro binding assays with variant U1 snRNAs and variant 5′ splice site RNAs.

My overall assessment is that this manuscript offers useful data relating to the action and specificity of splice-modifying small molecules drugs. However, the authors do not clearly articulate the open questions they address in this work, nor do they properly position their results within the context of existing literature. Indeed, it is questionable how novel their key results are. The data analysis approach used also has significant limitations that limit the interpretability of the results.

Major points
1. Novelty. The authors do not present a clear view of the existing literature on the specificities of splice-modifying drugs. They also do not adequately discuss the novelty of their findings in light of this literature.
a. The authors present novel in vitro data, based on measurements of 2-AP fluorescence, that generally support the -1A “bulge repair” mechanism for drug specificity. However, the bulge repair mechanism is not novel – it was proposed by Campagne et al. (2019) based on NMR structural data, and has found further support in the works Malard et al. (2024) and Ishigami et al. (2024).
b. The authors show that there are additional sequence determinants of the activity of branaplam at positions -4, +6 and +7 of the 5’ splice site. This is shown using transcriptomic profiling under ectopic overexpression of modified U1 sequences, as well as in vitro binding experiments. While these confirmatory data are valuable, the importance of the -4 and +6 positions have already been reported (e.g., in Ishigami et al. 2024).
c. The suggestion that drug activity is influenced by gene expression would, in principle, represent a novel and intriguing finding. But the observed association is more parsimoniously explained by limited statistical power to detect splicing changes in lowly expressed genes due to reduced sequencing depth. Stronger evidence would be required to support this claim.
2. Interpretability.
a. The analytical framework used in this study is not well suited to disentangling whether sequence mutations influence drug activity through modulation of the strength of the U1–5′ splice site interaction or by altering the strength of drug binding to the U1–5′ splice site complex. Prior work has already demonstrated that certain mutations, such as −4A and mutations at +4 and +5, can affect not only basal U1–5′ splice site binding but also drug activity. Disentangling these distinct effects requires quantitative modeling, e.g. as carried out in Ishigami et al. 2024. But as manuscript does not present any quantitative modeling, I do not see how these effects can be distinguished.
b. The authors rely primarily on identifying sets of “significantly activated” exons, a strategy that is heavily confounded by variable statistical power across genes. More generally, the lack of statistical significance is repeatedly misinterpreted throughout the manuscript as evidence for the absence of an effect. For example, the claim that AGA at -3 to -1 is insufficient for branaplam sensitivity is based solely on a failure to detect significant differences across all AGA exons. Similarly, the conclusion that branaplam exhibits different sequence-specific effects under ectopic expression of wild-type versus mutant

U1 is derived from similar reasoning. While these claims may be true, such arguments are not statistically valid. A more appropriate approach would be to analyze the quantitative effect sizes of drug responses directly, rather than relying on binary significance thresholds.

Minor points
1. The authors should provide a more thorough assessment of data quality. For example, scatterplots of estimated exon PSI across biological replicates and across conditions would help demonstrate reproducibility. Similarly, a PCA or related analysis should be used to show that drug treatment accounts for most of the experimental variance between samples.
2. The Methods section should specify the number of biological replicates for each RNA-seq experiment. Line 191 refers to a “single experiment,” but it is unclear whether this means a single biological replicate or a single culture batch.
3. The manuscript reports that −4C enhances interaction with U1, whereas −4A is suggested to act via direct drug interaction. To strengthen this claim, the authors should show whether −4A affects U1 binding in the absence of drug, analogous to the analysis presented for −4C. This would provide additional orthogonal evidence that −4A does not exert its effect via stabilization of the U1–5′ splice site interaction.
4. The sashimi plots presented for selected exons are interesting, but the generality of these observations should be demonstrated quantitatively. Specifically, scatterplots comparing PSI values under basal and drug conditions in wild-type versus G6,C7 U1 backgrounds should be shown for all exons with complementary 5′ splice site sequences. Furthermore, it should be tested whether exons complementary to the wild-type U1 positions 6 and 7 are preferentially activated in the presence of the wild-type U1 compared with the G6,C7 mutant.
5. The in vitro experiments convincingly show that branaplam stabilizes RNA duplexes only when −1A is bulged out. However, it remains unclear whether this mechanism operates in the spliceosomal context. Additionally, the authors could (for example) test whether ectopic expression of modified U1 containing −2C,−1U not only increases basal PSI but also reduces the effect of branaplam on the splicing efficiency of an AGA exon minigene or endogenous exon.
6. Note all of the raw sequencing data is currently available. Only RNA seq samples from HeLa cells treated with branaplam or ectopically expressing modified −2C,−1U appear to be deposited. Data from experiments performed on SH-SY5Y and iNGN2 cell lines, and with the U1-C6G7 treated HeLa cells, appear to be missing.
7. The code used to analyze the data should be made publicly available, e.g. on GitHub.
8. The labels for panels B and C are switched in legend of Figure 2B.
9. Typo: line 116 should read “an NMR structure,” not “a NMR.”
10. Line 163: the Snaptron tool should be cited appropriately (Wilks et al., Bioinformatics, 2018).

Reviewer #2

(Remarks to the Author)
The authors provide genome-wide sequencing work in order to decipher the mechanism of action of small molecule compounds (risdiplam and branaplam) in enhancing alternative splicing of specific alternative exons. These data confirm the molecular mechanism proposed by the structural work of Campagne et al, but provide these using complementary methods namely RNA sequencing and the modification of U1snRNA. Overall the key element is the presence of a A-1 bulge within a stable RNA duplex.
Overall the data are sounds and I cannot think of particular criticism to make.
The discussion is rather short and may benefit from mentioning other proposed mechanisms suggested in the literature where an additional motif purine rich is proposed as well for example. How are the data here agree or disagree ?

Reviewer #3

(Remarks to the Author)
This manuscript examines the underlying basis for the limited number of genes affected by two approved drugs for the treatment of SMA (risdiplam and branaplam (NVS-SM1)). Previous analysis of the basis for the enhancement of SMN2 exon 7 inclusion induced by either compound revealed an alteration in RNA structure upon compound binding that enhanced interaction with U1 snRNA. However, analysis of the sequence that confers sensitivity to risdiplam reveal that it occurs at a high frequency within RNA (~58000 potential exons) but drug treatment is shown to induce changes in splicing of only 609 exons. To explore the basis for this apparent discrepancy, the authors have investigated additional sequence features that confer regulation by NVS-SM1. Analysis by this group identified 8718 potential 5’ss that matched the AGA/GU motif required for regulation, of which 125-304 were affected by NVS-SM1 addition. Of the other sites, 334 AGA exons showed high levels of inclusion in the absence of compound. One feature common to AGA exons affected by NVS-SM1 was ~90 times higher level of expression than unaffected RNAs, suggesting that RNA abundance is a critical factor for the response. Another possible factor affecting AGA exon recognition is the extent of base pairing with U1 snRNA. To explore this possibility, the authors examine how extent of such base pairing of AGA exons impacts usage. Calculation of maximum H-bonds with U1 snRNA among AGA exons revealed a correlation with the response to either risdiplam or NVS-SM1 addition. As a test of the hypothesis, expression of modified U1 snRNA (U1-G6C7) resulted in an increase in AGA exons affected by compound addition, the exons affected showing high complementary scores with U1-G6C7. Further exploration of the role of U1 complementarity indicated that base pairing at the -4 position also contributed to the response to compound addition. In vitro experiments using fluorescence polarization appear to support this hypothesis. Sequence analysis of the exons impacted by risdiplam or NVS-SM1 revealed a bias for AGA-, AGGA-, and AUGA-ending exons but not CGA-exons. However, expression of U1 snRNA variants (-2U>C, -1C>U) that allow base pairing to all GA exons can promote CGA exon usage, suggesting formation of the A-1 bulge is required for the response to these compounds. To explore the requirement of the bulge A for drug response, the A-1 was replaced with 2-aminopurine, allowing the authors to monitor changes in RNA

structure by fluorescence in vitro. Addition of either U1 oligos (-2U>C, -1C>U) able to base pair with sequence or NVS-SM1 resulted in a marked change in 2-aminopurine fluorescence not seen with WT U1 oligos or inactive forms of the compound alone. Additional experiments determined that NVS-SM1 binding to the A bulge occurred in a sequence independent fashion.
Overall, the authors provide compelling data that increases our understanding of how primary sequence and secondary structure can confer regulation by NVS-SM1 or risdiplam. How this knowledge can lead to more effective development of novel agents for the manipulation of other splicing events is unclear.

Comments
1) Although the authors indicate that RNA abundance impacts the efficiency of particular AGA exons, the argument could be seen as circular. The effect of the compounds is on the precursor RNA (unspliced) whose concentration is not measured in these analyses. Accumulation of RNA is dependent on its appropriate processing and its stablility. RNAs that are not effectively processed are generally degraded. Since abundance of any RNA is dependent on its rate of synthesis versus decay, to make an evaluation of its rate of processing (i.e. splicing), one must first know something about its stability or the level of the precursor RNA.
2) Although the extent of base pairing with U1 snRNA is likely an important determinant in the recognition/usage of a specific 5' splice site, it is not the only determinant in the interaction. Splice site recognition is also impacted by the presence of local splicing enhancers or silencers that enhance or inhibit, respectively, splice site usage. Consequently, use of a weak 5'ss is significantly impacted by the local sequence environment that promotes or inhibits its use beyond just base pairing with U1 snRNA. This point likely underlines the differences in RNAs affected in the two cell lines analyzed (SY5Y vs iNGN2 cells), each likely differing in expression of SR and hnRNP proteins that regulate splicing enhancer and inhibitor activity. The authors should acknowledge this possible contribution and comment as to whether drug affected GA-exons had a higher frequency of adjacent exon splicing enhancer sequences .
3) Fig. 2 A complementary score for AGA exons not included upon NVS-SM1 addition would be informative given the small number of included AGA exon events relative to total AGA exons.

Minor issues
1) The labelling of Fig. 2 is incorrect. According to the legend provided, the violin plot should be labelled as B. Labelling of the y-axis of 2A would be appreciated.
2) Fig. 3E. Y-axis, what is meant by density?
3) Fig. 3F. Legend refers to the wrong data set. CAGA exon is on the left, all other GA-exons is on the right, opposite of what is stated.

Reviewer #4

(Remarks to the Author)
I co-reviewed this manuscript with one of the reviewers who provided the listed reports. This is part of the Nature Communications initiative to facilitate training in peer review and to provide appropriate recognition for Early Career Researchers who co-review manuscripts.

Version 1:

Reviewer comments:

Reviewer #1

(Remarks to the Author)
Referee report for revision of "Specificity and exon target space of splicing modifying compounds" by Lenkeit et al., submitted to Nature Communications

13 March 2026

We thank the authors for their efforts in responding to our critiques. The revised manuscript improves the framing of the study within existing literature, and the quantitative analysis of PSI changes upon drug treatment addresses some of the methodological issues we raised. However, we do not feel that the revision fully addresses our concerns. We still have significant concerns regarding novelty. What new knowledge this contributes about the mechanisms of these drugs remains unclear, and some claims are not fully supported by the data.

Major points

1. It seems to us that the primary contribution of the study is additional empirical support for the requirement of U1–5′ splice site binding for risdiplam- and branaplam-mediated splicing activation, and that this activation occurs via the bulge-repair mechanism. While the bulge-repair mechanism was previously proposed and established via structural studies in Campagne et al. (2019), the present manuscript does provide orthogonal evidence supporting this model. Specifically, the authors' data show that the –4C nucleotide contributes to stabilization of the bulged register, which is mechanistically plausible and, to our knowledge, has not been previously demonstrated.

2. Ishigami et al. (2024) reported that –4A or –3A is required for branaplam binding. In contrast, the authors show only that these nucleotides influence drug activity. They do not test whether these mutations affect U1–5′ splice site binding itself, nor

provide a mechanistic explanation for their effects. This limits the extent to which this work advances the understanding of how these nucleotides contribute to drug activity.

3. The authors also propose that CGA-ending exons (as opposed to [U/C/G]GA) are insensitive to branaplam and risdiplam because these sequences prevent formation of the −1A bulge. However, the supporting evidence is not convincing. Figures 4C–D show that some CGA exons are activated by branaplam and even more strongly by risdiplam (presumably ACGA given Figure 5A,B). Although AGA exons are clearly more responsive than CGA, the differences between UGA and CGA are modest. The authors should directly test whether ACGA-ending exons can form the −1A bulge (as they showed that the U1-C-2U-1 variant closes the bulge) and more clearly demonstrate that ACGA/GUAAGU splice sites (with strong intronic pairing) are not activated by these drugs.

Minor points

1. The experiments intended to validate the requirement of the −1A bulge for drug activity were performed using the U1C-2G-1 variant. However, this mutation is not obviously expected to remove the bulge in the same way that U1C-2U-1 would. Accordingly, the observed reduction in drug effect is minimal, which is consistent with the bulge likely remaining intact. The authors should clarify the motivation for using this variant; if the goal is to test the requirement of the bulged conformation, a mutation predicted to directly eliminate the bulge would be more appropriate.

2. In the correlation plots, many exons appear to have PSI values of 0 or 1 in one of the samples. This suggests that the exon filtering criteria may be too permissive. The authors should consider applying more stringent filtering to exclude poorly covered or constitutively spliced events before computing correlations. While this may not substantially alter the conclusions, it could reduce noise and help clarify patterns of activation or lack thereof.

3. The PCA plot does not include the U1-C-2G-1 ectopic expression experiments, although correlation plots are shown for these samples. For consistency and completeness, these samples should also be included in the PCA analysis or the reason for their exclusion should be explained.

4. The sashimi plots are difficult to interpret and appear to omit some relevant exons. Improving their clarity (e.g., clearer labeling, better scaling, and inclusion of all relevant exons or splice junctions) would make them more informative.

5. The authors have now included a GitHub repository with code for reproducing the analysis. This repository would benefit from additional code and documentation to facilitate reproducibility. In particular, the authors should:

a. Include code used to compute PSI values and perform the statistical analyses (not only the final tables).

b. Provide instructions for downloading the required datasets.

c. Indicate how to obtain or access processed data used in the analyses (refer to the raw data files and processed files deposited at Zotero).

d. Specify the genome annotation version and software versions used throughout.
e. Provide environment specifications (e.g., via a conda environment file) and clear instructions for reproducing the analyses.

(Remarks on code availability)

Reviewer #2

(Remarks to the Author)
I did not have much criticism in the first place, but from what the author added. I am still positive about this work.

(Remarks on code availability)

Reviewer #3

(Remarks to the Author)
The manuscript by Lenkeit et al. has addressed most of the issues raised in my prior review. The current analysis provides important insights into the factors contributing to the effects of risdiplam on RNA splicing seen in their assay systems. Whether these data can be extrapolated to the effect of other compounds on RNA splicing is unclear since the compounds have a very unique mechanism of action (the stabilization of a RNA secondary structure to promote U1 snRNP binding).

Minor Issue.
Fig S4. No mention of panel F is provided in the legend and the x-axis is not labelled.

(Remarks on code availability)

Reviewer #4

(Remarks to the Author)
I co-reviewed this manuscript with one of the reviewers who provided the listed reports. This is part of the Nature Communications initiative to facilitate training in peer review and to provide appropriate recognition for Early Career Researchers who co-review manuscripts.

(Remarks on code availability)

Reviewer #5

(Remarks to the Author)
I co-reviewed this manuscript with one of the reviewers who provided the listed reports. This is part of the Nature Communications initiative to facilitate training in peer review and to provide appropriate recognition for Early Career Researchers who co-review manuscripts.

(Remarks on code availability)

Reviewer #6

(Remarks to the Author)
I co-reviewed this manuscript with one of the reviewers who provided the listed reports. This is part of the Nature Communications initiative to facilitate training in peer review and to provide appropriate recognition for Early Career Researchers who co-review manuscripts.

(Remarks on code availability)

Version 2:

Reviewer comments:

Reviewer #1

(Remarks to the Author)
Referee report for revision of “Specificity and exon target space of splicing modifying compounds” by Lenkeit et al., submitted to Nature Communications (Round 3)

15 April 2026

The second revision does not address our primary concerns. As we stated in our previous report, some of the authors’ results do provide new evidence for the existing understanding of how risdiplam and branaplam activate exon inclusion. However, it remains unclear what novel aspects of drug activity this work uncovers. We still believe that the lack of mechanistic quantitative modeling makes their results fundamentally difficult to interpret. This lack of clarity is exacerbated by how the paper is written.

Regarding specific revisions, we appreciate the additional RNAcofold calculations that the authors report. However, there are discrepancies between their description of these results and the actual figures. More generally, we do not feel that these computational calculations satisfy our concerns: they are simulations, not experiments, and as such they do not account for many of the complexities of splicing in real cells.

Other errors also remain. For example, in Line 86: “a critical C to T substitution deviates the 5’ss of exon 7 from the canonical sequence.” This isn’t true: the C>T mutation is at +6 of exon 7, not in the 5’ss of exon 7.

(Remarks on code availability)

Reviewer #5

(Remarks to the Author)
I co-reviewed this manuscript with one of the reviewers who provided the listed reports. This is part of the Nature Communications initiative to facilitate training in peer review and to provide appropriate recognition for Early Career Researchers who co-review manuscripts.

(Remarks on code availability)

Reviewer #6

(Remarks to the Author)
I co-reviewed this manuscript with one of the reviewers who provided the listed reports. This is part of the Nature Communications initiative to facilitate training in peer review and to provide appropriate recognition for Early Career Researchers who co-review manuscripts.

(Remarks on code availability)

Reviewer #7

(Remarks to the Author)
I have been asked by the editor to evaluate the submission by Lenkeit et al in the context of their responses to issues raised by two rounds or review. In my opinion the manuscript provides valuable information relevant to the mechanisms of selectivity of drugs that modulate 5' splice site recognition. Such compounds have been used therapeutically for the treatment of Spinal Muscular Atrophy or entered clinical trials for the treatment of Huntington Disease. Understanding the basis of splice site specificity (or lack of) of these compounds is of paramount importance for academic and industry research and therefore the results of Lenkeit et al are a valuable contribution to the community.

The concept that drug responses require a bulge repair mechanism was already established by previous reports, including structural data. The results of Lenkeit et al cement the concept that drug responses depend on weak recognition of the 5' splice site by U1 snRNA in the absence of the drug, a concept implicit in some of the previous papers but clearly demonstrated by Lenkeit et al using experiments that modify the sequence at the 5' end of U1 snRNA. In addition, results using a machine learning approach confirm and expand this concept by showing that, while the interplay between U1 snRNA/5' splice site interactions and the presence of bulged out A at position -1 are key, sequence co-dependencies in primary and secondary RNA sequences, regulatory elements and even cell type-specific factors can contribute to drug responses. While some conclusions are based upon predictions and not actual experimental / structural data, the arguments are reasonable in the context of the rest of the work presented. Taken together, the work by Lenkeit et al. provides important information relevant for understanding the mechanism of splicing-modifying compounds with therapeutic potential.

Minor suggestion: including the nucleotide at position 12 at the 5' end of U1 snRNA (a C) in the upper scheme of Figure S5B may help to further visualize the absence of potential base pairing with position -4 in the exon sequence.

(Remarks on code availability)

Open Access This Peer Review File is licensed under a Creative Commons Attribution 4.0 International License, which permits use, sharing, adaptation, distribution and reproduction in any medium or format, as long as you give appropriate

credit to the original author(s) and the source, provide a link to the Creative Commons license, and indicate if changes were made.
In cases where reviewers are anonymous, credit should be given to 'Anonymous Referee' and the source.
The images or other third party material in this Peer Review File are included in the article's Creative Commons license, unless indicated otherwise in a credit line to the material. If material is not included in the article's Creative Commons license and your intended use is not permitted by statutory regulation or exceeds the permitted use, you will need to obtain permission directly from the copyright holder.
To view a copy of this license, visit https://creativecommons.org/licenses/by/4.0/

Reviewer 1

We are very thankful to the reviewer for his/her/their time, work and throughout review of our manuscript. We appreciate the critical feedback, which have led us to analyze our data from another lens and include experiments suggested by the reviewer. Below is a point-by-point response to each of the points raised.

Major points
1. Novelty. The authors do not present a clear view of the existing literature on the specificities of splice-modifying drugs. They also do not adequately discuss the novelty of their findings in light of this literature.

We thank the reviewer for this important comment. To address this concern, we have substantially expanded the introduction to provide a more comprehensive overview of prior studies acknowledging key previous publications and have expanded the discussion section to highlight the contributions of our work in the context of the current knowledge. In addition, we have expanded our study by incorporating a machine-learning (ML)–model that further strengthens the novelty and mechanistic insights of our study.

a. The authors present novel in vitro data, based on measurements of 2-AP fluorescence, that generally support the -1A “bulge repair” mechanism for drug specificity. However, the bulge repair mechanism is not novel – it was proposed by Campagne et al. (2019) based on NMR structural data, and has found further support in the works Malard et al. (2024) and Ishigami et al. (2024).

We thank the reviewer for acknowledging the novelty of our data. Overall, this part of our study is indeed in line with the bulge-repair mechanism reported in literature. Our systematic functional approach, however, enabled us to uncover previously uncharacterized primary sequence co-dependencies that enables a better understanding of this mechanism and how it shapes the largely unexplained target exon landscape of splicing modulators. Specifically,

- **a.** Our data indicates that this secondary structure also forms in the absence of the compound, thereby positioning the −4 position to enable interaction with U1 snRNA and contributing to U1 snRNA–mRNA stability.
- **b.** Using the 1-AP assay, we assessed whether bulge formation is a functional requirement for compound-mediated splicing activation and whether it can be achieved by different primary sequences.
- **c.** We identified that exons that although matching the GA consensus sequence (such as CGA exons), remain non-inducible, likely due to their inability to form the pre-required bulged conformation.
- d. We uncover that splicing modulators have a prevalence to induce exons with two adenines within the last four bases of the exon. Since in some of the structures not

all two adenines contribute to the RNA secondary structure stability, they are likely to favor compound binding.

Thus, while bulge formation itself is not a novel concept, our work provides the systematic functional and sequence-resolved analysis of its necessity for drug responsiveness, thereby substantially advancing mechanistic understanding and improving predictive power for target exons.

b. The authors show that there are additional sequence determinants of the activity of branaplam at positions -4, +6 and +7 of the 5' splice site. This is shown using transcriptomic profiling under ectopic overexpression of modified U1 sequences, as well as in vitro binding experiments. While these confirmatory data are valuable, the importance of the -4 and +6 positions have already been reported (e.g., in Ishigami et al. 2024).

We have expanded our introduction and discussion sections to acknowledge previous work and explain how our data supports earlier findings. Compared to previous studies, we further characterize how the –4 position stabilizes the interaction with the U1 snRNA to enable compound mediated splicing, in several cases compensating for the lack of stability provided by the intronic part of the mRNA. Moreover, we are the first ones, to our knowledge, that demonstrate the modification of a splice modulator's exon target space by mutating the sequence of the U1 snRNA. From a mechanistic perspective, these experiments not only confirm that the intronic positions such as the –6 position are important but also show that their main contribution is to provide stability to the U1 snRNA binding. These experiments also highlight that it is possible to target specific exons with combinations of compound and mutated snRNA treatments.

c. The suggestion that drug activity is influenced by gene expression would, in principle, represent a novel and intriguing finding. But the observed association is more parsimoniously explained by limited statistical power to detect splicing changes in lowly expressed genes due to reduced sequencing depth. Stronger evidence would be required to support this claim.

We thank the reviewer for this comment. To address this point, we performed an additional analysis (shown in the figure below) assessing the relationship between baseline gene expression levels and compound-induced splicing changes. We do not observe a systematic trend between expression level and compound effect size.

We therefore do not claim that compound activity depends on gene expression levels. Rather, our interpretation, now clarified better in the manuscript, is that unexpressed or very lowly expressed genes (for example, genes expressed only in one cell type) cannot be

detected and consequently, cannot show detectable compound-induced splicing changes.

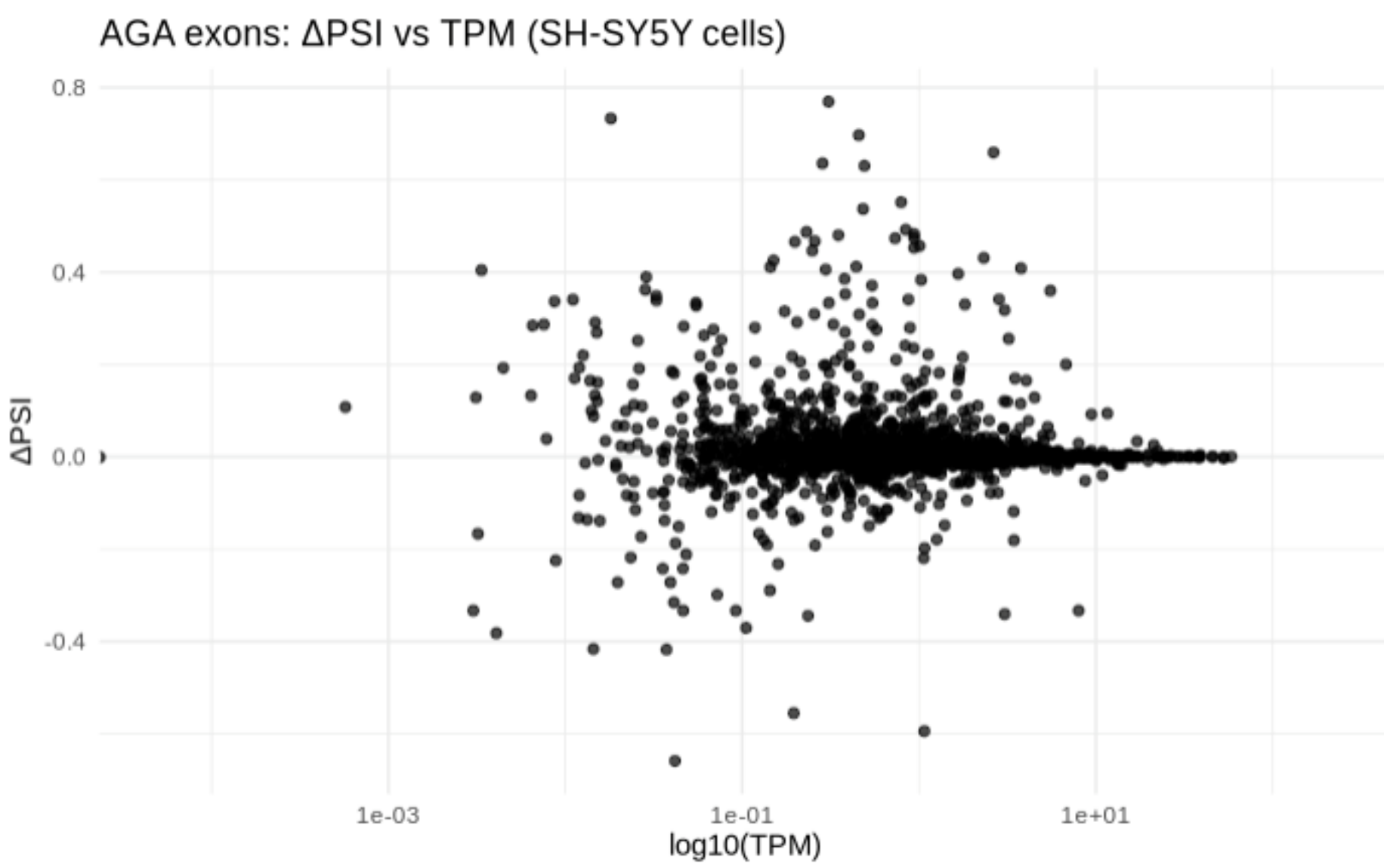

2. Interpretability.
a. The analytical framework used in this study is not well suited to disentangling whether sequence mutations influence drug activity through modulation of the strength of the U1–5' splice site interaction or by altering the strength of drug binding to the U1–5' splice site complex. Prior work has already demonstrated that certain mutations, such as −4A and mutations at +4 and +5, can affect not only basal U1–5' splice site binding but also drug activity. Disentangling these distinct effects requires quantitative modeling, e.g. as carried out in Ishigami et al. 2024. But as manuscript does not present any quantitative modeling, I do not see how these effects can be distinguished.

We thank the reviewer for this comment. In the absence of the compound treatment, our data provides strong evidence that the affinity of the U1-5' splice site interaction is affected by the induced mutations in the U1 snRNA, as we observe strong induction of 5' splice sites that match the induced mutations upon transfection. We also observe these change effects upon compound treatment, as we detect novel nGA-ending exons that match the induced mutations in the U1 snRNA (see Figures 3, S5, S6 and S7).

We agree with the reviewer that we don't analyze if these mutations result in an alteration of the strength of drug binding to the U1–5' splice site. However, we do not see quantitative modeling alone being able to resolve this complex problem. Instead, we think that addressing this question would need several years of development and empirical testing, which is out of the scope for the current study.

However, even in the absence of quantitative modeling data, our results clearly demonstrate exon target space changes upon transfection with a mutated U1 snRNA towards induction of exons that match the respective mutated U1 snRNA bases. Thus, our conclusion that baseline complementarity between snRNA and the target exons contributes to compound activity remains robust.

b. The authors rely primarily on identifying sets of “significantly activated” exons, a strategy that is heavily confounded by variable statistical power across genes. More generally, the lack of statistical significance is repeatedly misinterpreted throughout the manuscript as evidence for the absence of an effect. For example, the claim that AGA at -3 to -1 is insufficient for branaplam sensitivity is based solely on a failure to detect significant differences across all AGA exons. Similarly, the conclusion that branaplam exhibits different sequence-specific effects under ectopic expression of wild-type versus mutant U1 is derived from similar reasoning. While these claims may be true, such arguments are not statistically valid. A more appropriate approach would be to analyze the quantitative effect sizes of drug responses directly, rather than relying on binary significance thresholds.

We thank the reviewer for this comment and agree that observations should be robust independent of analytical approaches. Inspired by the quantitative analyses of Ishigami et al, we have added quantitative analysis showing absolute PSI values comparisons across conditions in addition to only focusing on induced vs non-induced exons based on a significance threshold. These new quantitative analyses confirm the existence of AGA exons that fail to be induced by the compound. We have also added these quantitative analyses for all statistical comparisons, demonstrating the robustness of our results and respective derived conclusions (see Figures 3E, 4C, 4D, 4E, 5, S2C, S3, S5B, S7).

Minor points

1. The authors should provide a more thorough assessment of data quality. For example, scatterplots of estimated exon PSI across biological replicates and across conditions would help demonstrate reproducibility. Similarly, a PCA or related analysis should be used to show that drug treatment accounts for most of the experimental variance between samples.

We thank the reviewer for this suggestion and agree that this helps to demonstrate data quality and reproducibility. We have added scatterplots comparing estimated exon PSI values across biological replicates and across treatment conditions, which show high correlation between replicates (See Figures S10, S11, S12). Additionally, we included the suggested PCA analyses in the Supplementary Material (See Figures S8 and S9).

2. The Methods section should specify the number of biological replicates for each RNA-seq experiment. Line 191 refers to a “single experiment,” but it is unclear whether this means a single biological replicate or a single culture batch.

We are sorry for the confusion and thank the reviewer for this comment. To minimize day-to-day variation and improve direct comparison, all samples were processed on the same day in the experiment mentioned (comparison of different cell lines). All RNA-seq experiments were always performed in biological triplicates, with each replicate representing an independent sample. We added this information in the method section.

3. The manuscript reports that −4C enhances interaction with U1, whereas −4A is suggested to act via direct drug interaction. To strengthen this claim, the authors should show whether −4A affects U1 binding in the absence of drug, analogous to the analysis presented for −4C. This would provide additional orthogonal evidence that −4A does not exert its effect via stabilization of the U1–5' splice site interaction.

We thank the reviewer for this comment. To increase precision of our results, we have clarified the phrasing in the manuscript to reflect that our data cannot full exclude the possibility that −4A does contribute to U1 binding in the absence of compound, however, we reason that it is highly unlikely. Furthermore, we highlight that the requirement for an A at −4, provided −3 is not an A, is consistent with direct compound interaction.

4. The sashimi plots presented for selected exons are interesting, but the generality of these observations should be demonstrated quantitatively. Specifically, scatterplots comparing PSI values under basal and drug conditions in wild-type versus G6,C7 U1 backgrounds should be shown for all exons with complementary 5' splice site sequences. Furthermore, it should be tested whether exons complementary to the wild-type U1 positions 6 and 7 are preferentially activated in the presence of the wild-type U1 compared with the G6,C7 mutant.

We thank the reviewer for this suggestion. We have added the suggested ΔPSI scatterplots comparing wild-type and mutant U1 conditions, highlighting exons that are more complementary to either the wild-type or G6,C7 U1 (Figure 3E and Supplementary Figure S5B). We have also included plots with raw PSI values to quantitatively analyze these effects (Supplementary Figure S7). These plots demonstrate increased activation of AGA exons complementary to the mutant U1 in the presence of this compared to the wild-type U1.

5. The in vitro experiments convincingly show that branaplam stabilizes RNA duplexes only when −1A is bulged out. However, it remains unclear whether this mechanism operates in the spliceosomal context. Additionally, the authors could (for example) test whether ectopic expression of modified U1 containing −2C,−1U not only increases basal PSI but also reduces the effect of branaplam on the splicing efficiency of an AGA exon minigene or endogenous exon.

We thank the reviewer for this comment. Our data show that exons ending in CGA, although they match the GA consensus sequence, are consistently non-induced by

splicing compounds. Using the modified U1 snRNA variant U1C-2U-1, we demonstrate that these CGA-ending exons are *in principle* inducible. This indicates that the lack of induction of CGA exons is a compound-specific effect. Importantly, CGA-ending exons are the only GA-ending exons for which bulge formation at the -1 position is structurally disfavored. The absence of compound-induced activation therefore provides evidence that compounds like NVS-SM1 require an −1 bulged A.

We highly appreciate the reviewer's idea of analyzing changes of compound effects in the presence of U1 snRNA that would disfavor bulge formation in AGA exon and added this additional experiment to the manuscript. In the presence of this mutated U1, the effect of NVS-SM1 on AGA exons is indeed reduced, supporting the idea that bulge formation contributes to compound activity also in the spliceosome.

The reduction is moderate, which we attribute to the fact that endogenous U1 snRNA, capable of forming the bulge, is still present in the cell. This new data has been added to the revised manuscript (see Supplementary Figure S4F).

6. Note all of the raw sequencing data is currently available. Only RNA seq samples from HeLa cells treated with branaplam or ectopically expressing modified −2C,−1U appear to be deposited. Data from experiments performed on SH-SY5Y and iNGN2 cell lines, and with the U1-C6G7 treated HeLa cells, appear to be missing.

We thank the reviewer for pointing this out. All raw sequencing data have been uploaded to the designated repository.

7. The code used to analyze the data should be made publicly available, e.g. on GitHub.
8. The labels for panels B and C are switched in legend of Figure 2B.
Thank you for pointing this out. We have corrected the labelling.

9. Typo: line 116 should read “an NMR structure,” not “a NMR.”
Thank you for the comment. This mistake has been corrected.

10. Line 163: the Snaptron tool should be cited appropriately (Wilks et al., Bioinformatics, 2018).
We thank the reviewer for pointing this out. The Snaptron tool is now properly cited.

Reviewer 2 (Remarks to the Author):

We thank the reviewer for the constructive feedback and for the time dedicated to the revision of our paper.

The authors provide genome-wide sequencing work in order to decipher the mechanism of action of small molecule compounds (risdiplam and branaplam) in enhancing alternative splicing of specific alternative exons. These data confirm the molecular mechanism proposed by the structural work of Campagne et al, but provide these using complementary methods namely RNA sequencing and the modification of U1snRNA. Overall the key element is the presence of a A-1 bulge within a stable RNA duplex. Overall the data are sounds and I cannot think of particular criticism to make. The discussion is rather short and may benefit from mentioning other proposed mechanisms suggested in the literature where an additional motif purine rich is proposed as well for example. How are the data here agree or disagree ?

We thank the reviewer for this suggestion and for pointing out previous studies proposing the involvement of purine-rich sequence elements in mediating responsiveness to risdiplam or branaplam, notably in the context of individual targets such as *SMN2* exon 7 (e.g. Tang et al., 2021). We have expanded the discussion section to better place our results in the context of this existing literature.

In addition, in the revised version of the manuscript, we now analyze the contributions of different genomic elements to exon responsiveness using a machine learning model (now included in Figure 7). Based on these model, we did not observe a conserved purine-rich sequence feature across the genome-wide set of compound-responsive exons. The only consistently constrained region across responsive exons was the U1 snRNA binding site at the 5' splice site, including the −4 position. Based on these observations, we interpret that the purine-rich elements act as exon-specific or context-dependent enhancers for individual exons, rather than representing a general or required determinant of risdiplam or branaplam responsiveness. Furthermore, our model highlights that the sequence context around the U1 binding sites does contribute to exon response upon compound treatment, indicating that different regulatory elements might contribute to the inducibility of each exon.

Reviewer 3 (Remarks to the Author)

We are grateful to the reviewer for the thoughtful feedback and for the time devoted to reviewing our paper.

Comments
1) Although the authors indicate that RNA abundance impacts the efficiency of particular AGA exons, the argument could be seen as circular. The effect of the compounds is on the

precursor RNA (unspliced) whose concentration is not measured in these analyses. Accumulation of RNA is dependent on its appropriate processing and its stablility. RNAs that are not effectively processed are generally degraded. Since abundance of any RNA is dependent on its rate of synthesis versus decay, to make an evaluation of its rate of processing (i.e. splicing), one must first know something about its stability or the level of the precursor RNA.

We thank the reviewer for raising this important point regarding the relationship between RNA abundance, stability, and splicing efficiency. In the first submitted version of the manuscript, we evaluated how rapid degradation of transcripts would prevent us from seeing induced AGA exons. Our analysis indicated that only 8.3% of genes containing undetected AGA exons were downregulated upon compound treatment, which suggested that rapid mRNA degradation would explain only a minority of the uninduced AGA exons. To further assess this concern in the revised version of the manuscript, we knocked down two major RNA degradation enzymes, EXOSC4 and XRN1, and assessed compound mediated exon inclusion under these conditions. The inhibition of these enzymes did not lead to a substantial change in compound-induced exon inclusion, indicating that the large majority observed effects are not primarily driven by differential degradation of misprocessed transcripts, but instead reflect direct modulation of splicing efficiency at the precursor RNA level. The corresponding results and discussion have now been added to the revised manuscript.

2) Although the extent of base pairing with U1 snRNA is likely an important determinant in the recognition/usage of a specific 5' splice site, it is not the only determinant in the interaction. Splice site recognition is also impacted by the presence of local splicing enhancers or silencers that enhance or inhibit, respectively, splice site usage. Consequently, use of a weak 5'ss is significantly impacted by the local sequence environment that promotes or inhibits its use beyond just base pairing with U1 snRNA. This point likely underlines the differences in RNAs affected in the two cell lines analyzed (SY5Y vs iNGN2 cells), each likely differing in expression of SR and hnRNP proteins that regulate splicing enhancer and inhibitor activity. The authors should acknowledge this possible contribution and comment as to whether drug affected GA-exons had a higher frequency of adjacent exon splicing enhancer sequences.

We thank the reviewer for highlighting the potential contribution of local splicing enhancers (ESEs) and silencers (ESSs) in splice site recognition and agree that this is very relevant. We analyzed the frequency of ESE and ESS motifs adjacent to AGA-exons. Interestingly, we did not observe statistically significant enrichment when comparing inducible and non-inducible AGA exons. We speculate that in the initial selection of candidate AGA exons, which needed to be detected in at least one of thousands of human tissue samples, we might be already pre-selecting exons with a certain requirement on the presence of splicing regulators.

However, we found a statistically significant association between the frequency of local and baseline PSI levels of AGA exons in the absence of compound treatment. Specifically, AGA exons with high baseline PSI levels were depleted for ESS and enriched for ESE compared to exons with low baseline PSIs. This result indicates that in normal physiological conditions, the absence of ESS and presence of splicing ESE compensate for weak AGA 5' splice sites.

In addition, we have included a machine learning model to highlight the relative contributions of sequence context, regulatory elements such as ESE and ESS to exon responsiveness and U1 binding site to exon responsiveness.

All these data and observations have been added to the revised version of the manuscript.

3) Fig. 2 A complementary score for AGA exons not included upon NVS-SM1 addition would be informative given the small number of included AGA exon events relative to total AGA exons.

We are assuming that this comment refers to Figure 2C, which displays the complementarity scores. Figure 2C shows the complementary scores for non-included AGA candidate exons. We have changed the description of the figure to make it clearer which exons are shown here. We apologize for the unclear labeling at the beginning.

Minor issues
1) The labelling of Fig. 2 is incorrect. According to the legend provided, the violin plot should be labelled as B. Labelling of the y-axis of 2A would be appreciated.
We thank the reviewer for pointing this out. We have corrected the figure labeling, and we have added an explicit y-axis label to Fig. 2A.

2) Fig. 3E. Y-axis, what is meant by density?
In response to comments from another reviewers, the original density plot shown in Fig. 3E has been replaced by a quantitative scatter-plot analysis that more directly compares exon inclusion changes between canonical and mutated U1 conditions.

3) Fig. 3F. Legend refers to the wrong data set. CAGA exon is on the left, all other GA-exons is on the right, opposite of what is stated.
We thank the reviewer for pointing out this error. The figure legend for Fig. 3F has been corrected.

Reviewer 4 (Remarks to the Author)

I co-reviewed this manuscript with one of the reviewers who provided the listed reports. This is part of the Nature Communications initiative to facilitate training in peer review and

to provide appropriate recognition for Early Career Researchers who co-review manuscripts.

We thank the reviewer for his/her/their contributions.

REVIEWER COMMENTS

Reviewer #1 (Remarks to the Author):

Referee report for revision of “Specificity and exon target space of splicing modifying compounds” by Lenkeit et al., submitted to Nature Communications

We thank the authors for their efforts in responding to our critiques. The revised manuscript improves the framing of the study within existing literature, and the quantitative analysis of PSI changes upon drug treatment addresses some of the methodological issues we raised. However, we do not feel that the revision fully addresses our concerns. We still have significant concerns regarding novelty. What new knowledge this contributes about the mechanisms of these drugs remains unclear, and some claims are not fully supported by the data.

We thank the reviewers for their efforts revising our manuscript. Please see below our responses to each of the points raised.

Major points

1. It seems to us that the primary contribution of the study is additional empirical support for the requirement of U1–5' splice site binding for risdiplam- and branaplam-mediated splicing activation, and that this activation occurs via the bulge-repair mechanism. While the bulge-repair mechanism was previously proposed and established via structural studies in Campagne et al. (2019), the present manuscript does provide orthogonal evidence supporting this model. Specifically, the authors’ data show that the −4C nucleotide contributes to stabilization of the bulged register, which is mechanistically plausible and, to our knowledge, has not been previously demonstrated.

We thank the reviewers for this thoughtful comment. We appreciate the recognition that our study provides new empirical support for the requirements for compound mediated exon inclusion. We are grateful that the reviewer highlighted our demonstration of the contribution of the −4C to stabilizing the bulge formation.

In addition, we would also like to point out the following aspects that have not been discussed previously in literature:

- We demonstrate that baseline complementarity between U1 snRNA and target exons is a key determinant of compound-mediated exon inclusion. Specifically, our results show that a minimal level of complementarity (contributed also by both the

intronic region of the 5' splice site and the −4 position) is required for an exon to respond to the compound.
- By integrating empirical evidence from RNAseq experiments with biochemical assays and genetic perturbation experiments, we go beyond motif characterization and show that not all GA ending exons are equally inducible. Instead, we demonstrate that sequence codependencies beyond the core motif determine drug responsiveness.
- We show that the -1A bulge formation with the U1snRNA is a prerequisite for compound-mediated induction rather than an effect of compound binding.
- We demonstrate that −1A bulge can be formed from diverse primary exon sequences, such as the ACA ending exon shown in Fig. 6, while preserving compound engagement.
- We developed a machine learning model that predicts the response of exons to splicing modulating compounds and use it to systematically evaluate the contribution of regulatory elements to compound response. Our model shows that compound-responsive exons do not contain universal regulatory elements beyond the U1 snRNA binding site. Compound response is instead influenced by subtle, sequence-embedded features whose effects collectively determine the sensitivity of an exon to the compound.
- Based on all these learnings, we can reprogram the specificity of splicing-modulating compounds by genetic manipulation of the U1 snRNA component of the spliceosome.

These results, taken together, advance our understanding of the sequence and regulatory determinants that determine exon responsiveness to splicing modulating compounds, thus providing a comprehensive landscape of the target space of these compounds.

2. Ishigami et al. (2024) reported that −4A or −3A is required for branaplam binding. In contrast, the authors show only that these nucleotides influence drug activity. They do not test whether these mutations affect U1–5' splice site binding itself, nor provide a mechanistic explanation for their effects. This limits the extent to which this work advances the understanding of how these nucleotides contribute to drug activity.

We thank the reviewers for raising this point. We have now directly examined whether −4A influences U1 binding by adding RNAcofold structural predictions to the revised manuscript (Supplementary Fig. S6). These analyses show that the probability of the adenine at position −4 pairing with the corresponding U1 nucleotide can be excluded with high confidence. In detail, this analysis indicates that −4A does not contribute to stabilization of the U1–5'ss duplex and therefore the effects of −4A on drug responsiveness cannot be explained by altered U1 binding. We have updated the results section accordingly to highlight, based on the structural predictions, that the presence of an additional adenine is more likely to promote compound binding, rather than influence U1 recognition.

3. The authors also propose that CGA-ending exons (as opposed to [U/C/G]GA) are insensitive to branaplam and risdiplam because these sequences prevent formation of the −1A bulge. However, the supporting evidence is not convincing. Figures 4C–D show that some CGA exons are activated by branaplam and even more strongly by risdiplam (presumably ACGA given Figure 5A,B). Although AGA exons are clearly more responsive than CGA, the differences between UGA and CGA are modest. The authors should directly test whether ACGA-ending exons can form the −1A bulge (as they showed that the U1-C-2U-1 variant closes the bulge) and more clearly demonstrate that ACGA/GUAAGU splice sites (with strong intronic pairing) are not activated by these drugs.

We thank the reviewers for this comment. In the revised manuscript, we have included RNAcofold predictions to test whether CGA-ending exons can stabilize a −1A bulge in conjunction with U1 snRNA (Supplementary Fig. S5). These predictions indicate that −1A bulge formation is highly unlikely for CGA-ending exons, as the −2G position does not form a bulge-stabilizing base pair with the corresponding C in U1 snRNA.

In the quantitative analyses, the differences between UGA- and CGA-ending exons are modest but not identical. The lower induction observed for UGA-ending exons is consistent with their lower potential to stabilize a -1A bulge compared to AGA-ending exons.

While we acknowledge that these quantitative analyses add some value to our manuscript, they are inherently sensitive to biological and technical variation. When such variability is accounted for using statistical significance thresholds, the data clearly show that CGA-ending exons are refractory to induction by splicing modulators (Fig. S4B).

Minor points

1. The experiments intended to validate the requirement of the −1A bulge for drug activity were performed using the U1C-2G-1 variant. However, this mutation is not obviously expected to remove the bulge in the same way that U1C-2U-1 would. Accordingly, the observed reduction in drug effect is minimal, which is consistent with the bulge likely remaining intact. The authors should clarify the motivation for using this variant; if the goal is to test the requirement of the bulged conformation, a mutation predicted to directly eliminate the bulge would be more appropriate.

We appreciate the reviewers' suggestion regarding the use of the U1C-2U-1 mutant. Based on other experiments presented in this manuscript, however, we know that this U1 variant with perfect complementarity to GA-ending exons massively elevates their basal PSI (see Fig. 4C). As we show in Figure S2 and in the machine learning model, drug-dependent ΔPSI changes are largely masked when basal PSI is extremely high. We therefore decided to use U1C-2G-1, which preserves overall U1-5'ss thermodynamic stability on AGA exons but

disrupts the ability to form the −1A bulge. We acknowledge that this experimental design decision was not clearly described in the previous version of the manuscript and have now clarified it in the revised version of the manuscript.

We have also added RNAcofold analyses demonstrating that the U1C-2G-1 variant has zero pairing probability to form a −1A bulge (Supplementary Fig. S6). Although the −1A nucleotide remains unpaired in this construct, it is not extruded into a bulged conformation. This confirms that U1C-2G-1 specifically eliminates the bulged formation without excessively strengthening U1snRNA–5'ss interactions at AGA exons.

We further note that the observed moderate reduction in compound response is likely due to the presence of endogenous canonical U1 snRNA, as the U1C-2G-1 variant is ectopically expressed rather than mutating the endogenous U1 snRNA.

2. In the correlation plots, many exons appear to have PSI values of 0 or 1 in one of the samples. This suggests that the exon filtering criteria may be too permissive. The authors should consider applying more stringent filtering to exclude poorly covered or constitutively spliced events before computing correlations. While this may not substantially alter the conclusions, it could reduce noise and help clarify patterns of activation or lack thereof.

We thank the reviewers for this helpful suggestion. We followed the recommendation and applied more stringent filtering. We have updated the corresponding figures in the revised manuscript, which now highlight better the high reproducibility of our experiments (Supplementary Figure S13 - 18).

3. The PCA plot does not include the U1-C-2G-1 ectopic expression experiments, although correlation plots are shown for these samples. For consistency and completeness, these samples should also be included in the PCA analysis or the reason for their exclusion should be explained.

We thank the reviewers for pointing this out. We have now included the U1-C-2G-1 samples in the PCA analysis. The revised manuscript contains the updated PCA figure (Supplementary Figure S11 - 12).

4. The sashimi plots are difficult to interpret and appear to omit some relevant exons. Improving their clarity (e.g., clearer labeling, better scaling, and inclusion of all relevant exons or splice junctions) would make them more informative.

Thank you for pointing this out. We have refined the label annotations for all sashimi plots and now explicitly highlight the cassette exon events that each plot is designed to

illustrate.

5. The authors have now included a GitHub repository with code for reproducing the analysis. This repository would benefit from additional code and documentation to facilitate reproducibility. In particular, the authors should:

a. Include code used to compute PSI values and perform the statistical analyses (not only the final tables).

Thank you for the suggestion. We've added the code to calculate the PSI values and to perform the statistical analyses.

b. Provide instructions for downloading the required datasets.

We thank the reviewer for this suggestion and added the information in the README file in the GitHub repository.

c. Indicate how to obtain or access processed data used in the analyses (refer to the raw data files and processed files deposited at Zotero).

We added a section within the README.md file that focuses on downloading and extracting Zenodo-hosted processed datasets.

d. Specify the genome annotation version and software versions used throughout.

Thank you. We have added environment specifications in the *environment.yml* and *renv.lock* files that include details of the software versions used. We added the genome annotation version as well.

e. Provide environment specifications (e.g., via a conda environment file) and clear instructions for reproducing the analyses.

We thank the reviewer for pointing out the missing environment specifications. In the revised version of the manuscript and repository, we have added a conda environment file (environment.yml) for the python scripts and an R environment management via renv, including the renv.lock file. We have also updated the README with instructions on how to recreate both R and Python environments.

Reviewer #2 (Remarks to the Author):

I did not have much criticism in the first place, but from what the author added. I am still positive about this work.

Thank you for the revision of our work, we appreciate the positive endorsement.

Reviewer #3 (Remarks to the Author):

The manuscript by Lenkeit et al. has addressed most of the issues raised in my prior review. The current analysis provides important insights into the factors contributing to the effects of risdiplam on RNA splicing seen in their assay systems. Whether these data can be extrapolated to the effect of other compounds on RNA splicing is unclear since the compounds have a very unique mechanism of action (the stabilization of a RNA secondary structure to promote U1 snRNP binding).

Thank you for revising our manuscript and for the positive feedback. We have added a sentence to the conclusion noting that similar work may be needed to define the target space of other splicing-modulating compounds that act through different mechanisms.

Minor Issue.
Fig S4. No mention of panel F is provided in the legend and the x-axis is not labelled.

We thank the reviewer for pointing this out. We've updated the figure and the corresponding legend.

Reviewer #4 (Remarks to the Author):

I co-reviewed this manuscript with one of the reviewers who provided the listed reports. This is part of the Nature Communications initiative to facilitate training in peer review and to provide appropriate recognition for Early Career Researchers who co-review manuscripts.

Reviewer #5 (Remarks to the Author):

I co-reviewed this manuscript with one of the reviewers who provided the listed reports. This is part of the Nature Communications initiative to facilitate training in peer review and to provide appropriate recognition for Early Career Researchers who co-review manuscripts.

Reviewer #6 (Remarks to the Author):

I co-reviewed this manuscript with one of the reviewers who provided the listed reports. This is part of the Nature Communications initiative to facilitate training in peer review and to provide appropriate recognition for Early Career Researchers who co-review

manuscripts.

Reviewer #1 (Remarks to the Author):

Referee report for revision of "Specificity and exon target space of splicing modifying compounds" by Lenkeit et al., submitted to Nature Communications (Round 3)

The second revision does not address our primary concerns. As we stated in our previous report, some of the authors' results do provide new evidence for the existing understanding of how risdiplam and branaplam activate exon inclusion. However, it remains unclear what novel aspects of drug activity this work uncovers. We still believe that the lack of mechanistic quantitative modeling makes their results fundamentally difficult to interpret. This lack of clarity is exacerbated by how the paper is written.

Regarding specific revisions, we appreciate the additional RNAcofold calculations that the authors report. However, there are discrepancies between their description of these results and the actual figures. More generally, we do not feel that these computational calculations satisfy our concerns: they are simulations, not experiments, and as such they do not account for many of the complexities of splicing in real cells.

We thank the reviewer for the continued evaluation of our manuscript. We agree with the reviewer that computational predictions do not capture the full complexity in cells. Importantly, all our conclusions are supported by experimental approaches, including transcriptome-wide analyses, U1 snRNA perturbations and biochemical assays. RNAcofold complements these experiments by providing additional context for interpreting their results.

Other errors also remain. For example, in Line 86: "a critical C to T substitution deviates the 5'ss of exon 7 from the canonical sequence." This isn't true: the C>T mutation is at +6 of exon 7, not in the 5'ss of exon 7.

We thank the reviewer for pointing out the inaccuracy in line 86. We have corrected this statement to reflect that the C>T substitution in *SMN2* occurs in exon 7 (+6 position) and disrupts splicing regulation rather than altering the 5' splice site sequence.

Reviewer #5 (Remarks to the Author):

I co-reviewed this manuscript with one of the reviewers who provided the listed reports. This is part of the Nature Communications initiative to facilitate training in peer review and to provide appropriate recognition for Early Career Researchers who co-review

manuscripts.

Reviewer #6 (Remarks to the Author):

I co-reviewed this manuscript with one of the reviewers who provided the listed reports. This is part of the Nature Communications initiative to facilitate training in peer review and to provide appropriate recognition for Early Career Researchers who co-review manuscripts.

Reviewer #7 (Remarks to the Author):

I have been asked by the editor to evaluate the submission by Lenkeit et al in the context of their responses to issues raised by two rounds or review. In my opinion the manuscript provides valuable information relevant to the mechanisms of selectivity of drugs that modulate 5' splice site recognition. Such compounds have been used therapeutically for the treatment of Spinal Muscular Atrophy or entered clinical trials for the treatment of Huntington Disease. Understanding the basis of splice site specificity (or lack of) of these compounds is of paramount importance for academic and industry research and therefore the results of Lenkeit et al are a valuable contribution to the community.

The concept that drug responses require a bulge repair mechanism was already established by previous reports, including structural data. The results of Lenkeit et al cement the concept that drug responses depend on weak recognition of the 5' splice site by U1 snRNA in the absence of the drug, a concept implicit in some of the previous papers but clearly demonstrated by Lenkeit et al using experiments that modify the sequence at the 5' end of U1 snRNA. In addition, results using a machine learning approach confirm and expand this concept by showing that, while the interplay between U1 snRNA/5' splice site interactions and the presence of bulged out A at position -1 are key, sequence co-dependencies in primary and secondary RNA sequences, regulatory elements and even cell type-specific factors can contribute to drug responses. While some conclusions are based upon predictions and not actual experimental / structural data, the arguments are reasonable in the context of the rest of the work presented. Taken together, the work by Lenkeit et al. provides important information relevant for understanding the mechanism of splicing-modifying compounds with therapeutic potential.

We thank the reviewer for the careful evaluation of our manuscript and for the positive assessment of our work. We appreciate the recognition that our study provides experimentally supported insights into the determinants of splice site selectivity of splicing-modifying compounds and complements previous structural studies.

Minor suggestion: including the nucleotide at position 12 at the 5' end of U1 snRNA (a C) in

the upper scheme of Figure S5B may help to further visualize the absence of potential base pairing with position -4 in the exon sequence.

We thank the reviewer for the suggestion. We agree that clear visualization of potential base pairing interactions with the U1 snRNA is important. However, we have not extended the schematic to include position 12, as this nucleotide is part of a structured region of the U1 snRNA and is not accessible for base pairing with the pre-mRNA. Including it in the context of the exon–U1 interaction could therefore be misleading, as it might suggest a potential pairing interaction that is not expected to occur in the native spliceosomal context. We believe that the current representation more accurately reflects the biologically relevant interaction interface.
